# Design and Mechanistic Analysis of a Potent Bivalent Inhibitor of Transthyretin Amyloid Fibrillogenesis

**DOI:** 10.1021/acs.jmedchem.5c00430

**Published:** 2025-05-27

**Authors:** P. Patrizia Mangione, Guglielmo Verona, Cristina Cantarutti, Paola Nocerino, Maria Chiara Mimmi, Christopher J. Swain, Diana Canetti, Sofia Giorgetti, Iain Uings, Julian D. Gillmore, Graham W. Taylor, Mark B. Pepys, Vittorio Bellotti, Alessandra Corazza

**Affiliations:** † Department of Molecular Medicine, 19001 University of Pavia, 27100 Pavia, Italy; ‡ Research Department, Fondazione IRCSS Policlinico San Matteo, 27100 Pavia, Italy; § Centre for Amyloidosis, 4919 University College London, NW3 2PF London, U.K.; ∥ Department of Medicine, 9316 University of Udine, 33100 Udine, Italy; ⊥ Transplant Research Area and Centre for Inherited Cardiovascular Diseases, 18631 Fondazione IRCCS Policlinico San Matteo, 27100 Pavia, Italy; # 438592 Cambridge MedChem Consulting, CB22 4RN Cambridge, U.K.; ∇ GSK Medicines Research Centre, SG1 2NY Stevenage, U.K.; ○ Wolfson Drug Discovery Unit, University College London, London NW3 2PF, U.K.; ◆ Istituto Nazionale Biostrutture e Biosistemi, 00136 Rome, Italy

## Abstract

Transthyretin amyloidosis (ATTR) is a systemic disease that primarily affects the heart and the peripheral nervous system. Despite available therapeutic options, advanced ATTR amyloidosis still presents unmet medical needs. We have therefore focused on the design of bivalent small molecules starting from our prototype palindromic ligand mds84, whose binding by transthyretin (TTR) greatly improves stability of the native structure by overcoming the negative cooperativity which is typical of monovalent stabilizers. Among the newly designed compounds here, we present B26, which is pseudoirreversibly bound by native TTR with faster entry kinetics into the protein molecule compared to mds84. It retains the ability to inhibit fibril formation *in vitro*, together with improved solubility. Using solution NMR, we show that B26 occupies both TTR binding sites simultaneously, leading to conformational effects distant from the binding site, including the proteolytic cleavage site involved in fibril formation by the mechano-enzymatic mechanism.

## Graphical abstract

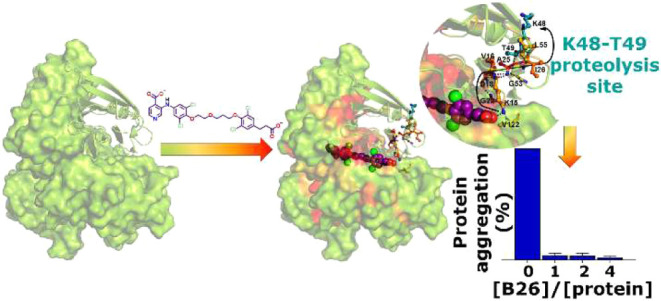

## Introduction

Systemic transthyretin amyloidosis (ATTR) is a progressive, fatal disease in which extracellular deposition of transthyretin (TTR) amyloid fibrils affects different organs, predominantly the heart and peripheral nerves. Initially considered a rare form of disease associated with missense mutations in the TTR gene (ATTRv), ATTR amyloidosis related to the wild-type protein (ATTRwt) is increasingly recognized as a prevalent cause of fatal heart failure with preserved ejection fraction (HFpEF) and/or fatal arrhythmia in the elderly. It occurs predominantly in men, with carpal tunnel syndrome and/or spinal stenosis often preceding cardiac amyloid deposition.

Over the past decade, new therapies have evolved from orthotopic liver transplantation^1^ to novel disease-modifying treatments comprising efforts to reduce plasma TTR concentration and thus amyloid fibril precursor abundance or to stabilize the native tetrameric structure of TTR, thereby inhibiting fibrillar aggregation.

Several agents with varying mechanisms have been approved or are under development as gene silencers, such as small interfering RNA and antisense oligonucleotide.^2^ Patisiran and vutrisiran (RNAi) are beneficial in both neuropathy^3,4^ and cardiomyopathy^5,6^ and inotersen^7^ and eplontersen^8^ are beneficial in polyneuropathy, with eplontersen currently being tested in a Phase 3 clinical trial for cardiomyopathy. Although safe and well tolerated, neither of these therapies completely abolishes plasma TTR, leaving 10–15% of native TTR still circulating.

TTR, originally called prealbumin, owes its name to its function as a carrier protein for the thyroid hormone thyroxine (T4) and retinol. In the cerebrospinal fluid, TTR is the principal T4 transporter, whereas the hormone in the blood circulates mainly bound to thyroxine-binding globulin, a protein with a much higher affinity for T4 than TTR and human serum albumin.^9^ Binding to retinol is mediated by the retinol-binding protein (RBP), which forms a complex that is less susceptible to degradation than RBP alone, making the tertiary complex more effective to maintain a homeostatic level of retinol in plasma.^10^

Native TTR is a noncovalent homotetramer of β-sheet-rich 127-residue protomers assembled as two dimers interacting face to face to create two identical funnel-shaped ligand binding sites for T4 at the dimer–dimer interface, linked by a narrow central channel through the heart of the molecule (Figure 1).

**Figure 1:**
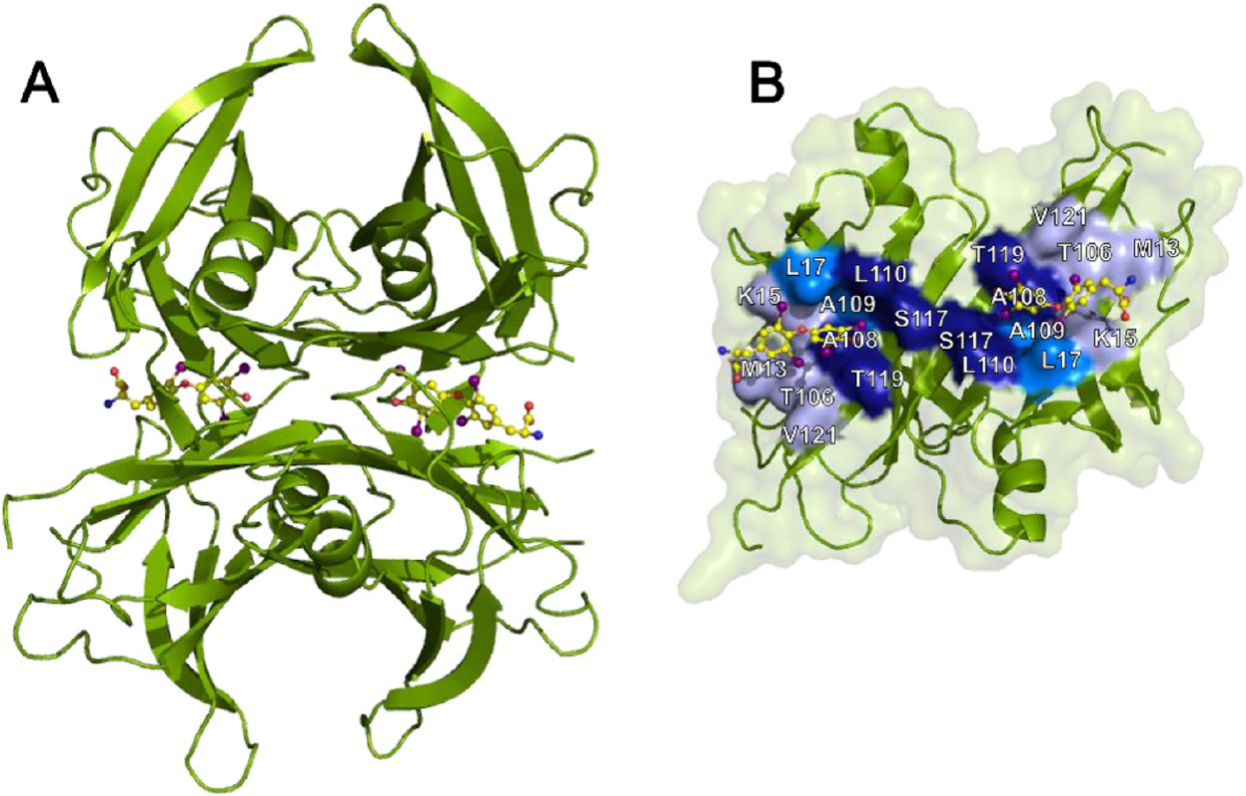
T4 binding pockets of TTR. (A) Cartoon representation of wild-type TTR, with the two bound T4 molecules represented as balls and sticks (pdb: 2ROX). (B) View of a dimer rotated by 90°. With the exception of the binding pocket, the surface is shown in transparent light green. Different shades of blue, from lighter to darker, indicate the binding pockets from the outside to the inside of the channel, named HBP1, HBP2, and HBP3. HBP1 includes M13, K15, L17, T106, A108, and V122; HBP2 includes L17, A108, A109, and L110, and HBP3 includes A108, L110, S117, and T119.

The seminal contribution of Colon and Kelly^11^ focused attention on the dissociation of the tetrameric native structure of TTR into dimers and protomers as a potentially crucial step in the mechanism of ATTR amyloid fibrillogenesis. TTR dissociation was modeled by prolonged exposure of the native protein to low pH. Based on the observation that binding of a single thyroxine molecule by TTR strongly stabilizes the protein against acid denaturation, Kelly and his team invented novel compounds that are specifically bound by TTR in whole human plasma and thereby stabilize the protein against denaturation.

This powerful program led to tafamidis^12^ (Chart 1), a safe, well-tolerated oral drug that has moderate but significant clinical benefit in ATTR patients, and, as the first effective pharmaceutical treatment for this previously untreatable disease, has deservedly enjoyed tremendous success.^13^ The beneficial effects of the recently FDA-approved monovalent stabilizer acoramidis^14^ (Chart 1) (also known as AG10 or Attruby as brand name) on patients with ATTR cardiomyopathy were published in a Phase 3 clinical trial,^15^ expanding the availability of TTR stabilizers. Based on several observations that truncated forms of TTR are present in natural fibrils,^16,17^ we identified a novel mechano-enzymatic mechanism that may underlie TTR fibril formation *in vivo*.

**Chart 1:**
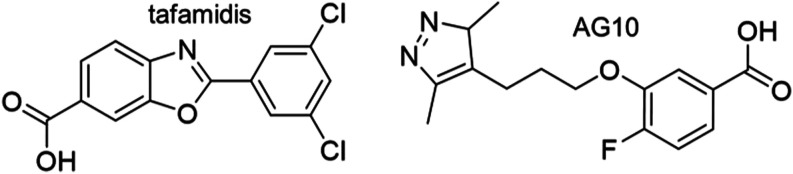
Structures of Tafamidis and AG10

A combination of biomechanical forces and specific serine proteases, operating at normal physiological pH, ionic strength and temperature, releases both amyloidogenic TTR fragments and full length protomers, thereby triggering high yield TTR aggregation into amyloid fibrils.^18,19^ The fibrils have all the distinguishing physical, structural, and biological properties of authentic *ex vivo* ATTR amyloid fibrils. As well as being much more abundant than the rare fibrillar objects produced by acid denaturation, they are more stable than the latter and very closely resemble, both thermodynamically and morphologically, natural ex vivo fibrils.^20^ Inhibition of the mechano-enzymatic pathway of TTR amyloid fibrillogenesis requires occupation of both thyroxine binding sites.^21^ These conditions can be achieved *in vivo* using bivalent stabilizers while monovalent ligands are unlikely to provide full protection *in vivo* due to negative cooperativity.

The discovery of the capacity of our prototype bivalent and palindromic compound, mds84,^22^ to bind pseudoirreversibly TTR under physiological conditions and simultaneously occupy both TTR binding sites provided the first demonstration that a bivalent thyroxine-like molecule could cross the inner channel connecting the two binding sites.^23^ This type of binding offers the highest stabilization of the tetramer, avoiding the negative cooperativity of most of the monovalent ligands. It has also shown the highest potency in inhibiting the mechano-enzymatic mechanism of fibrillogenesis in comparison with other ligands under clinical trials or already available in clinical practice.^21^ However, the unfavorable physicochemical properties of mds84 in terms of molecular weight, lipophilicity, and bioavailability prevented further development as an orally available drug. Nevertheless, it provided the starting point for the design and creation of new bivalent compounds.

Based on the structural requirements and molecular mechanism of action of small molecules that optimally and almost completely inhibit the mechano-enzymatic mechanism of ATTR amyloid fibrillogenesis, we here report the knowledge-based design and characterization of a novel bivalent ligand.

## Results

### Design of a New Family of Bivalent Stabilizers

Since our report in 2010 on the binding properties of a TTR bivalent ligand,^22^ new molecules were designed to maintain the same potency as the prototypic mds84 in inhibiting the mechano-enzymatic mechanism of fibrillogenesis.^18,19^ Key elements of the medicinal chemistry strategy were to reduce molecular weight and calculated lipophilic distribution coefficient, cLogD, by removing atoms not critical to ligand binding based on analysis of the crystallographic data of TTR-mds84 complex (pdb code: 3IPE)^22^ and also to alter the linker to optimize ligand-channel interactions. The first step toward a more drug-like molecule was the substitution of one headgroup of mds84 (Chart 2) with a single ring 3-(3,5-dichlorophenyl) propanoic acid (Head group B) to reduce the molecular weight and lipophilicity.

**Chart 2:**
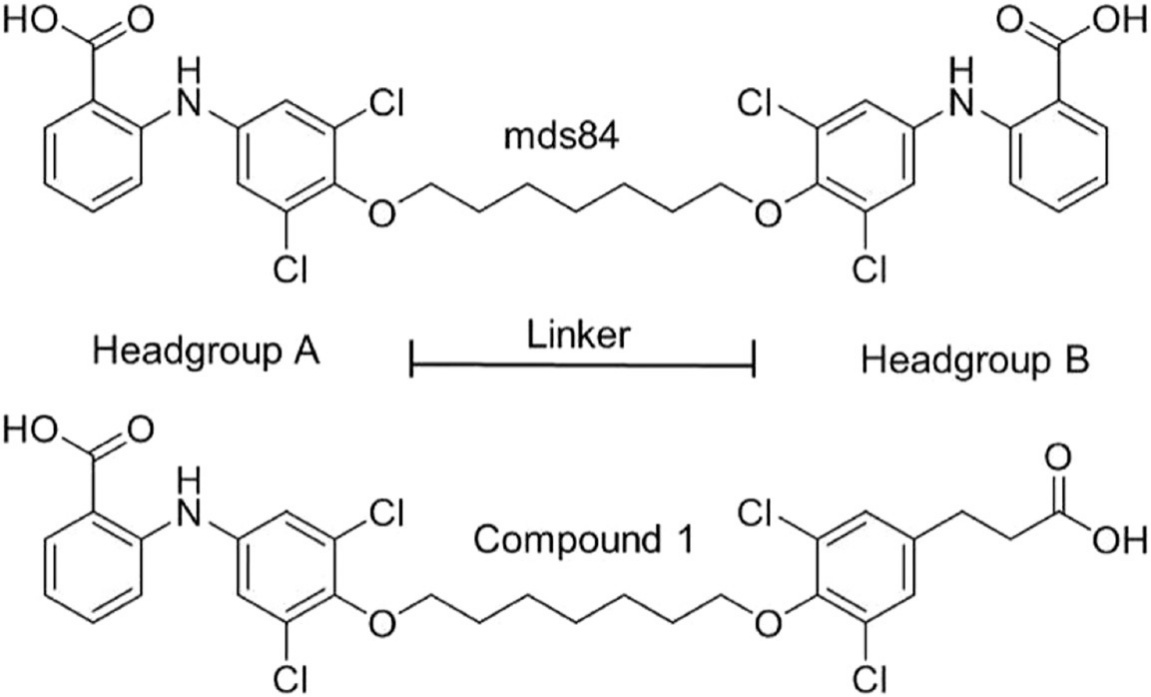
Structures of mds84 and Compound **1**

Encouragingly compound 1 (Chart 2) (MW= 629 and cLogD 3.67) had better capacity to displace ^125^I-thyroxine (^125^I-T4) from TTR in human plasma (pIC_50_ = 2 ± 0.2 μM) and much improved rat pharmacokinetics compared with mds84 (data not shown).

Different compounds were therefore designed by modifying the scaffold according to the following structure-based design principles (Figure 2 and Table 1):

1) *Linker*. Variations of the methylene chain (-(CH2)_7_- in mds84) between the two head groups were rationally designed based on the observation of the X-ray structure of wild-type-TTR bound to mds84. To introduce a potential hydrogen bond interaction between the ligand and the four TTR S117 (one for each chain) positioned in the very center of the TTR channel, we replaced carbons with oxygen in different positions of the linker. In an alternative approach to build hydrogen bonds to S117, hydroxyl substituents were introduced into the alkyl chain. The length of the linker was also changed aiming at the formation of a hydrogen bond (hb) between the headgroup and the side chain of K15, a key point in TTR stabilization as described.^21^
2) *Head group A*. To further reduce lipophilicity, the introduction of heteroatoms in the phenyl carboxylic acid ring (B24, B25, B26, B27) or the removal of one chlorine were explored (B20, B22).
3) *Head group B*. Removal of one chlorine in the phenyl carboxylic ring (B21, B22) was evaluated to reduce lipophilicity. The relevance of headgroup B was also tested by its abrogation in compounds B5 and B6.

**Figure 2:**
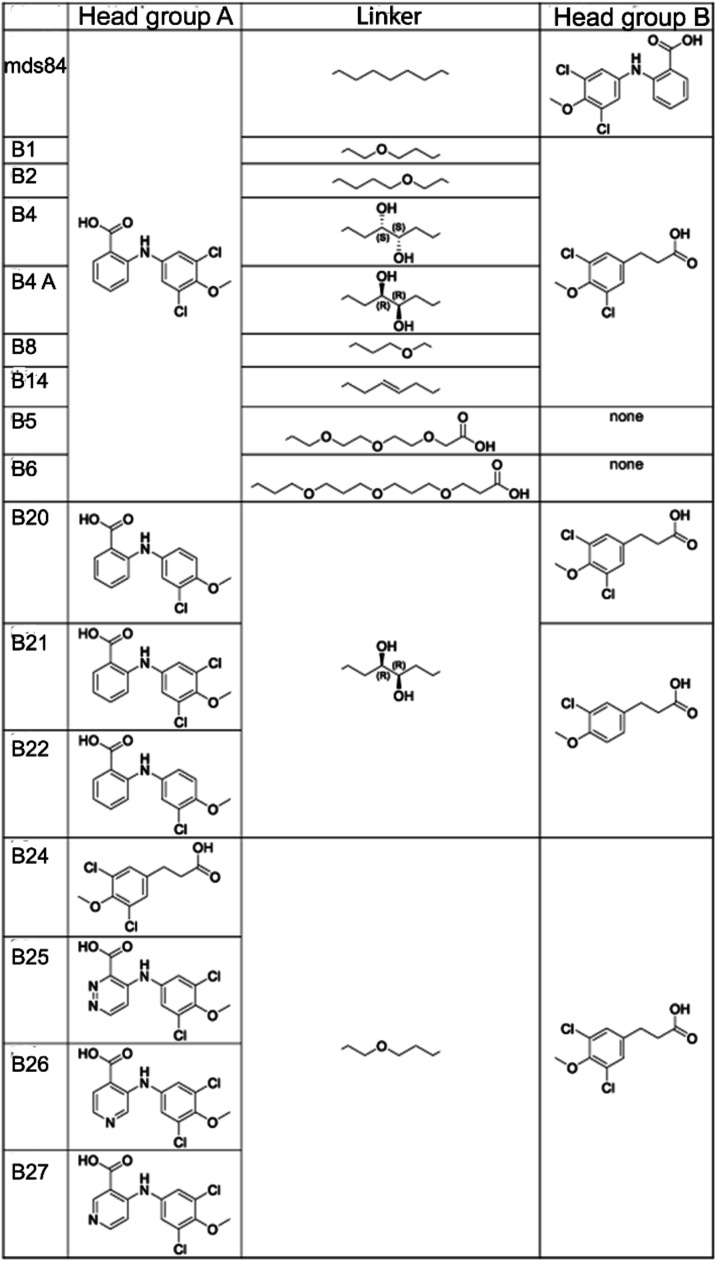
Chemical structures. Linker, head groups A and B of the 15 ligands, shown in comparison with the prototype bivalent compound mds84. All ligands are included in the Patent Application no. WO2022/058733 “Agents for use in the treatment of amyloidosis”.

**Table 1:** Structural and Functional Properties of the Selected Compounds^a^,^b^,^c^,^d^,^e^,^f^,^g^,^h^

compound	cLogD	MW	HBA	HBD	TPSA (Å^2^)	bIC_50_ (μM)	pIC_50_ (μM)	Agg (%)
mds84	6.14	692.41	8	4	117.12	0.15 ± 0.025	19 ± 5.8	2.4 ± 0.30
B1	1.77	617.3	8	3	114.32	0.1 ± 0.03	1.1 ± 0.11	5.2 ± 3.6
B2	2.29	631.32	8	3	114.32	0.1 ± 0.002	2.6 ± 0.59	4.4 ± 1.5
B4A	0.62	647.32	9	5	145.55	0.07 ± 0.009	2.3 ± 0.51	3.3 ± 1.2
B4	0.62	647.32	9	5	145.55	0.07 ± 0.01	4.4 ± 1.2	4.4 ± 3.0
B8	1.87	603.27	8	3	114.32	2.1 ± 0.21	>150	12 ± 3.7
B14	2.86	613.31	7	3	105.09	0.08 ± 0.008	11 ± 2.9	13 ± 3.2
B5	–1.8	488.3	9	3	123.55	5 ± 0.4	66.4 ± 15.7	28.8 ± 13.4
B6	–1.02	544.42	9	3	123.55	1.2 ± 0.41	40 ± 13	12 ± 4.4
B20	0.02	612.88	9	5	145.55	0.07 ± 0.004	7.0 ± 2.6	11 ± 4.1
B21	0.11	612.88	9	5	145.55	0.07 ± 0.004	4.1 ± 2.1	15 ± 2.8
B22	–0.49	578.44	9	5	145.55	0.07 ± 0.01	6.4 ± 2.9	15 ± 2.8
B24	–0.48	619.27	10	3	140.1	0.1 ± 0.005	7.9 ± 1.4	13 ± 8.2
B25	–0.1	619.27	10	3	140.1	0.07 ± 0.01	3.3 ± 1.8	11 ± 4.8
B26	0.45	618.29	9	3	127.21	0.09 ± 0.002	1.4 ± 0.11	4.2 ± 1.9
B27	2.48	618.29	9	3	127.21	0.1 ± 0.02	2.2 ± 0.94	12 ± 1.1

a cLogD: computed coefficient of distribution at pH 7.4.

b MW: molecular weight.

c HBA: hydrogen-bond acceptor count.

d HBD: hydrogen-bond donor count.

e TPSA: topological polar surface area.

f bIC_50_: ligand concentration reducing the binding of ^125^I-thyroxine by isolated recombinant wild-type TTR by 50%.

g pIC_50_: ligand concentration reducing the binding of ^125^I-thyroxine by wild-type TTR by 50% in normal human plasma.

h Agg (%): percentage of the residual V122l TTR fibrillar aggregation in the presence of equimolar concentrations of ligand, based on the ThT emission normalized to the ThT signal of the protein sample without ligand. All functional data are given as mean ± SD of at least three independent experiments.

Activities of 15 representative compounds (Table 1) were evaluated in comparison with mds84 based on their binding properties to both isolated TTR and TTR in human plasma, together with their capacity to inhibit the mechano-enzymatic mechanism of TTR fibrillogenesis.^18,19^

### Synthesis of Bivalent Ligands

The 15 bivalent ligands presented in Figure 2 were successfully synthesized according to the following schemes (Schemes 1–16). For experimental details, see the Experimental Section.

**Scheme 1:**
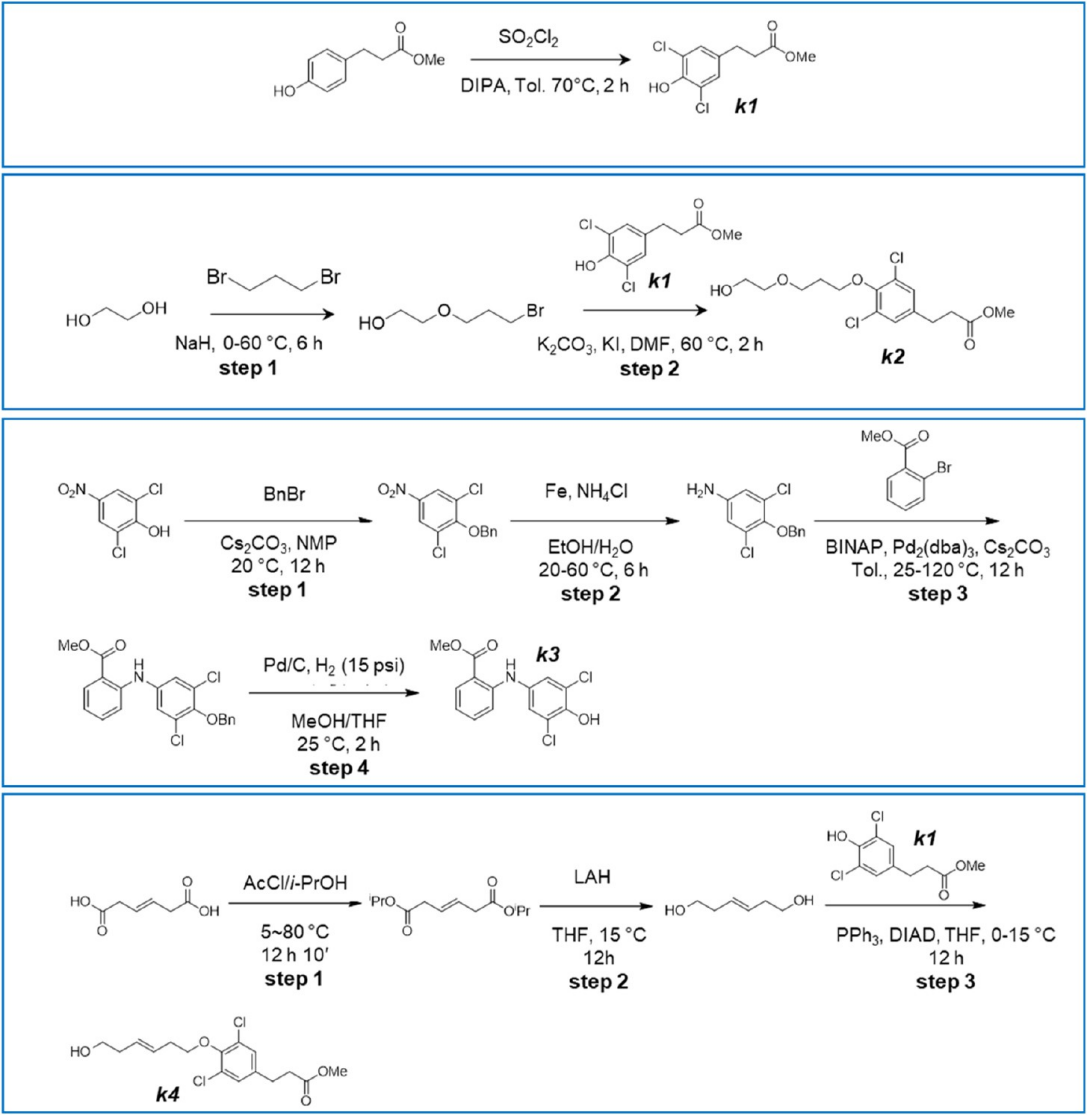
Synthesis of Intermediates *k1*, *k2*, *k3*, and *k4*

**Scheme 2:**
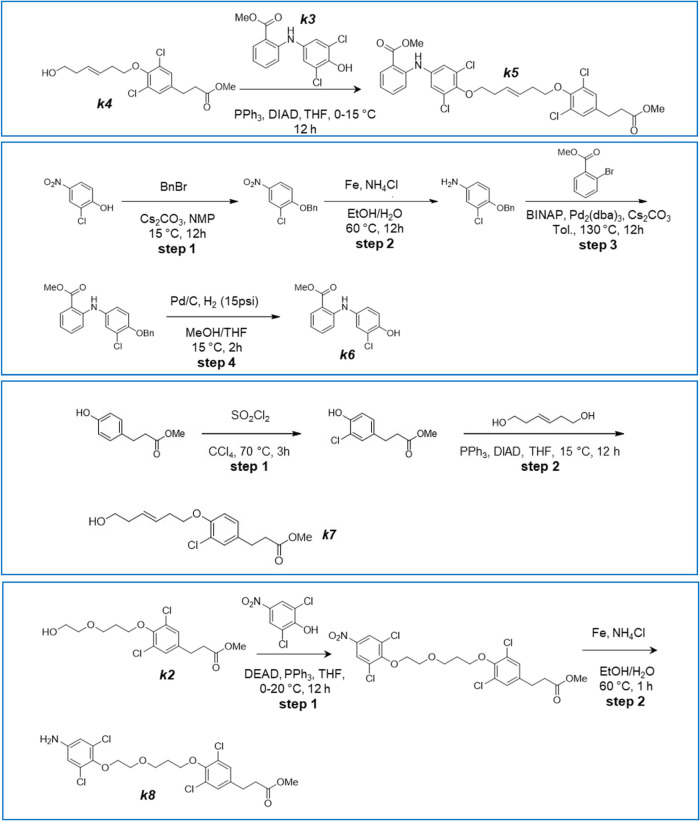
Synthesis of Intermediates *k*5, *k*6, *k*7, and *k*8

**Scheme 3:**
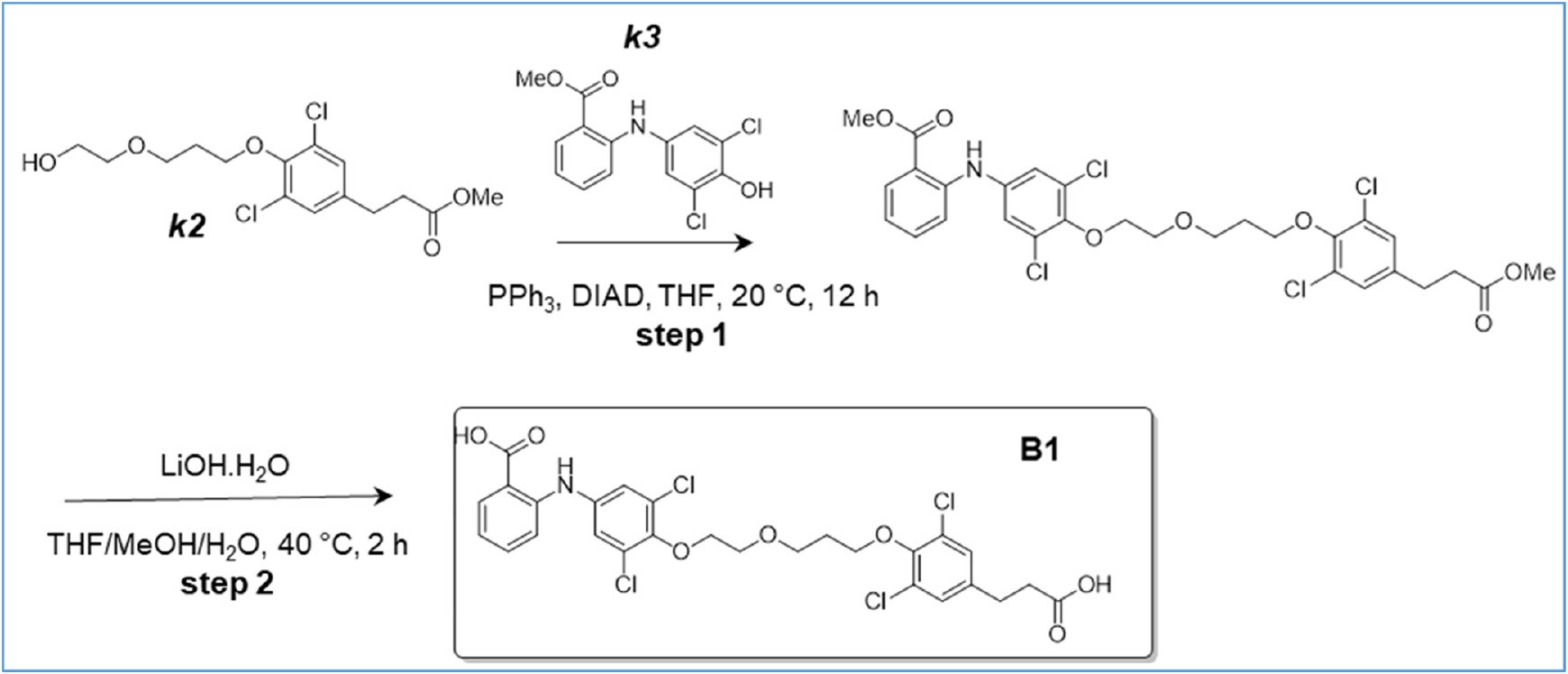
Synthesis of B1

**Scheme 4:**
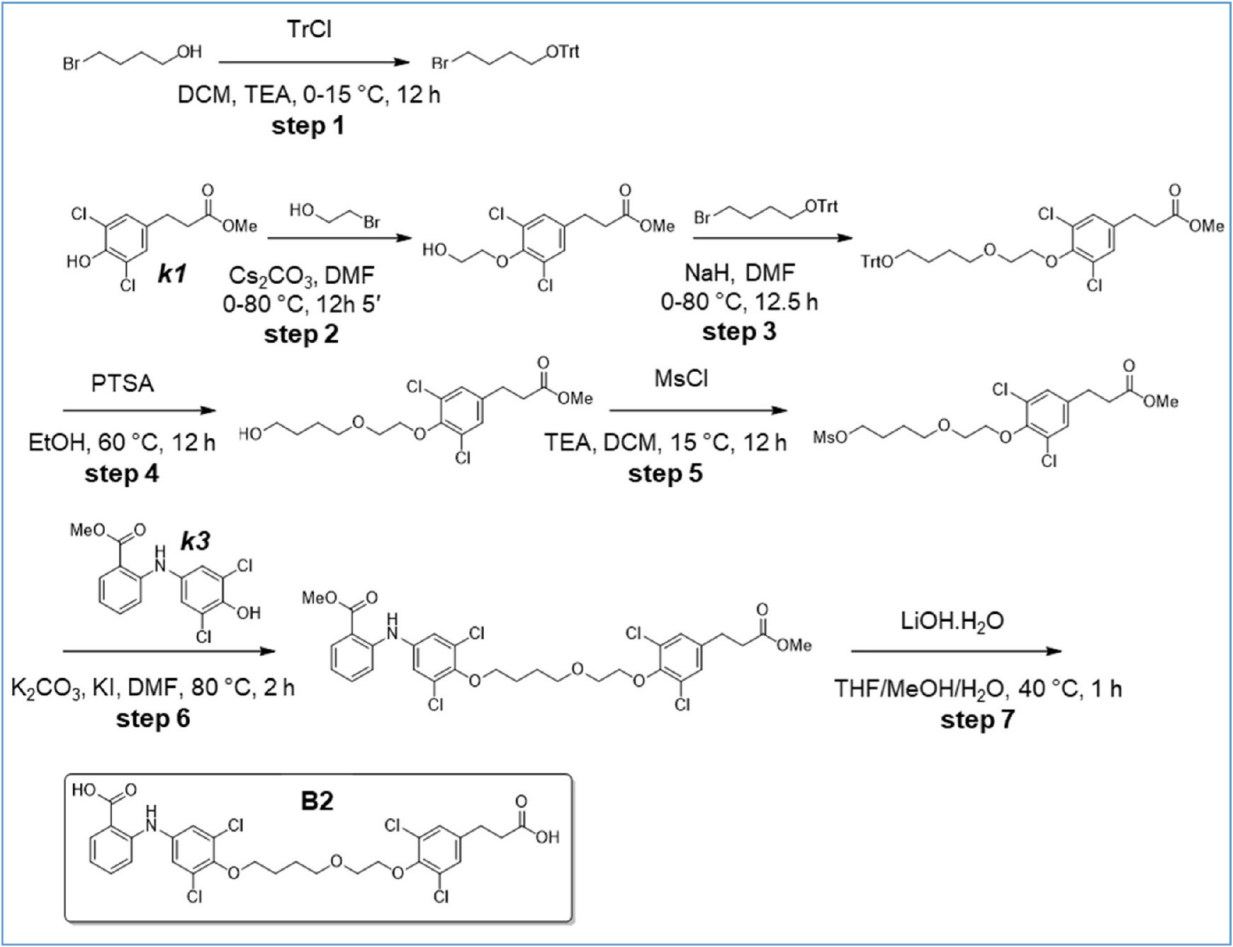
Synthesis of B2

**Scheme 5:**
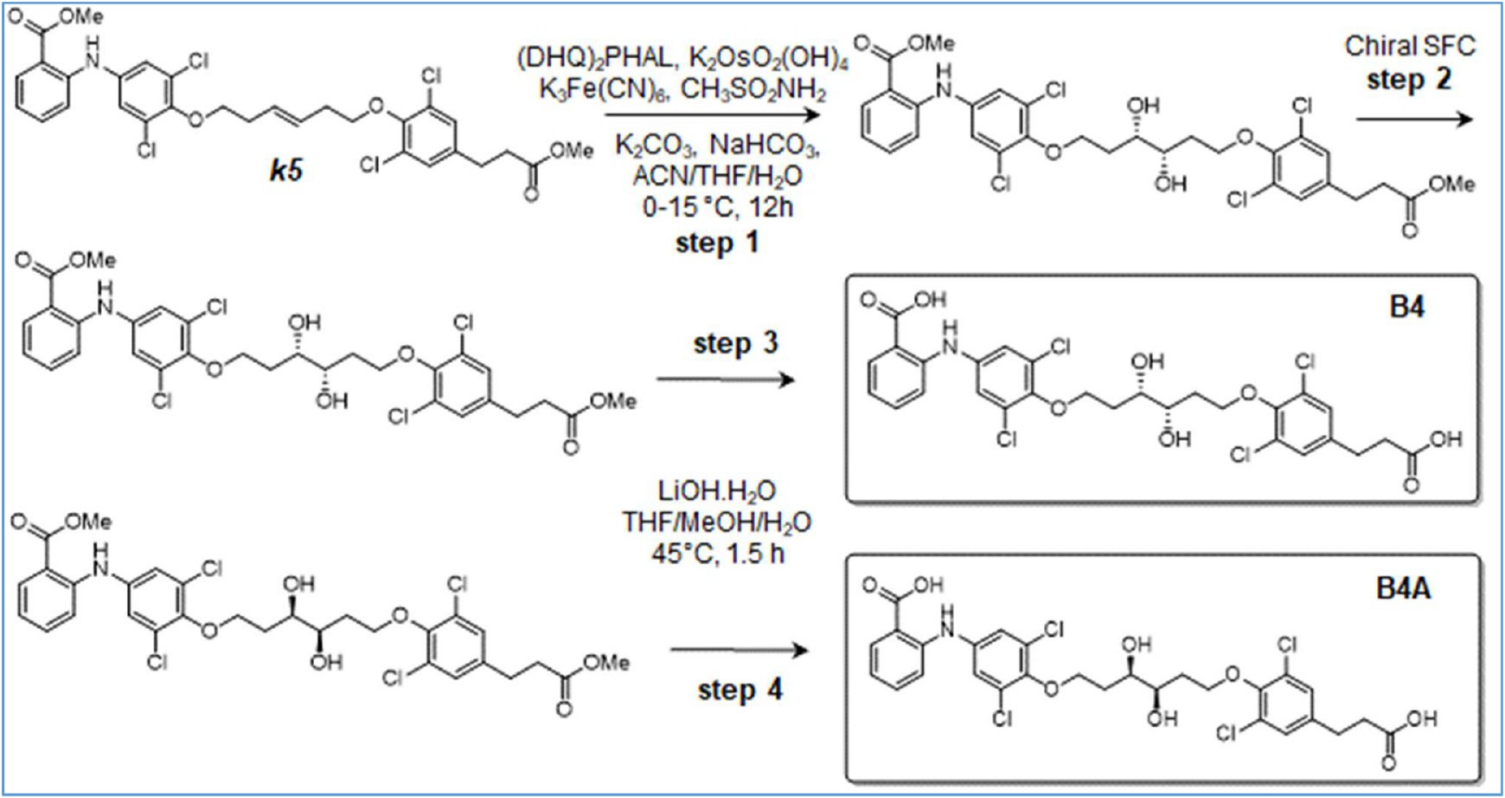
Synthesis of B4 and B4A

**Scheme 6:**
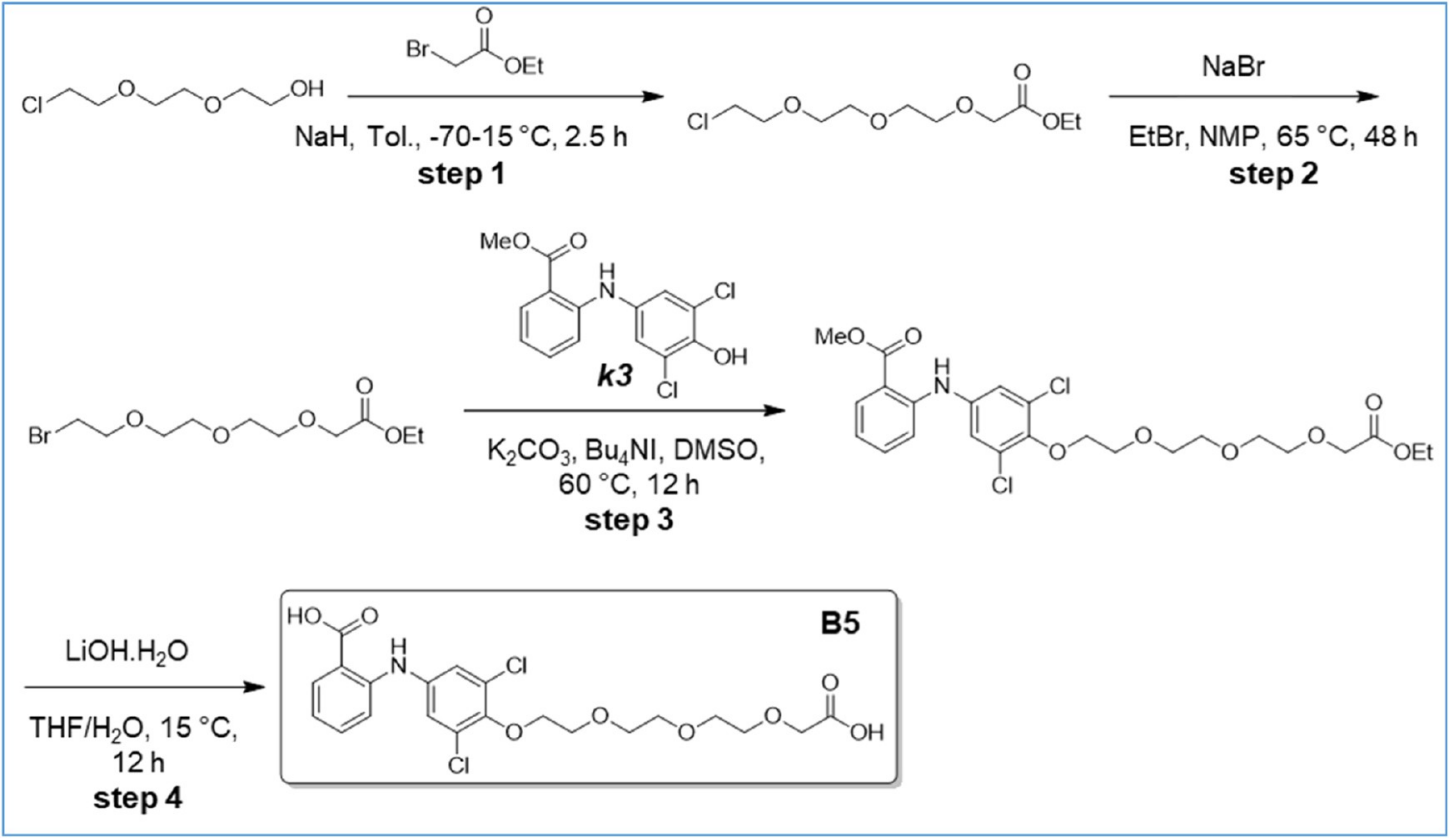
Synthesis of B5

**Scheme 7:**
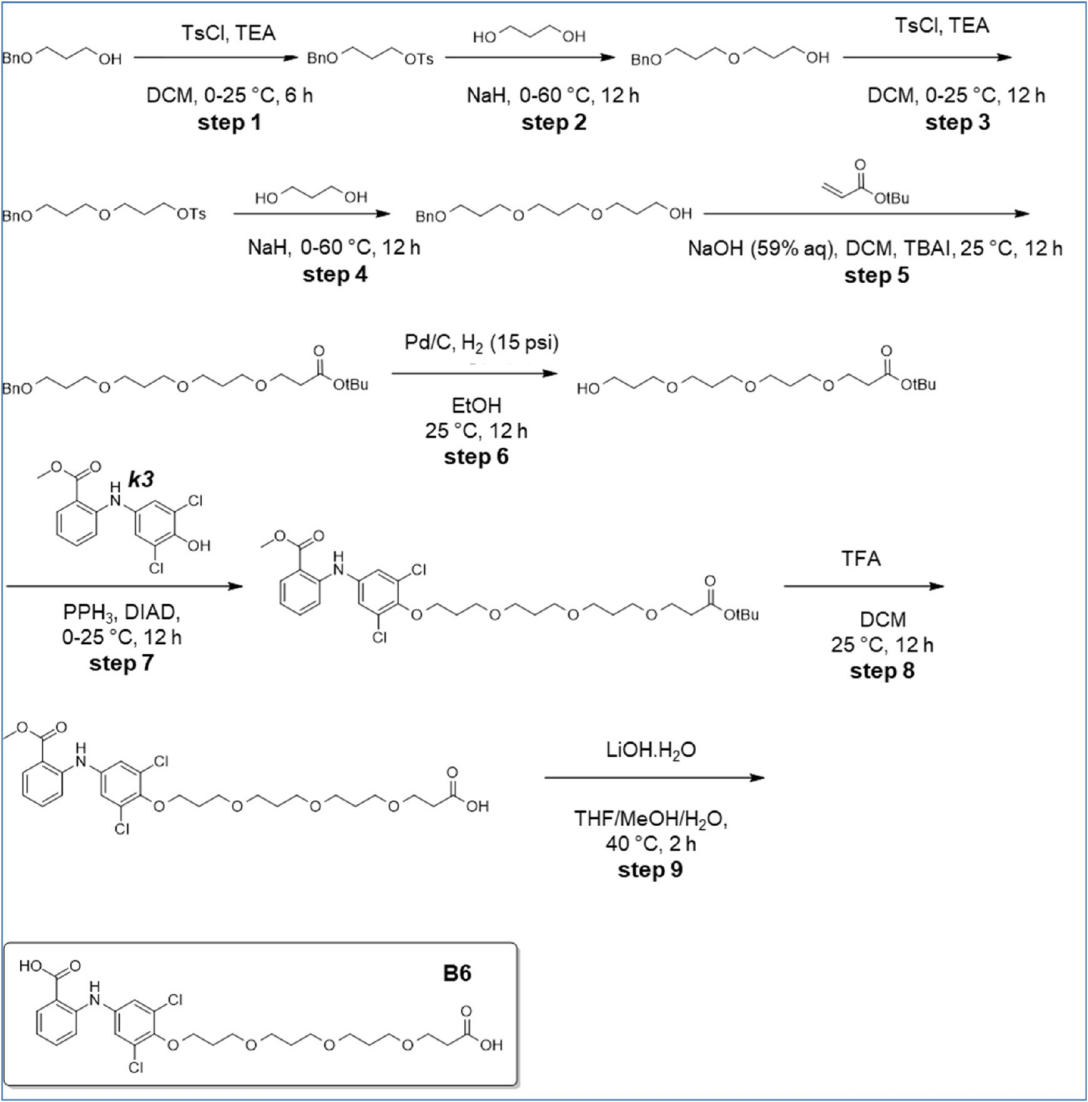
Synthesis of B6

**Scheme 8:**
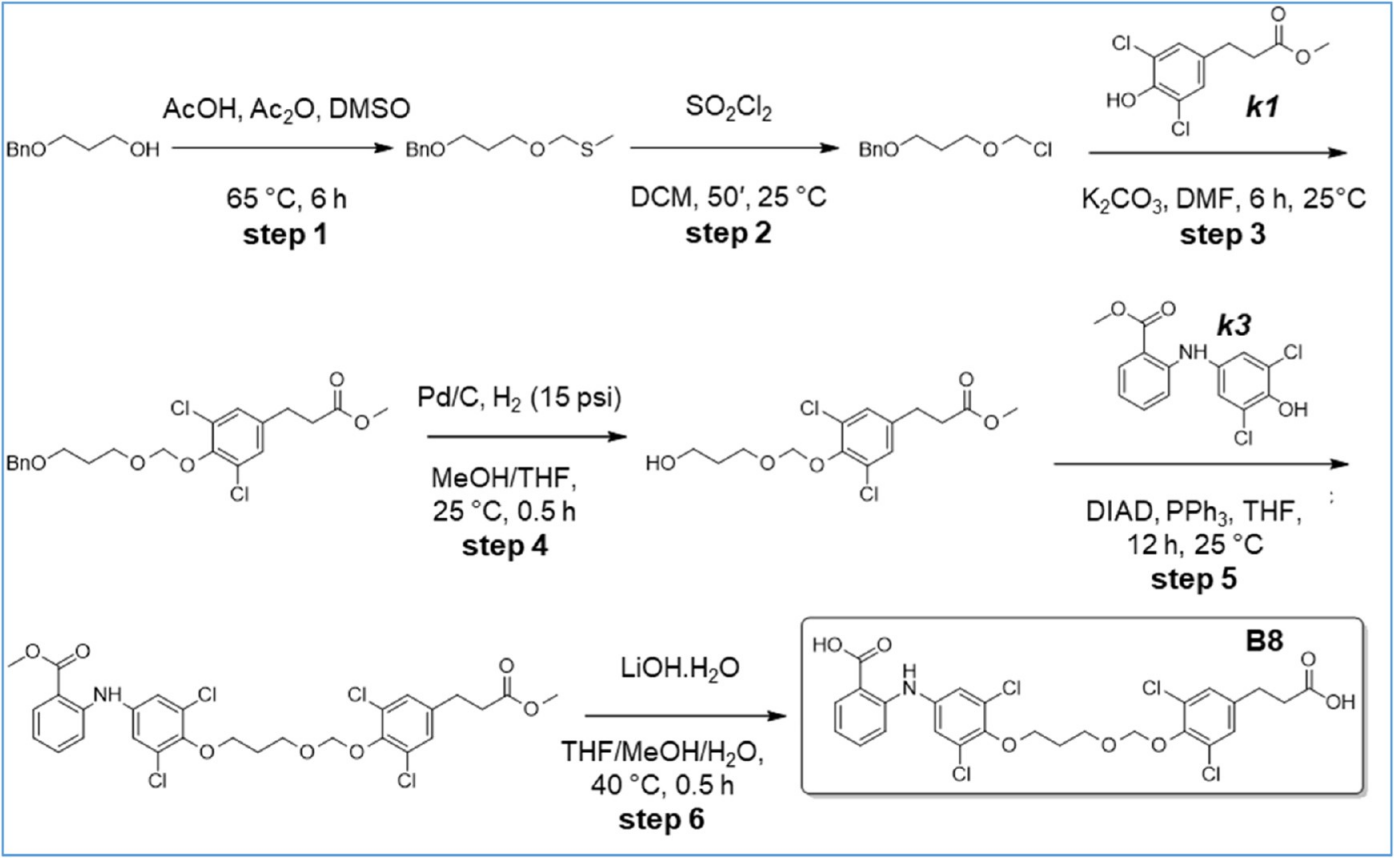
Synthesis of B8

**Scheme 9:**
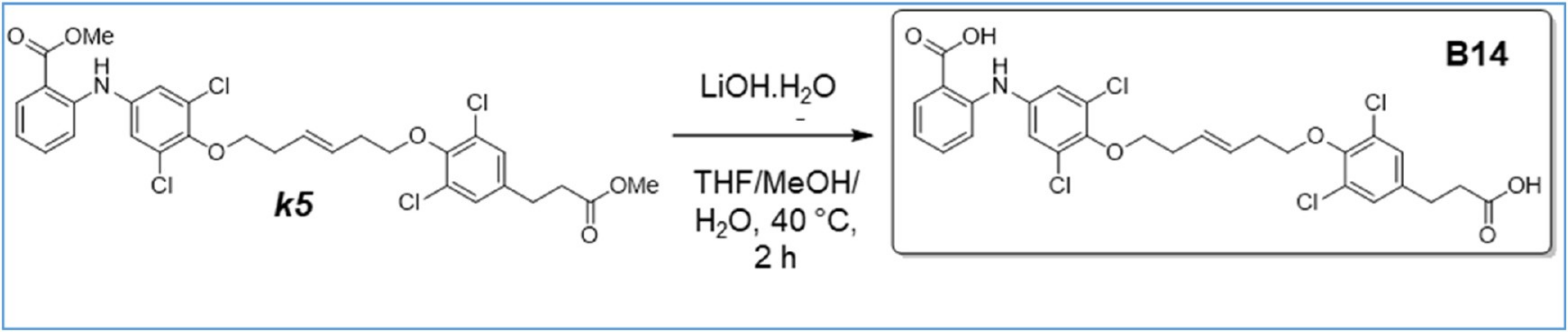
Synthesis of B14

**Scheme 10:**
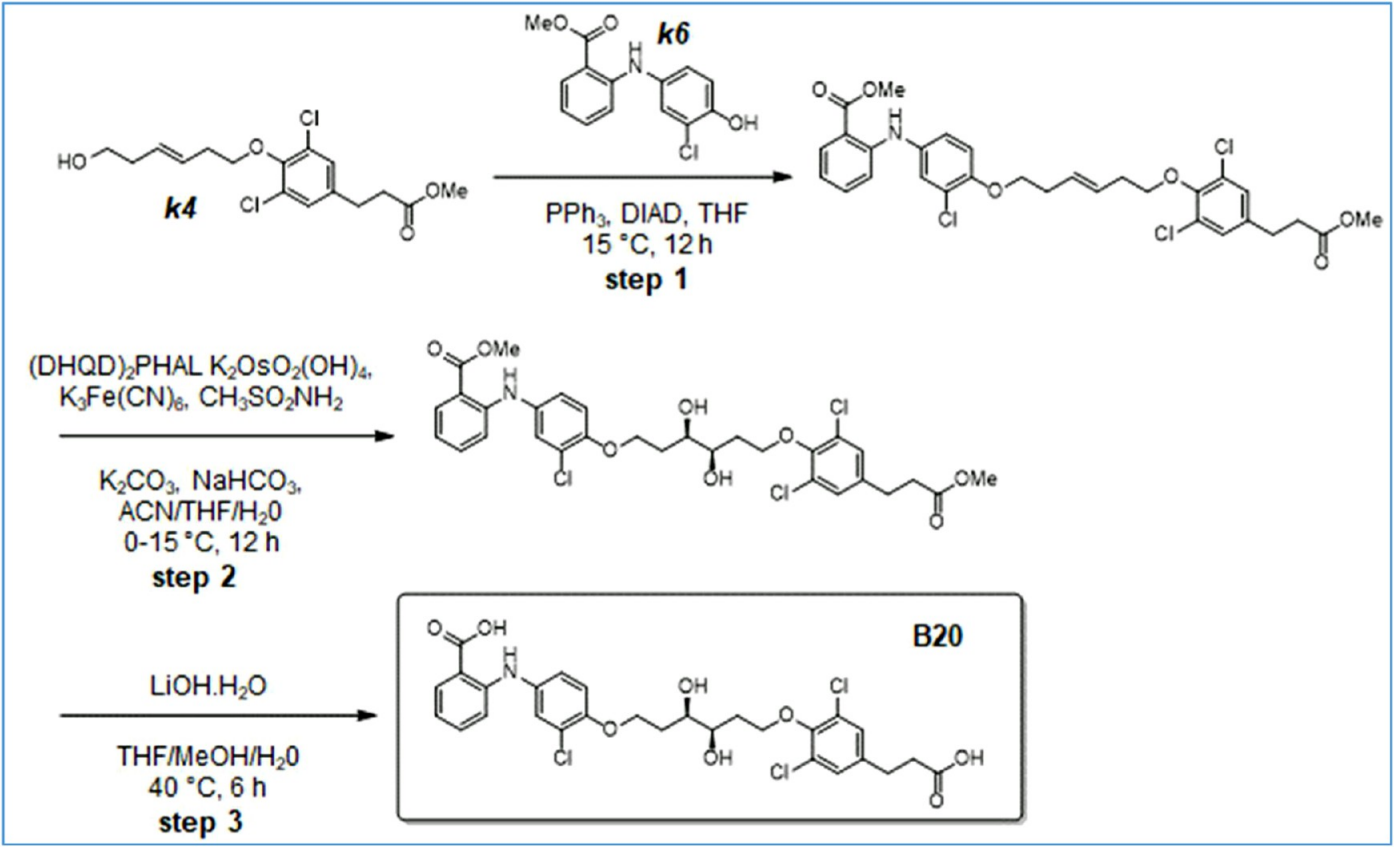
Synthesis of B20

**Scheme 11:**
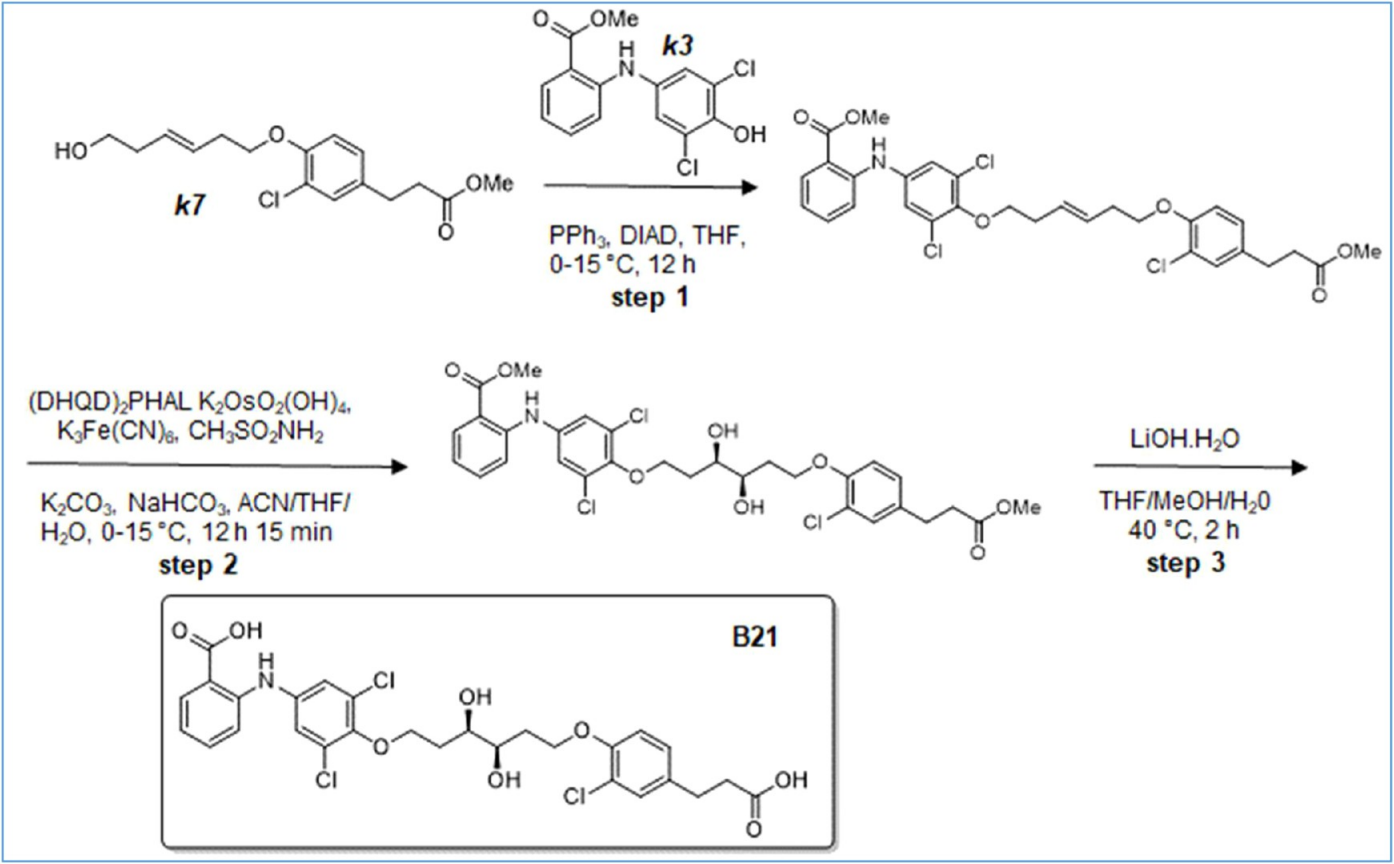
Synthesis of B21

**Scheme 12:**
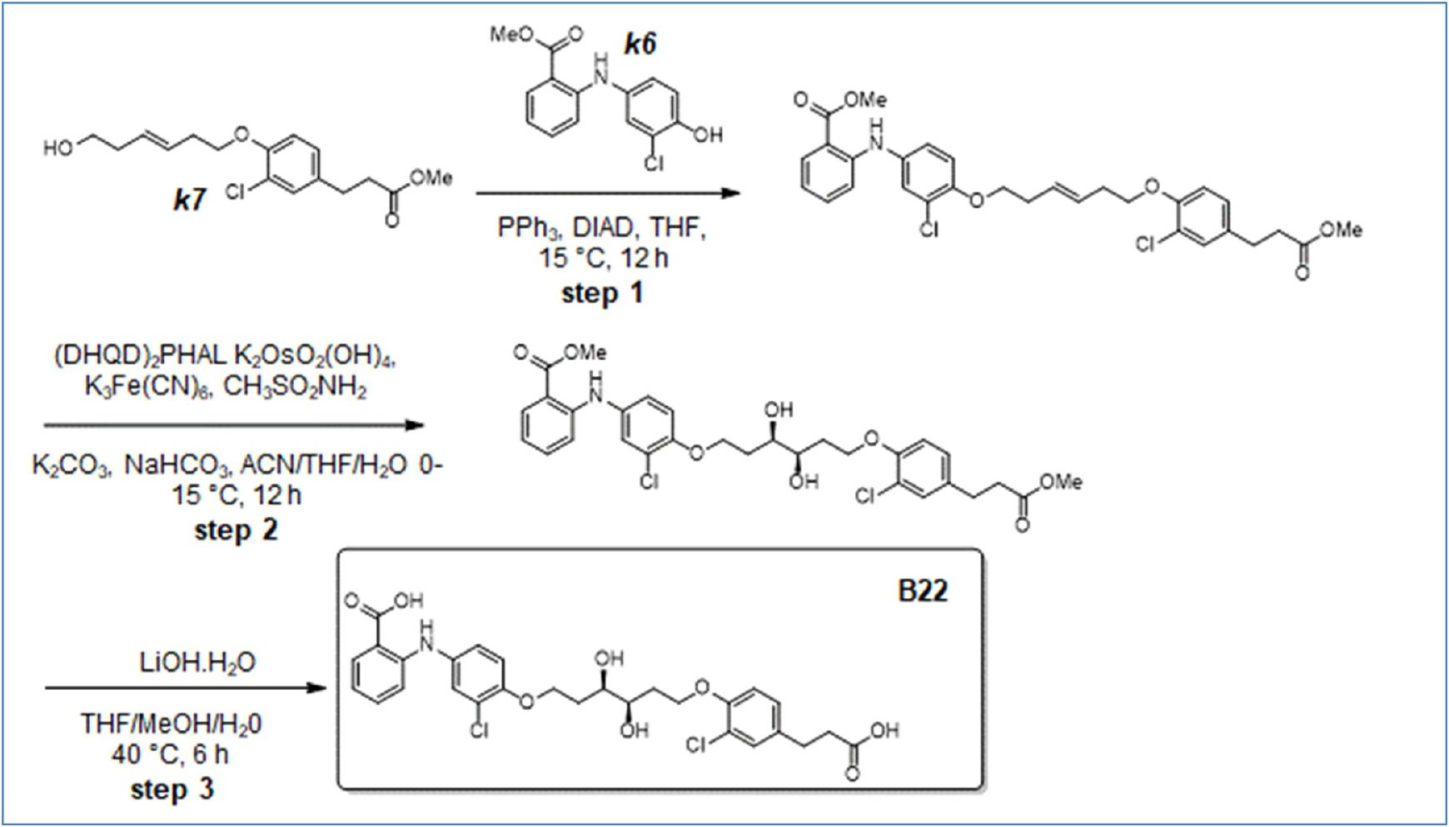
Synthesis of B22

**Scheme 13:**
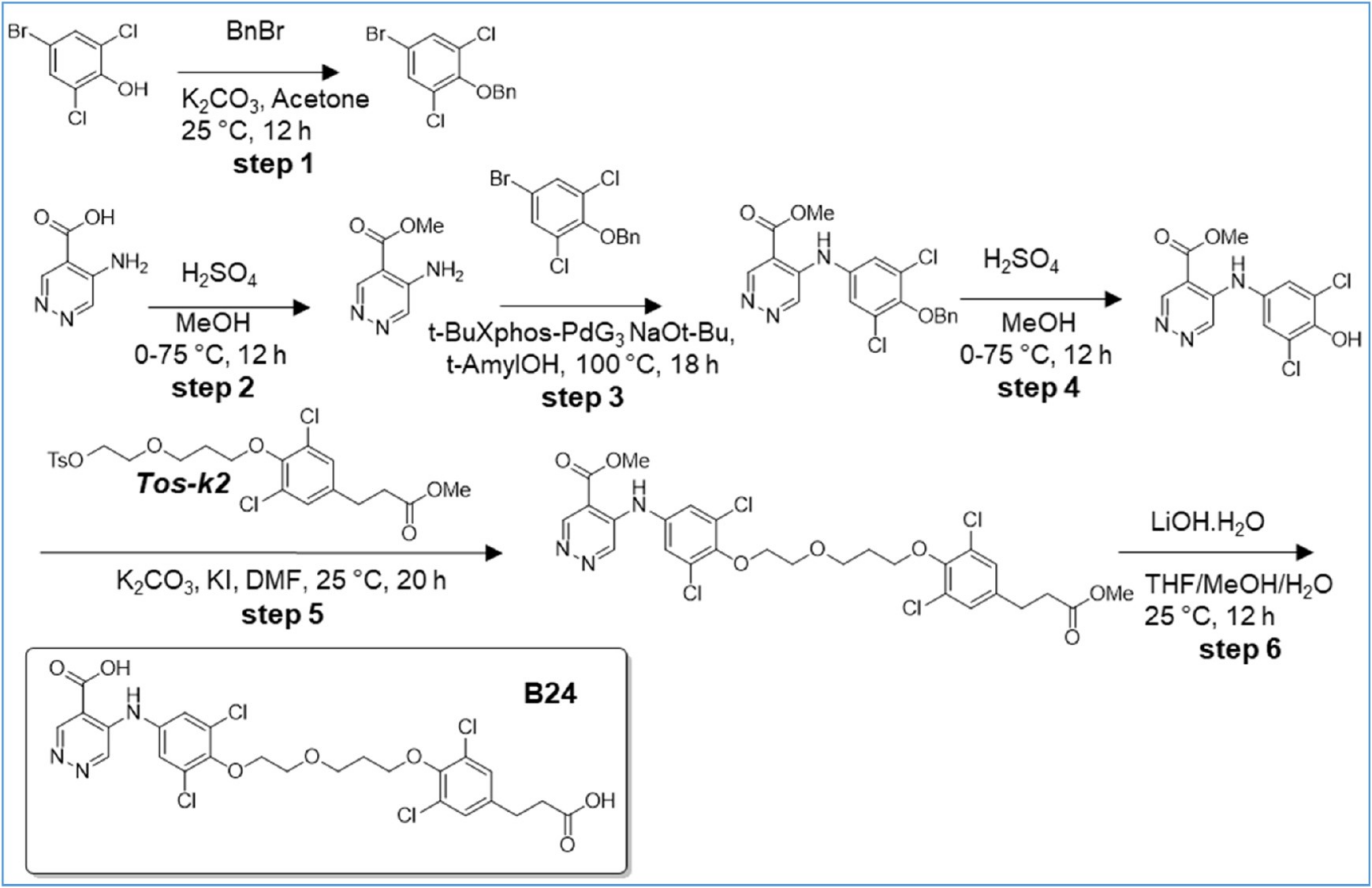
Synthesis of B24

**Scheme 14:**
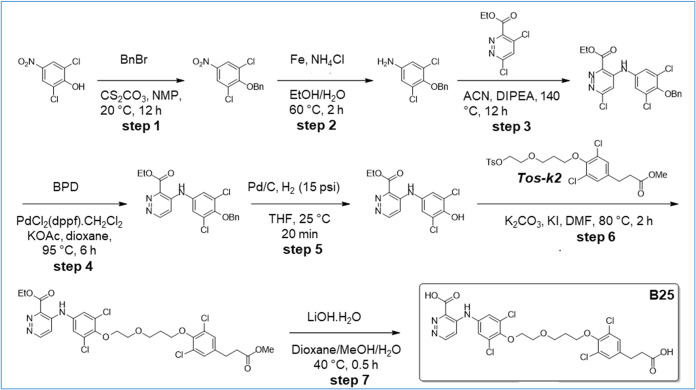
Synthesis of B25

**Scheme 15:**
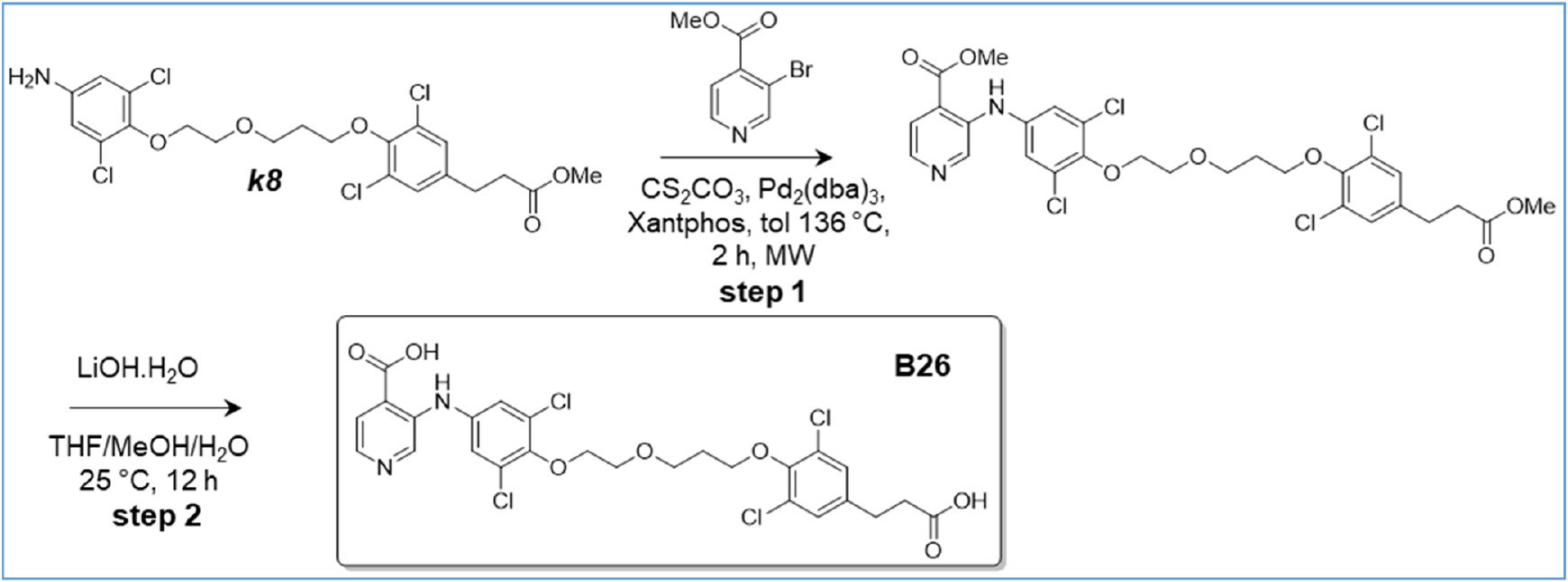
Synthesis of B26

**Scheme 16:**
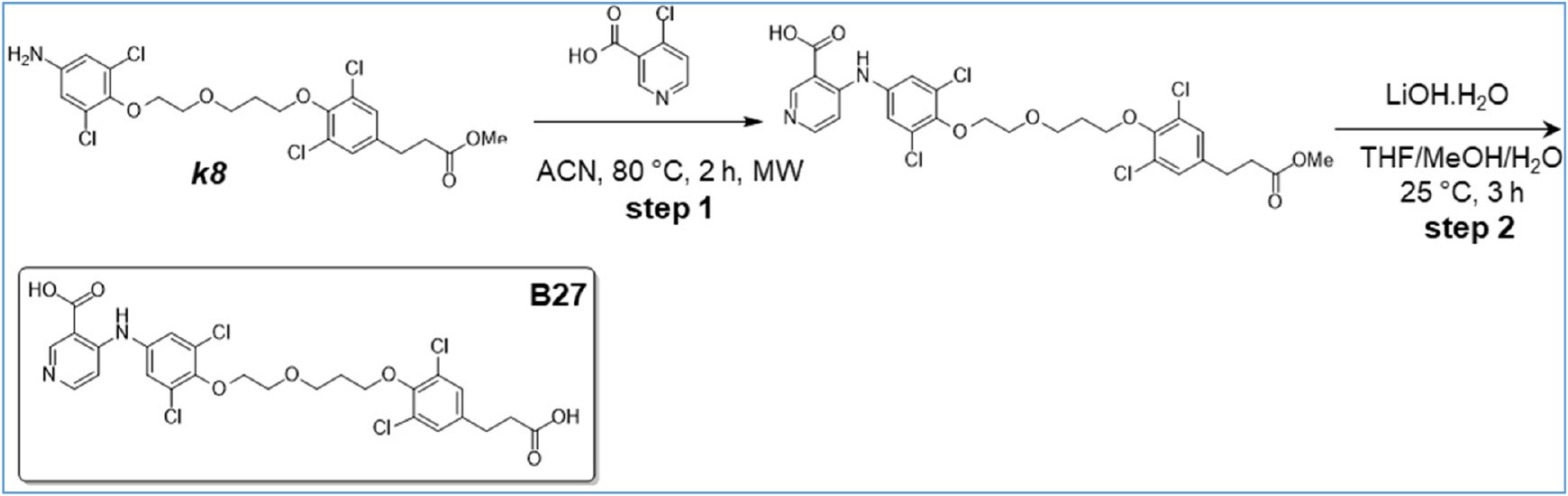
Synthesis of B27

### Ligand Binding by Isolated TTR

Assessment of binding was based on the ligand capacity to displace ^125^I-T4 from isolated recombinant wild-type TTR.^22^ As shown in Table 1, complete removal of the headgroup B (B5, B6) as well as the introduction of a shorter linker, −(CH_2_)_3_OCH_2_– in one of the bivalent compounds (B8) negatively affected the binding by TTR leading to higher values of IC_50_ in buffer (bIC_50_) compared to mds84. Changes in head groups A, removal of chlorines in A and/or B as well as most modifications in the linkers did not appear to affect the capacity to displace the radiolabeled T4.

### Ligand Binding by TTR in Normal Human Plasma

All the compounds were further evaluated to test their capacity to displace ^125^I-T4 from TTR in normal human plasma. The three compounds that showed reduced TTR binding in buffer (B5, B6, B8) also showed reduced binding in the plasma assay.

The replacement of one of the two head groups of mds84 with a single ring 3-(3,5-dichlorophenyl) propanoic acid, coupled with the introduction of hydrophilicity in the linker region, increased the potency of binding to TTR in plasma compared to mds84 (B1, B2, B4, B4A). Removal of chlorines from headgroup A and/or B of compound B4A (B20, B21, B22) led to a reduction in the observed potency in plasma (Table 1). Modification in the B1 molecule with the introduction of heteroatoms in headgroup A resulted in a slight reduction of binding potency for B24 and preservation of the properties for the others (B25, B26, B27) (Table 1).

### Inhibition of the Mechano-Enzymatic Mechanism of Fibrillogenesis

To test their potency as inhibitors of TTR fibril formation, the 15 ligands were evaluated for their ability to inhibit the mechano-enzymatic mechanism of fibrillogenesis of V122I TTR, which is one of the most common amyloidogenic variants,^19,21^ causing a disease with severity intermediate between the wild-type and other rare, more aggressive types of amyloidosis.^24^ As shown in Table 1, most of the molecules directly derived from compound 1 after modification in the linker (B1, B2, B4, B4A) were excellent inhibitors of fibrillogenesis. Antifibrillogenic activity was reduced in other derivatives of compound 1 in which the linker was shorter (B8, B14) or headgroup B was removed (B5, B6). Removal of chlorines in headgroup A and/or B resulted in reduced potency (B20, B21, B22). Changes in headgroup A maintained excellent antifibrillogenic activity in B26, in which the 4-carboxy-pyridine seemed to inhibit better than the 3-carboxy-pyridine (B27), the 4-carboxy-pyridazine (B24), and 3-carboxy-pyridazine (B25). Biochemical activity of ligands together with their physicochemical properties (i.e., cLogD, TPSA) were taken into consideration and B26 was finally selected as the most promising lead for further studies (Figure 3A). An additional analysis was carried out to compare the efficacy of the bivalent B26 with monovalent tafamidis^12^ and AG10 ^14^ (Figure 3B) as inhibitors of *in vitro* V122I TTR fibril formation at different molar ratios of ligand/protein. Prototype mds84 was also included for comparison. Equimolar concentrations of both bivalent ligands showed a similar effect as they almost completely inhibited TTR amyloid formation *in vitro* compared with the significantly lower inhibitory effect of both tafamidis and AG10 (Figure 3C). Although higher molar ratios of the monovalent ligands did inhibit fibrillogenesis, this cannot be replicated *in vivo* because the negative cooperativity of TTR cannot be overcome at feasible drug dosage.^25,26^

**Figure 3:**
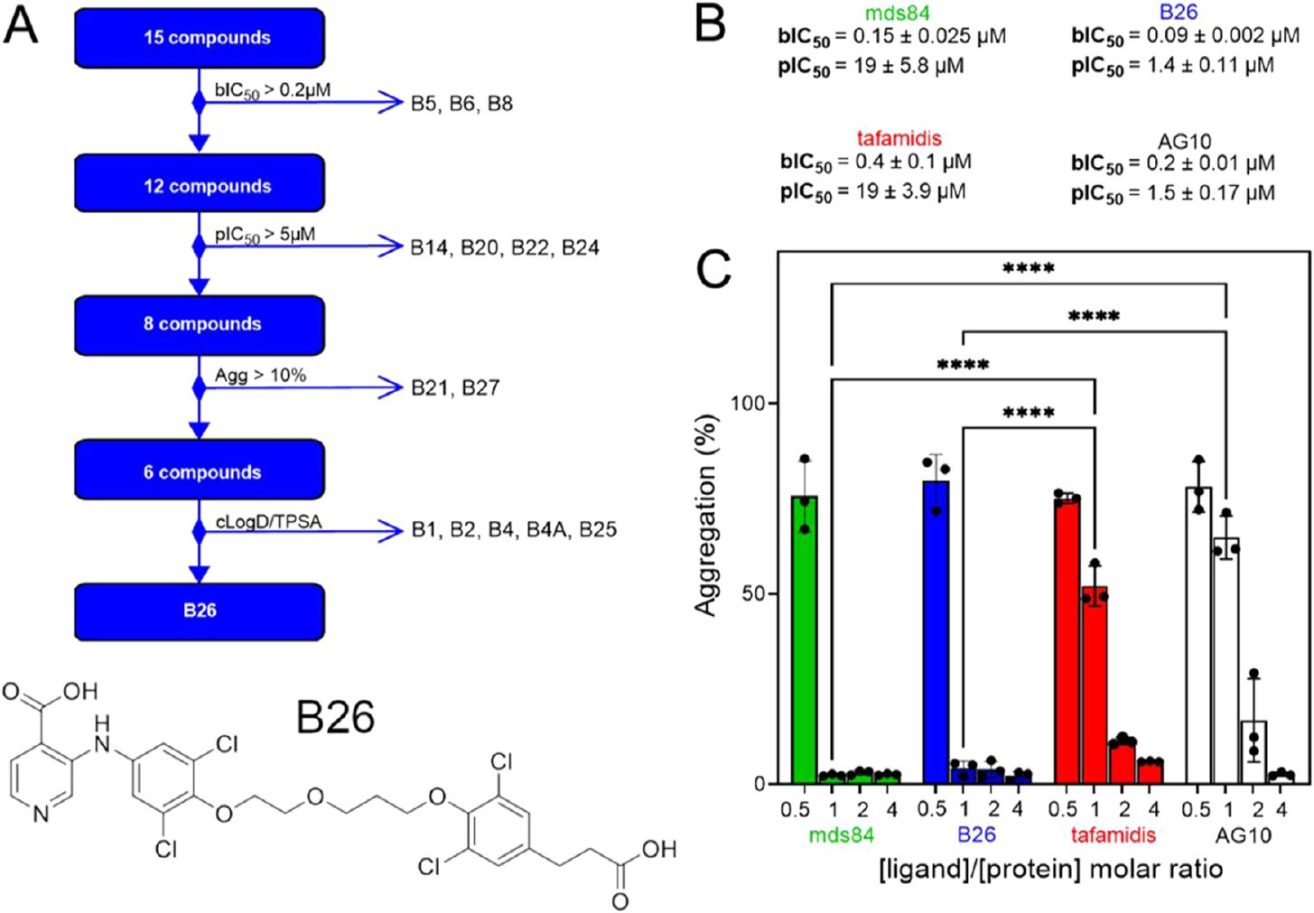
Prototype selection. (A) Flow chart showing the exclusion criteria (bIC_50_ > 0.2 μM, pIC_50_ > 5 μM, Agg >10%, higher cLogD and TPSA values) adopted at each step to choose B26 as a prototype of the bivalent family of TTR ligands. (B) Values of IC_50_ were determined to displace ^125^I-T4 from isolated recombinant wild-type TTR in buffer (bIC_50_) and from wild-type TTR in human plasma (pIC_50_) for mds84, B26, tafamidis, and AG10. (C) Comparative effect of mds84, B26, and monovalent ligands, tafamidis and AG10, on mechano-enzymatic V122I fibrillogenesis. Aggregation of 9 μM V122I TTR in the presence of 0.5-, 1-, 2-, and 4-fold molar excess of B26 or tafamidis or AG10, for each compound quantified as ThT emission fluorescence normalized to the ThT signal of the protein without ligand. All data represent the mean (SD) of three independent experiments. A two-way ANOVA test for pairwise comparison shown to highlight significant differences between the inhibitory effect of B26 (or mds84) vs tafamidis or AG10 at equimolar ligand/protein concentration, with *p* < 0.0001 (****).

### NMR Spectroscopy of the Wild-Type TTR-B26 Complex

The X-ray structures of wild-type TTR and of most TTR variants are all very similar,^27^ in particular, the all-atom root-mean-square deviation (RMSD) between wild-type TTR and V122I is 0.801 Å (Figure S1). This very close structural similarity, also verified by NMR,^28^ validates the use of wild-type TTR to test the binding and allows comparison between the NMR analysis of the complex of wild-type TTR with bound B26 and our previous NMR observations of the complexes of TTR with tafamidis and mds84, respectively.^23^ Superimposition of free (apo) and bound (holo) 2D [^1^H, ^15^N] TROSY NMR spectra of U-[^2^H, ^13^C, ^15^N] wild-type TTR showed that binding of B26 modified about a third of the peaks (Figure 4A). As the ratio of B26:TTR approached equimolar, these changes included the reduction to zero of peaks derived from apo-TTR and the appearance of new peaks of increasing intensity. No further changes were observed at higher B26:TTR molar ratios. This typical slow exchange regime behavior indicates that the apo and holo forms of TTR are observed simultaneously and that the exchange rate between holo and apo (*k*_ex_ = *k*_on_ + *k*_off_) is much lower than the NMR frequency difference between the two forms (Δω). In addition, the complete abrogation of the apo-TTR peaks at 1:1 molar B26:TTR ratio indicates that the ligand has crossed and thus occupied the central channel, thereby occupying both the halogen binding pockets (HBPs) (Figure 1). If the ligand was bound only at a single site, some apo-TTR peaks would be observed or would contribute to the formation of a unique cross-linked polymeric profile.

**Figure 4:**
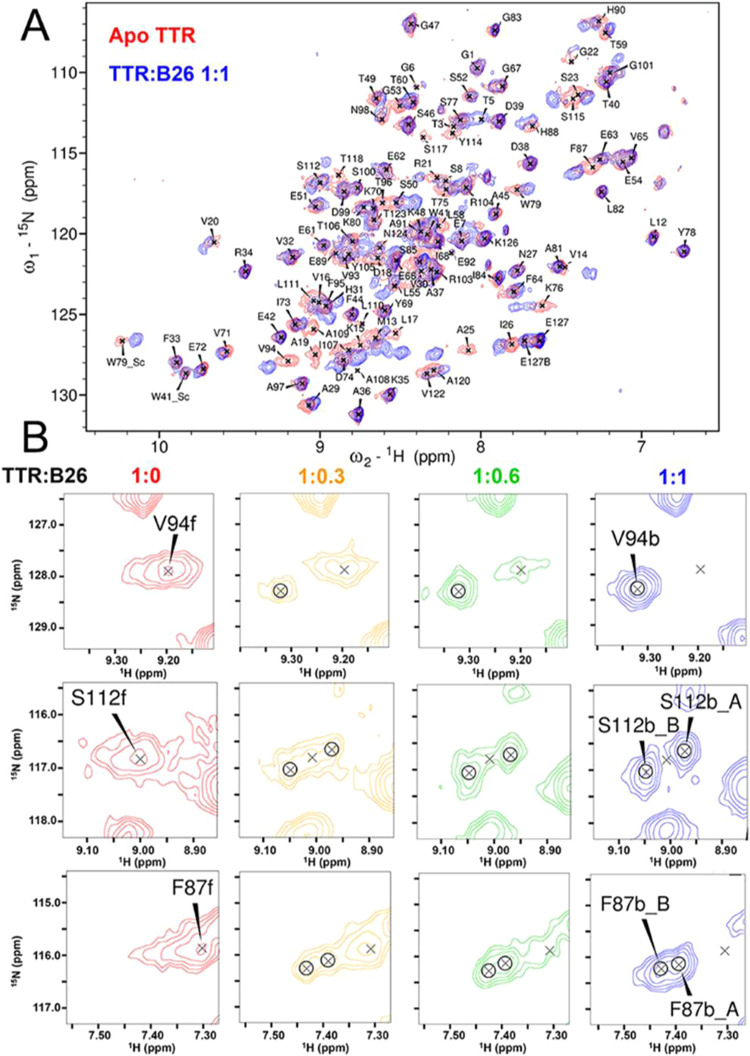
NMR spectra show the binding of B26 to wild-type TTR. (A) Overlay of 2D NMR TROSY spectra of (U–^2^H,^13^C,^15^N)-labeled apo-TTR (red) and after equimolar addition of B26 (blue), recorded at 700 MHz and 37 °C. The assignment of apo-TTR is indicated. The assignment of the B26-bound form was confirmed by 3D HNCA experiments. (B) Selected regions at increasing concentration of B26 (0, 0.3, 0.6, and 1 TTR equivalents) illustrate the slow exchange regime of binding with the disappearance of peaks of the free (f) TTR and the simultaneous appearance of the bound peaks (b). The second and third rows show the growth of the double forms (A, B) for bound peaks.

The asymmetric structure of the heterobivalent ligand, B26, with the headgroup of mds84 at one end and the 3-(3,5-dichlorophenyl) propanoic acid headgroup on the other end enabled observation of residues with perturbed chemical shift located in the HBPs and in the central intramolecular channel connecting them, as well as in regions distant from the HBPs that do not directly contact the ligand. In addition, a number of residues in the holo TTR spectra gave rise to double forms (Figure 4B), verified via the procedure of holo TTR backbone assignment. Indeed, superimposition of the spectra of holo-TTR, complexed with either B26 or mds84, helped to classify, by proximity, which double forms were related to headgroup A or B.

With the exception of residue T106, the chemical shift perturbation (CSP) histogram for head groups A and B deviates significantly from the apo-TTR chemical shift for all residues located in the HBPs and the connecting channel, and L110 showed the greatest deviation (Figure 5).

**Figure 5:**
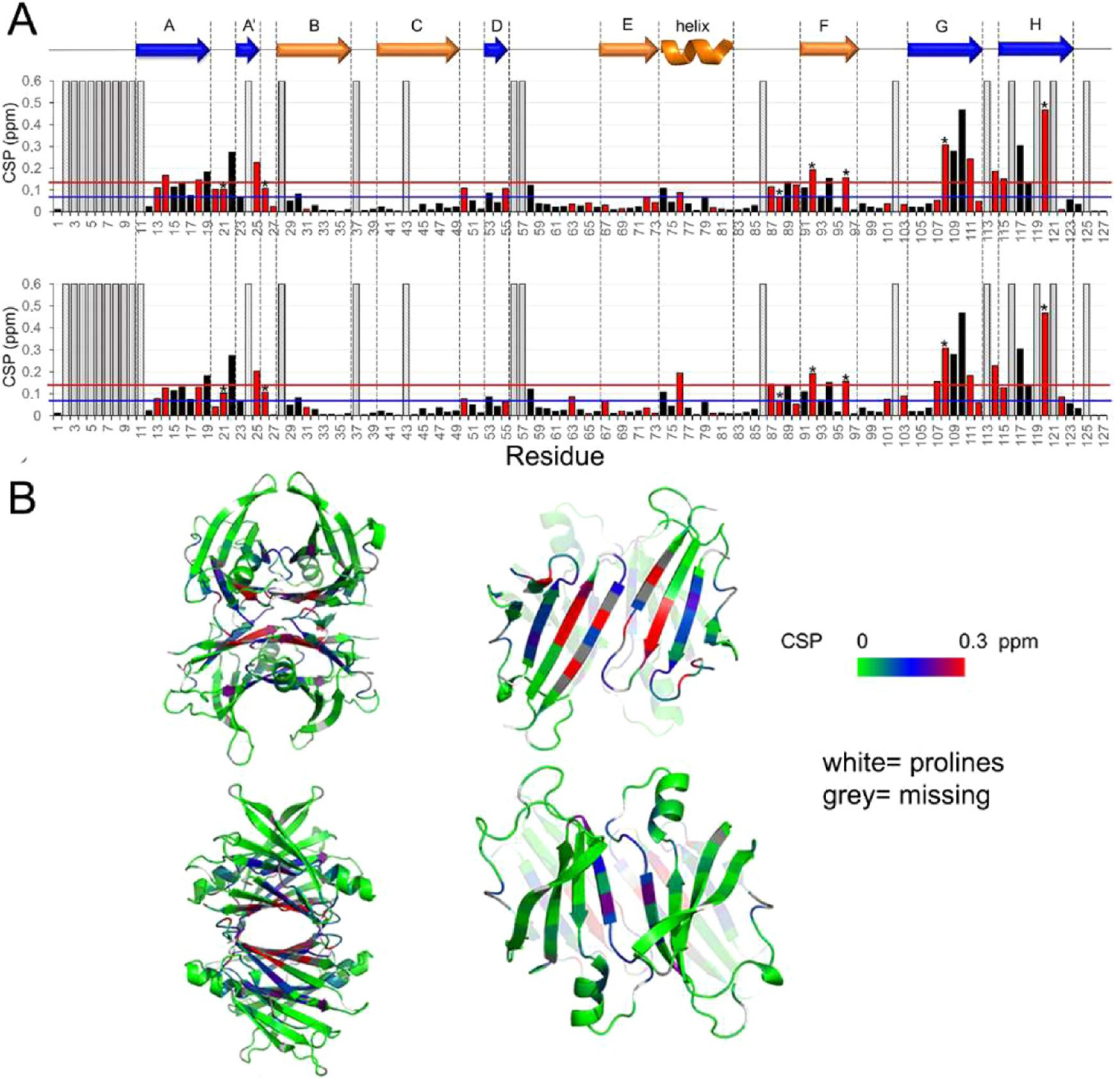
Chemical shift perturbation of wild-type TTR amides upon B26 binding. (A) Combined amide ^1^H and ^15^N chemical shift perturbation at a 1:1 B26:TTR ratio. Amide groups showing two peaks in the bound form (red bars) are reported in the upper plot if assigned to headgroup A or in the lower one if assigned to headgroup B. The double forms that cannot be attributed to a specific headgroup are reported as average CSP values and marked by an asterisk. If no double forms are present, the CSP bar is black. The blue and red lines represent the CSP mean value and the mean value plus one standard deviation, respectively. The filled gray bars refer to unobserved peaks and the striped ones to prolines. The color-coded secondary structure is shown at the top of the histograms: in blue, the β-sheet of the dimer–dimer interface, and in orange, the β-sheet and the helix of the outer region. (B) CSP values are illustrated in a green-blue-red color gradient on the TTR structure (pdb: 5CN3). To better visualize the effects of B26 on the inner A’DAGH β sheet (top right) and on the outer CBEF β sheet together with the helix (bottom right), these elements are shown separately. Cleavage of the bond between residues K48 and T49 in the CD loop is essential for the mechano-enzymatic mechanism of ATTR amyloid fibrillogenesis under physiological conditions *in vivo*. However, these two residues showed limited chemical shift deviation, whereas the flanking residue, S50, was shifted more than average in the holo forms with all ligands. Crucially, loop CD is sandwiched between strand D, where G53 and L55 are slightly perturbed and strand A’ where A25, H-bonded to T49 and I26, are consistently perturbed.

A120, which was extremely weak in 2D TROSY spectra of apo-TTR due to conformational mobility in the μs-ms time scale, is notably immobilized once B26 is bound. The formation of a H-bond between A120 and the facing residue Y114, confirmed by a temperature coefficient higher than the threshold value (−4.5 ppb/K) (see Table S1), contributes to the tetramer stabilization once bound to B26.^23^ Other significant CSPs, outside the binding sites, involve strand F and F’ that are the zippers of the outer cavity. Residue E92 at the N terminal of strand F, which shifted significantly upon binding of B26, is H-bonded to the side chain of Y116 (strand H) located in the channel. In addition, the phenyl ring of Y116 interacts hydrophobically with A91. These three residues clearly illustrate how the perturbation caused by binding of B26 propagates information from the actual binding sites to the outer regions of the protein. Interestingly, peaks representing double forms were of comparable intensity, except K76 in the helix, which showed the most significant difference in CSP between the two head groups. Importantly, one tenth of the peaks were distant more than 12 Å from the ligand rings,^29^ clearly demonstrating that binding of B26 also induces conformational changes in the outer regions of the protein. In particular, strand F, loop H1–F, and helix H1 in the B26 holo form all deviate from apo-TTR.

Electrophoretic analysis of V122I TTR before and after mechano-enzymatic aggregation (Figure S2) showed that B26 inhibited proteolysis, thus confirming the long distant effects of ligand binding that propagate through the channel to the outer surface and stabilize against cleavage and fibrillogenesis.^23^

The NMR analyses thus highlighted not only the ligand binding sites themselves but also, importantly, the overall effects of B26 binding on pathophysiological and potential therapeutic dynamics of the whole TTR protein.

### Docking of B26 with the TTR Structure

The CSPs of 2D [^1^H, ^15^N] TROSY spectra provide information about changes in the chemical environment of each protein amide caused by ligand binding but are not informative about the conformation of the ligand molecule itself. We therefore simulated a docking of TTR with mds84 or B26 in AutoDock4 with the Lamarckian Genetic Algorithm, using a semi-empirical free energy force field^30^ for the evaluation of optimal binding configuration. The docking simulation with mds84 was used to establish the optimal protocol for AutoDock experiments, as the results could be compared with the available X-ray structure (pdb: 3IPE).^22^ The selected best mds84-TTR model overlapped the known crystal structure with RMSD 0.63 Å and calculated binding energy −13.22 kcal/mol. Repeating the same procedure with B26 in 50 starting poses provided two clusters, graded by estimated binding energy. The first cluster contained 20 conformations with an RMSD of 0.32 Å and an average binding energy of −12.75 kcal/mol. The best conformation had an estimated binding energy of −13.08 kcal/mol, very similar to that found in the docking of mds84. Our space-filled model of B26 with the contacting wild-type TTR residues in sticks (Figure 6A) and our 2D representation of hydrophobic and electrostatic interactions in the TTR B26 complex (Figure 6B), are consistent with the binding of B26 mainly involving hydrophobic interactions with HBP residues (Figure S3A). In addition, the formation of an H-bond between the K15 side chain nitrogen (Nζ) and the carboxylic oxygens of headgroup B is suggested, consistent with the X-ray structure of the TTR AG10 complex.^14^ K15, together with K48, R103, and R104, contributes to the formation of a positive electrostatic patch (Figure S3B) at the entrance of the binding channel, which, together with the negative charge of the ligands, makes electrostatics a plausible driving force of the interaction.

**Figure 6:**
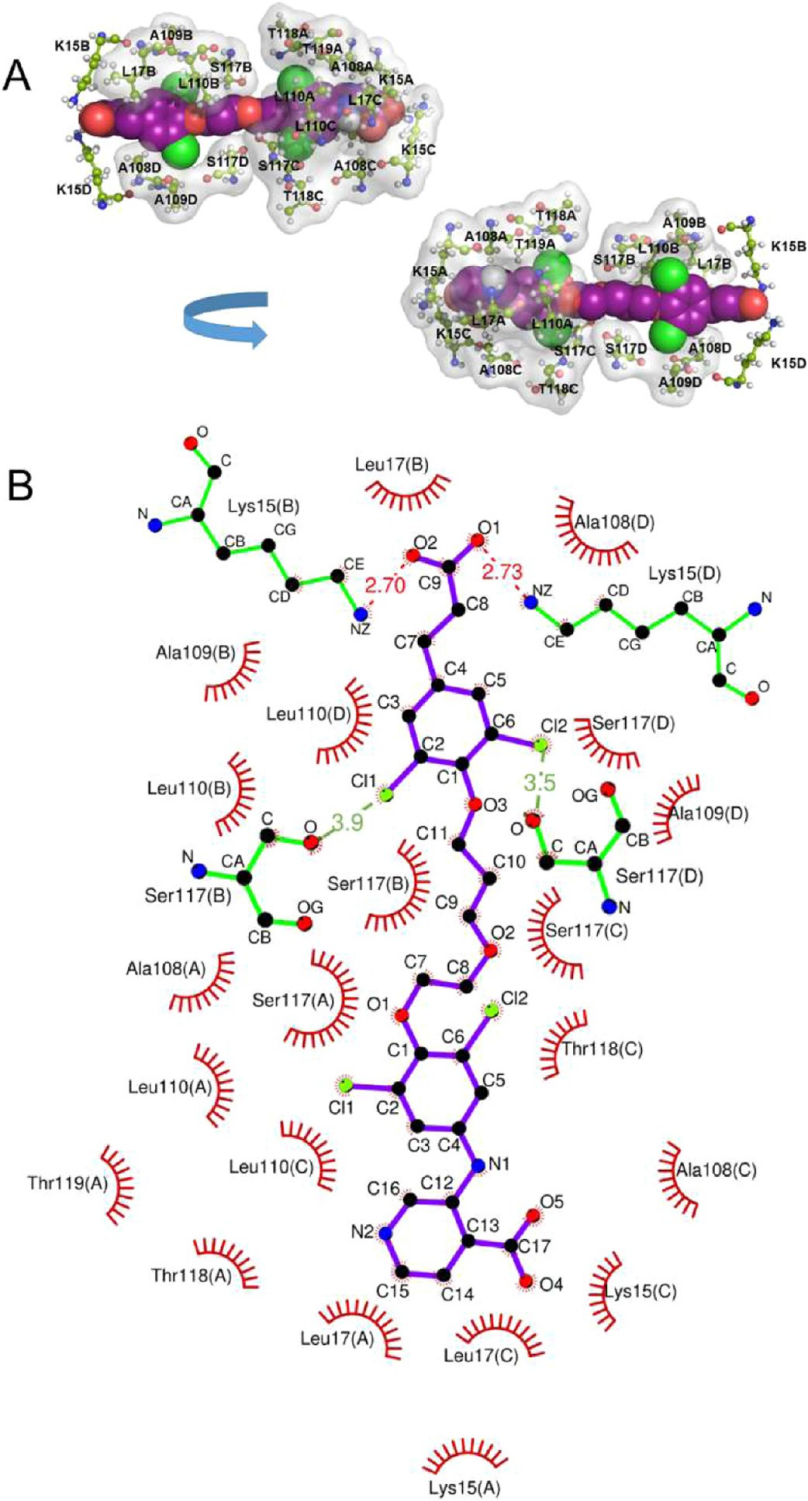
B26 docked to wild-type TTR. (A) Two 180° rotated views of B26 docked to the TTR binding pockets and crossing the central channel show the TTR residues involved in binding. The light gray surface highlights residues involved in hydrophobic interaction with B26. (B) 2D diagram of ligand-protein interaction generated by the Ligplot program for B26 docked to TTR. The ligand (purple) and the protein side chains (green) are shown in the ball-and-stick representation. Hydrogen bonds and halogen bonds are shown as red and green dash-dot-dash lines, while the spoked arcs represent protein residues that make nonbonded contacts with the ligand. The name of the protomer is reported in brackets.

### Stopped Flow Studies of Binding Kinetics

Interaction of ligands in the TTR thyroxine binding pockets induces quenching of intrinsic fluorescence emission.^22^

Analysis of the rapid kinetics of binding within the first second from the rapid mixing of equimolar concentrations of protein and ligands showed that B26 reduced TTR intrinsic fluorescence approximately by 30% with a constant rate of 22 ± 0.55 s^–1^.

Comparative analysis with tafamidis and mds84, under the same experimental conditions, showed that the B26-driven quenching reaction was at least 2 fold slower than tafamidis (*k* = 49 ± 0.0016 s^–1^) and 5 fold faster than mds84 (*k* = 4.4 ± 0.44 s^–1^) (Figure 7).

**Figure 7:**
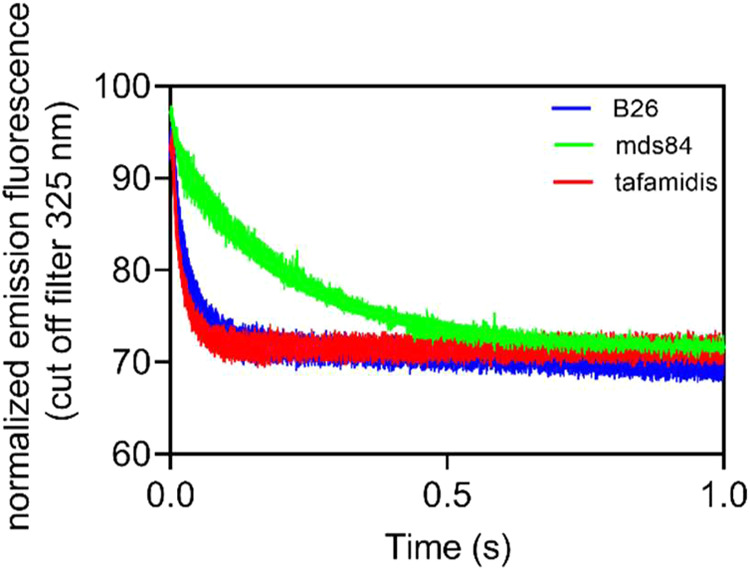
Kinetics of ligand binding by fluorescence emission. Fluorescence emission above 325 nm after excitation at 280 nm of 1 μM native recombinant wild-type TTR with equimolar concentrations of ligands. Data are normalized by attributing 100% fluorescence to the native protein alone. Very early changes in TTR fluorescence occur during the dead time of the measurement (<5 ms) for mds84 (3%) and B26 and tafamidis (approximately 8%).

## Discussion

The possibility of breaking the amyloidogenic pathway of human TTR *in vivo* with specific small ligands represents an extraordinary example of translational medicine from the elucidation of the molecular basis of TTR conversion into fibrils to the design of a drug specifically interfering with the TTR pathological aggregation.^31^

Despite the evidence of the capacity of TTR stabilizers to modify the natural history of the disease,^32^ two major topics are now being addressed: to improve the efficacy of TTR ligands by overcoming the limit of the negative cooperativity of the binding mechanism^33^ and to demonstrate the efficacy of the inhibitors in experimental models that more closely resemble the physiological environment of amyloid formation *in vivo*.

We have already shown that bivalent TTR ligands can be more effective than monovalent counterparts in a model of TTR fibrillogenesis compatible with pathophysiology in a target organ.^21^ However, the prototype mds84 was unsuitable for drug development due to its large and highly hydrophobic structure.^22^ The bivalent compound B26 represents the evolution of such TTR ligands. Compared to the palindromic prototype, the molecule has reduced size and reduced lipophilicity as a consequence of the removal of one of the aryl rings from headgroup B; introduction of an oxygen in the six-atom linker; replacement of the benzoyl group in headgroup A with 4-carboxy pyridine. Despite these structural changes, the ligand retains the same pseudoirreversible binding of mds84.^22^ Indeed, attempts to estimate *k*_off_ using NMR saturation transfer difference (STD) experiments^34^ showed that no STD signal could be observed by increasing the saturation time thus indicating a very slow *k*_off_ in analogy with mds84.^23^ Therefore, the B26 ligand overcomes the pharmaceutical limitations of negative cooperativity and, at the same time, retains the biomolecular property of very strong inhibition of TTR fibrillogenesis by simultaneously occupying the two ligand binding sites. Each of them is able to accommodate two aryl rings, and the one that penetrates deeper into the cavity is anchored to the protein by two halogen substituents. We have designed a bivalent molecule in which one-half has the properties of a classical analogue of the thyroid hormone, and the second half, connected through a chemical linker, is represented by only one aryl ring with halogen substituents.

The new compound has the same potency in inhibiting the mechano-enzymatic mechanism of TTR amyloidogenesis, most likely due to similar long-range structural effects upon binding.^23^ In particular, NMR reveals that B26 fully penetrates TTR through its channel and also provokes conformational changes in the outer regions of the tetramer. Although NMR chemical shifts do not change in the cleavage site (K48 and T49) upon binding of B26, a network of CSPs can be observed in the region that connects Thr49 on one side and the B26 binding site on the other. In particular, A25, which is H-bonded to the side chain of T49 via its O_γ_ is one of the most displaced residues outside the HBP; V16 (bound to the amide of A25) and D18 (H-bonded to V122 and belongs to HBP1) are also more displaced than average (see Movie S1). In addition, B26 induces the formation of an H-bond between A120 (H) and Y114 (H’), which locks the dimer at the edges of the H and H’ opposite strands. Docking simulations also indicate, in addition to a noncovalent bond between the chlorine and the S117 carbonyl common to both B26 and mds84, the formation of a specific H-bond between headgroup B of B26 and K15 side chain, which may be important to increase the binding stability. Overall chemical modifications in B26 have improved the physical-chemical properties in comparison to mds84 without affecting or even enhancing its antiamyloidogenic properties and potency. Changes in the original palindromic compound have actually accelerated the binding kinetics as the penetrance of B26 throughout TTR is 5-fold faster than mds84. Several factors, such as its reduced size and lipophilicity, may have contributed to increasing the flexibility of the molecule that enters into the water-rich TTR channel with a faster binding interaction with TTR compared to the palindromic compound. Further development of B26 could lead to a second generation of inhibitors of TTR amyloidogenesis that may widen the current options for prevention and cure of TTR amyloidosis.

## Experimental Section

### Ligands Synthesis

Syntheses of bivalent ligands are included in the Patent Application no. WO2022/058733 “Agents for use in the treatment of amyloidosis”, 2021 and performed by WuXi AppTech (Shanghai 200131, China). Schemes and experimental procedures for key intermediates (***k1–k8***) and bivalent ligands are here included based on Wuxi reports. All commercially available starting materials and solvents are reagent grade and used without further purification. All ligands are >95% pure (see LCMS and ^1^H NMR spectra in Supporting Information).

### Methyl 3-(3,5-Dichloro-4-hydroxy-phenyl)­Propanoate (Compound ***k1***, Scheme 1)

To a solution of methyl 3-(4-hydroxyphenyl)­propanoate (10 g, 55.49 mmol, 1 equiv) and DIPA (561.54 mg, 5.55 mmol, 784.28 μL, 0.1 equiv) in toluene (1 L) was added sulfuryl chloride (18.72 g, 138.73 mmol, 13.87 mL, 2.5 equiv) at 70 °C. The reaction was stirred at 70 °C for 1 h. The reaction was washed with H_2_O (100 mL*4), brine (100 mL*2), and dried over Na_2_SO_4_, filtered and concentrated to give the residue, then purified by silica gel flash column chromatography (petroleum ether, PE/EtOAc = 100:0–75:25) to give compound ***k1*** (9.5 g, 38.14 mmol, 68.73% yield) as a white solid. ^1^H NMR: (400 MHz, Chloroform-d) δ 7.17–7.05 (m, 2H), 5.84 (s, 1H), 3.73–3.60 (m, 3H), 2.91–2.80 (m, 2H), 2.67–2.52 (m, 2H).

### Methyl 3-[3,5-Dichloro-4-[3-(2-hydroxyethoxy)-propoxy]-phenyl]-Propanoate (Compound ***k2***, Scheme 1)

Step 1: NaH (8.79 g, 219.76 mmol, 60% purity, 1.1 equiv) was added to ethylene glycol (124 g, 2.00 mol, 111.71 mL, 10 equiv) at 0 °C in portions over 2 h. After addition, 1,3-dibromopropane (40.33 g, 199.78 mmol, 20.37 mL, 1 equiv) was added to the above mixture, and it was stirred at 60 °C for 4 h. The reaction mixture was diluted with H_2_O (60 mL) and extracted with EtOAc (50 mL*3). The combined organic layers were washed with brine (70 mL), dried over Na_2_SO_4_, filtered, and concentrated under reduced pressure to give a residue. The residue was purified by silica gel flash column chromatography (PE/EtOAc = 10:1–2:1) to afford 2-(3-bromopropoxy)-ethanol (8.6 g, 46.98 mmol, 23.52% yield) as a colorless oil. ^1^H NMR: (Methanol-d4, 400 MHz); δ 6.01–5.86 (m, 1H), 5.36–5.11 (m, 1H), 4.02 (d, *J* = 5.6 Hz, 1H), 3.72–3.64 (m, 2H), 3.60 (t, *J* = 5.9 Hz, 1H), 3.57–3.50 (m, 3H), 2.10 (quin, *J* = 6.2 Hz, 1H).

Step 2: To a solution of ***k1*** (1.5 g, 6.02 mmol, 1 equiv) and 2-(3-bromopropoxy)-ethanol (1.32 g, 7.23 mmol, 1.2 equiv) in DMF (120 mL) was added K_2_CO_3_ (2.50 g, 18.07 mmol, 3 equiv) and KI (999.64 mg, 6.02 mmol, 1 equiv). The mixture was stirred at 60 °C for 2 h. The reaction mixture was diluted with H_2_O (100 mL) and extracted with EtOAc (80 mL*3). The combined organic layers were washed with brine (100 mL), dried over Na_2_SO_4_, filtered and concentrated under reduced pressure to give a residue then purified by silica gel flash column chromatography (PE/EtOAc = 10:1–2:1) to afford compound ***k2*** (1.4 g, 3.99 mmol, 66.19% yield) as yellow oil. ^1^H NMR: (Methanol-d4, 400 MHz); δ 7.23 (s,2H), 4.07 (m, 2H), 3.73 (t, *J* = 6.3 Hz, 2H), 3.70–3.65 (m, 2H), 3.64 (s, 3H), 3.57–3.53 (m, 2H), 2.86–2.82 (m, 2H), 2.65–2.60 (m, 2H), 2.08 (quin, *J* = 6.3 Hz, 2H).

For the synthesis of B24 and B25, the compound ***k2*** in DCM was further converted into the corresponding p-tosylsulphonyl derivative by adding TEA and Tos-Cl at 0 °C. After stirring at 20 °C for 12 h, the reaction was concentrated and the residue was further purified by flash silica gel chromatography to give methyl (E)-3-[3,5-dichloro-4-[3-[2-(p-tolylsulfonyloxy)-ethoxy]-propoxy]-phenyl]-prop-2-enoate (***Tos*** **-** ***k2*** **)**.

### Methyl 2-(3,5-Dichloro-4-hydroxy-anilino)-Benzoate (Compound ***k3***, Scheme 1)

Step 1: To a mixture of 2,6-dichloro-4-nitro phenol (50 g, 240.39 mmol, 1 equiv) and Cs_2_CO_3_ (195.81 g, 600.97 mmol, 2.5 equiv) in NMP (800 mL) was added bromomethylbenzene (61.67 g, 360.58 mmol, 42.83 mL, 1.5 equiv) dropwise at 20 °C, the reaction mixture was stirred at 20 °C for 12 h. The reaction mixture was filtered and diluted with EtOAc (1200 mL), washed with subsaturated brine (600 mL*3).

The organic layer was dried over Na_2_SO_4_ and concentrated to give a residue, then purified by silica gel flash column chromatography (PE/EtOAc = 100:1–100:4) and washed with MeOH (50 mL) to afford 2-benzyloxy-1,3-dichloro-5-nitro-benzene (30 g) as a white solid. ^1^H NMR: (Chloroform-d, 400 MHz); δ 8.24 (s, 2H), 7.54 (dd, *J* = 1.7, 7.5 Hz, 2H), 7.46–7.36 (m, 3H), 5.18 (s, 2H).

Step 2: To a solution of 2-benzyloxy-1,3-dichloro-5-nitro benzene (30 g, 100.63 mmol, 1 equiv) in EtOH (600 mL) and H_2_O (120 mL) was added Fe (28.10 g, 503.15 mmol, 5 equiv) and NH_4_Cl (26.91 g, 503.15 mmol, 5 equiv) at 20 °C in portions, the reaction mixture was stirred at 60 °C for 6 h. The reaction mixture was filtered and concentrated to give a residue, then purified by silica gel flash column chromatography (PE/EtOAc = 5:1–1:1) to afford 4 benzyloxy-3,5-dichloro-aniline (25 g, 93.24 mmol, 92.65% yield) as a white solid. ^1^H NMR: (Chloroform-d, 400 MHz); δ 7.57 (d, *J* = 6.8 Hz, 2H), 7.46–7.30 (m, 3H), 6.64 (s, 2H), 4.96 (s, 2H), 3.64 (br s, 2H).

Step 3: To a mixture of 4 benzyloxy-3,5-dichloro-aniline (25 g, 93.24 mmol, 1 equiv), methyl 2-bromobenzoate (30.07 g, 139.85 mmol, 19.66 mL, 1.5 equiv) and Cs_2_CO_3_ (75.94 g, 233.09 mmol, 2.5 equiv) in toluene (300 mL) was added BINAP (4.35 g, 6.99 mmol, 0.075 equiv) and Pd_2_(dba)­3 (4.27 g, 4.66 mmol, 0.05 equiv) at 25 °C, the reaction mixture was degassed and purged with N_2_ for 3 times and then stirred at 120 °C for 12 h. The reaction mixture was filtered and the filter cake was washed with MeOH (300 mL) and hexane (200 mL) to give a residue then purified by silica gel flash column chromatography (PE/EtOAc = 20:1) to afford methyl 2-(4-benzyloxy-3,5-dichloro-anilino)-benzoate (32 g, 79.55 mmol, 85.32% yield) as a light-yellow solid. ^1^H NMR: (Chloroform-d, 400 MHz); δ 9.50 (s, 1H), 8.04 (dd, *J* = 1.4, 8.0 Hz, 1H), 7.63 (d, *J* = 7.0 Hz, 2H), 7.51–7.39 (m,4H), 7.31 (d, *J* = 6.2 Hz, 1H), 6.88 (t, *J* = 7.5 Hz, 1H), 5.09 (s, 2H), 3.96 (s, 3H).

Step 4: To a solution of methyl 2-(4-benzyloxy-3,5-dichloro-anilino)-benzoate (15 g, 37.29 mmol, 1 equiv) in MeOH (300 mL) and THF (300 mL) was added Pd/C (4 g, 10% purity). The reaction mixture was degassed and purged with H_2_ for 3 times, stirred under H_2_ (15 psi) at 25 °C for 2 h, and then filtered. The filtrate was concentrated to give a residue, then purified by silica gel flash column chromatography (PE/EtOAc = 35:1) to afford compound ***k3*** (99.4% purity) (22 g) as a light yellow solid. ^1^H NMR: (Chloroform-d, 400 MHz); δ 9.32 (br s, 1H), 7.97 (dd, *J* = 1.4, 8.0 Hz, 1H), 7.39–7.31 (m, 1H), 7.20 (s, 2H), 7.05 (d, *J* = 8.4 Hz, 1H), 6.77 (t, *J* = 7.5 Hz, 1H), 5.72 (s, 1H), 3.91 (s, 3H).

### Methyl 3-[3,5-Dichloro-4-[(E)-6-hydroxyhex-3-enoxy]-phenyl] Propanoate (Compound ***k4***, Scheme 1)

Step 1: Acetyl chloride (26.40 g, 336.32 mmol, 24 mL, 4.04 equiv) was slowly added to *i*-PrOH (180 mL) at −5 °C. After being stirred at −5 °C for 10 min, (E)-hex-3-enedioic acid (12 g, 83.26 mmol, 1 equiv) was added, and the reaction was heated to 80 °C and stirred for 12 h. The reaction was concentrated to give a residue, then diluted with EtOAc (500 mL) and washed with aq. NaHCO_3_ (100 mL*4). The organic layer was washed with brine (200 mL), dried over Na_2_SO_4_, filtered and concentrated under vacuum to give a residue purified by silica gel flash column chromatography (PE/EtOAc = 100:1–50:1) to afford diisopropyl (E)-hex-3-enedioate (28 g, 122.65 mmol, 73.66% yield) as a yellow solid. ^1^H NMR: (Chloroform-d, 400 MHz); δ 5.68 (tt, *J* = 1.7, 3.7 Hz, 2H), 5.00 (spt, *J* = 6.3 Hz, 2H), 3.07–3.03 (m, 4H), 1.23 (d, *J* = 6.4 Hz, 12H).

Step 2: To a suspension of Lithium Aluminum Hydride (LAH, 16.63 g, 438.05 mmol, 4 equiv) in THF (100 mL) was added diisopropyl (E)-hex-3-enedioate (25 g, 109.51 mmol, 1 equiv) in THF (100 mL) dropwise and the reaction mixture stirred at 15 °C for 12 h. The reaction mixture was quenched with H_2_O (16.7 mL) at 0 °C dropwise before adding NaOH (aq, 15% w/w, 16.7 mL) followed by H_2_O (50 mL) at 0 °C. The suspension was filtered, and the filtrate was concentrated to afford (E)-hex-3-ene-1,6-diol (12 g, 103.31 mmol, 94.33% yield) as a light yellow oil. ^1^H NMR: (Chloroform-d, 400 MHz); δ 5.55–5.47 (m, 2H), 3.62 (q, *J* = 5.1 Hz, 4H), 2.85–2.72 (m, 2H), 2.37–2.19 (m,4H).

Step 3: To a solution of (E)-hex-3-ene-1,6-diol (12 g, 103.31 mmol, 2.57 equiv), ***k1*** (10 g, 40.15 mmol, 1 equiv) and PPh_3_ (21.06 g, 80.29 mmol, 2 equiv) in THF (300 mL) was added a solution of DIAD (16.24 g,80.29 mmol, 15.61 mL, 2 equiv) in THF (50 mL) at 0 °C. The reaction mixture was stirred at 15 °C for 12 h and then concentrated to give the residue, further purified by silica gel flash column chromatography (PE/EtOAc = 50:1–5:1) to afford compound ***k4*** (20 g, 83% LCMS purity) as a light yellow oil. LCMS: (M+H^+^): 347.1 @ 1.252 min (5–95% ACN in H_2_O, 2.0 min).

### Methyl 2-[3,5-Dichloro-4-[(E)-6-[2,6-dichloro-4-(3-methoxy-3-oxo-propyl)-phenoxy]-hex-3-enoxy]-anilino]-Benzoate (Compound ***k5***, Scheme 2)

To a mixture of compound ***k4***, (6 g, 17.28 mmol, 1.2 equiv), ***k3*** (4.49 g, 14.40 mmol, 1 equiv) and PPh_3_ (7.55 g, 28.80 mmol, 2 equiv) in THF (25 mL) was added DIAD (5.82 g, 28.80 mmol, 5.60 mL, 2 equiv) at 0 °C. After stirring at 15 °C for 12 h, the reaction was concentrated to give the residue, which was purified by silica gel flash column chromatography (PE/EtOAc = 100:0–98:2) to afford compound ***k5*** (4.6 g, 6.67 mmol, 46.32% yield, 93% purity) as a yellow solid. ^1^H NMR: (Chloroform-d, 400 MHz); δ 9.41 (s, 1H), 7.98 (dd, *J* = 1.6, 8.1 Hz, 1H), 7.38 (ddd, *J* = 1.6, 7.2, 8.5 Hz, 1H), 7.23–7.17 (m, 3H), 7.13 (s, 2H), 6.81 (ddd, *J* = 1.0, 7.2, 8.1 Hz, 1H), 5.79–5.70 (m, 2H), 4.04 (dt, *J* = 3.2, 6.9 Hz, 4H), 3.91 (s, 3H),3.69 (s, 3H), 2.90–2.80 (m, 2H), 2.61 (br t, *J* = 7.6 Hz, 6H).

### Methyl 2-(3-Chloro-4-hydroxy-anilino)-Benzoate (Compound ***k6***, Scheme 2)

Step 1: To a mixture of 2-chloro-4-nitro-phenol (25 g, 144.05 mmol, 1 equiv) and Cs_2_CO_3_ (117.34 g, 360.13 mmol, 2.5 equiv) in NMP (400 mL) was added bromomethylbenzene (24.64 g, 144.05 mmol, 17.11 mL, 1 equiv) dropwise at 15 °C, and the reaction mixture was stirred at 15 °C for 12 h before dilution with H_2_O (1000 mL) and extraction with EtOAc (500 mL*3). The organic layer was washed with brine (200 mL) and concentrated to give the residue. The crude product was purified by recrystallization from MTBE (100 mL) at 15 °C to give 1-benzyloxy-2-chloro-4-nitro-benzene (31 g, 117.57 mmol, 81.62% yield) as a yellow solid. ^1^H NMR: (Chloroform-d, 400 MHz); δ 8.31 (d, *J* = 2.8 Hz, 1H), 8.23 (dd, *J* = 2.8, 9.2 Hz, 1H), 7.57–7.27 (m, 6H), 5.37 (s, 2H).

Step 2: To a solution of 1-benzyloxy-2-chloro-4-nitro-benzene (31 g, 117.57 mmol, 1 equiv) and NH_4_Cl (31.44 g, 587.84 mmol, 5 equiv) in EtOH (150 mL) and H_2_O (150 mL) was added Fe (32.83 g, 587.84 mmol, 5 equiv) in portions. The mixture was stirred at 60 °C for 12 h. The suspension was filtered through a pad of Celite, and the filter cake was washed with EtOAc (1.5 L). The combined filtrates were concentrated to dryness to give crude product, then purified by silica gel flash column chromatography (PE/EtOAc = 100:0–70:30) to afford 4-benzyloxy-3-chloro-aniline (25.4 g, 108.69 mmol, 92.45% yield) as a brown solid. ^1^H NMR: (Chloroform-d, 400 MHz); δ 7.49–7.43 (m, 2H), 7.42–7.35 (m, 2H), 7.35–7.29 (m, 1H), 6.80 (d, *J* = 8.7 Hz, 1H), 6.76 (d, *J* = 2.7 Hz, 1H), 6.51 (dd, *J* = 2.8, 8.6 Hz, 1H), 5.06 (s, 2H), 3.49 (br s, 2H).

Step 3: A mixture of 4-benzyloxy-3-chloro-aniline (5 g, 21.40 mmol, 1 equiv), methyl 2-bromobenzoate (4.60 g, 21.40 mmol, 3.01 mL, 1 equiv), BINAP (999.18 mg, 1.60 mmol, 0.075 equiv), Pd2­(dba)­3 (979.62 mg, 1.07 mmol, 0.05 equiv), and Cs_2_CO_3_ (17.43 g, 53.49 mmol, 2.5 equiv) in toluene (70 mL) was degassed and purged with N_2_ for 3 times, and then the mixture was stirred at 130 °C for 12 h under N_2_ atmosphere. The reaction mixture was diluted with H_2_O (200 mL) and partitioned; the aqueous layer was extracted with EtOAc (100 mL*2). The combined organic layers were washed with brine (200 mL), dried over Na_2_SO_4_, filtered and concentrated under reduced pressure to give a residue then purified by silica gel flash column chromatography (PE/EtOAc = 100:0–98:2) to afford methyl 2-(4-benzyloxy-3-chloro-anilino)-benzoate (6.5 g, 17.67 mmol, 82.59% yield) as a white solid. ^1^H NMR: (Chloroform-d, 400 MHz); δ 9.31 (s, 1H), 7.96 (dd, *J* = 1.5, 8.0 Hz, 1H), 7.52–7.46 (m, 2H), 7.41 (t, *J* = 7.4 Hz, 2H), 7.37–7.28 (m, 3H), 7.09–7.03 (m, 2H), 6.96 (d, *J* = 8.7 Hz, 1H), 6.75–6.69 (m, 1H), 5.17 (s, 2H), 3.91 (s, 3H).

Step 4: To a solution of methyl 2-(4-benzyloxy-3-chloro-anilino)-benzoate (6 g, 16.31 mmol, 1 equiv) in MeOH (60 mL) and THF (60 mL) was added Pd/C (1.5 g, 16.31 mmol, 10% purity) under Ar. The suspension was degassed under vacuum and purged with H_2_ several times. The mixture was stirred under H_2_ (15 psi) at 15 °C for 2 h. The suspension (500 mg) was filtered through a pad of Celite, and the filter cake was washed with EtOAc (1000 mL). The combined filtrates were concentrated to give a crude product, then purified by silica gel flash column chromatography (PE/EtOAc = 100:0–97:3) to yield compound ***k6*** (4.3 g) as a yellow solid. ^1^H NMR: (Chloroform-d, 400 MHz); δ 9.29 (br s, 1H), 7.96 (dd, *J* = 1.4, 8.0 Hz, 1H), 7.34–7.28 (m, 1H), 7.25 (d, *J* = 2.4 Hz, 1H), 7.11–7.05 (m, 1H), 7.04–6.96 (m, 2H), 6.75–6.70 (m, 1H), 5.45 (s, 1H), 3.95–3.88 (m, 3H).

### Methyl 3-[3-Chloro-4-[(E)-6-hydroxyhex-3-enoxy]-phenyl]-Propanoate (Compound ***k7***, Scheme 2)

Step 1: To a solution of methyl 3-(4-hydroxyphenyl)-propanoate (10 g, 55.49 mmol, 1 equiv) in CCl_4_ (100 mL) was added sulfuryl chloride (8.24 g, 61.04 mmol, 6.10 mL, 1.1 equiv). The mixture was stirred at 70 °C for 3 h. The reaction was quenched by the addition of H_2_O (150 mL) and partitioned, and the aqueous layer was extracted with EtOAc (80 mL*2). The combined organic layers were washed with brine (150 mL), dried over Na_2_SO_4_, filtered and concentrated under reduced pressure to give a residue then purified by silica gel flash column chromatography (PE/EtOAc = 100:0–93:7) to give a crude product (12 g) then purified by reversed-phase MPLC (HCl condition) to afford methyl 3-(3-chloro-4-hydroxy-phenyl)-propanoate (9 g, 37.74 mmol, 68.00% yield, 90% purity) as a yellow solid. ^1^H NMR: (Chloroform-d, 400 MHz); δ 7.16 (d, *J* = 1.8 Hz, 1H), 7.03–6.97 (m, 1H), 6.96–6.90 (m, 1H), 5.56 (br s, 1H), 3.68 (s, 3H), 2.91–2.83 (m, 2H), 2.63–2.56 (m, 2H).

Step 2: To a solution of (E)-hex-3-ene-1,6-diol (5.41 g, 46.58 mmol, 2 equiv), methyl 3-(3-chloro-4-hydroxy-phenyl)-propanoate (5 g, 23.29 mmol, 1 equiv) and PPh_3_ (12.22 g, 46.58 mmol, 2 equiv) in THF (100 mL) was added DIAD (9.42 g, 46.58 mmol, 9.06 mL, 2 equiv). The mixture was stirred at 15 °C for 12 h and then concentrated under reduced pressure to remove solvent. The residue was purified by silica gel flash column chromatography (PE/EtOAc = 100:0–84:16) to give a crude product (12 g) then purified by reversed MPLC (HCl condition) to afford compound ***k7*** (3 g, 9.59 mmol, 41.18% yield) as yellow oil. ^1^H NMR: (Chloroform-d, 400 MHz); δ 7.20 (d, *J* = 2.0 Hz, 1H), 7.02 (dd, *J* = 2.1, 8.3 Hz, 1H), 6.83 (d, *J* = 8.4 Hz, 1H), 5.70–5.53 (m, 2H), 4.03 (t, *J* = 6.5 Hz, 2H), 3.69–3.63 (m, 5H), 2.90–2.83 (m, 2H), 2.63–2.52 (m, 4H), 2.30 (q, *J* = 6.4 Hz, 2H).

### Methyl-3-[4-[3-[2-(4-Amino-2,6-dichloro-phenoxy)-ethoxy]-propoxy]-3,5-dichloro-phenyl]-Propanoate (Compound ***k8***, Scheme 2)

Step 1: To a solution of compound ***k2*** (2.5 g, 7.12 mmol, 1 equiv), 2,6-dichloro-4-nitro-phenol (1.78 g, 8.54 mmol, 1.2 equiv), PPh_3_ (2.80 g, 10.68 mmol, 1.5 equiv) in THF (20 mL) was added dropwise DIAD (2.16 g, 10.68 mmol, 2.08 mL, 1.5 equiv) at 0 °C. The reaction was stirred at 20 °C for 12 h and then concentrated to give the residue. The crude product was purified by reversed-phase HPLC (0.1% HCl condition, MeOH/H_2_O) to give methyl 3-[3,5-dichloro-4-[3-[2-(2,6-dichloro-4-nitro-phenoxy)-ethoxy]-propoxy]-phenyl]-propanoate (1.5 g, 2.77 mmol, 38.94% yield) as a brown oil.

Step 2: To a solution of methyl 3-[3,5-dichloro-4-[3-[2-(2,6-dichloro-4-nitro-phenoxy)-ethoxy]-propoxy]-phenyl]-propanoate (1.5 g, 2.77 mmol, 1 equiv) in EtOH (50 mL)/H_2_O (10 mL) was added NH_4_Cl (148.26 mg, 2.77 mmol, 1 equiv). Fe (773.90 mg, 13.86 mmol, 5 equiv) was added in batches. After stirring at 60 °C for 2 h, the reaction mixture was filtered through the Celite, washed with MeOH (50 mL) and, concentrated to give the residue then purified by silica gel flash column chromatography (PE/EtOAc = 100:0–90:10) to give compound ***k8*** (1.4 g, 2.74 mmol, 98.81% yield) as a brown oil. ^1^H NMR: (400 MHz, Chloroform-d); δ 7.12 (s, 2H), 6.63–6.52 (m, 2H), 4.10 (br d, *J* = 3.4 Hz, 2H), 3.88–3.76 (m, 4H),3.73–3.59 (m, 5H), 2.85 (t, *J* = 7.6 Hz, 2H), 2.65–2.55 (m, 2H), 2.13 (quin, *J* = 6.3 Hz, 2H).

### Synthesis of 2-[4-[2-[3-[4-(2-Carboxyethyl)-2,6-dichloro-phenoxy]-propoxy]-ethoxy]-3,5-dichloro-anilino]-Benzoic Acid (B1) (Scheme 3)

Step 1: to a solution of compound ***k2*** (1.4 g, 3.99 mmol, 1 equiv) and ***k3*** (1.24 g, 3.99 mmol, 1 equiv) in THF (15 mL) was added PPh_3_ (1.57 g, 5.98 mmol, 1.5 equiv) and DIAD (1.21 g, 5.98 mmol, 1.16 mL, 1.5 equiv). The mixture was stirred at 20 °C for 12 h, and then further concentrated under reduced pressure to remove solvent. The residue was diluted with H_2_O (40 mL) and extracted with EtOAc (25 mL*3). The combined organic layers were washed with brine (30 mL), dried over Na_2_SO_4_, filtered and concentrated under reduced pressure to give a residue then purified by silica gel flash column chromatography (PE/EtOAc = 100:0–90:10) to afford methyl 2-[3,5-dichloro-4-[2-[3-[2,6-dichloro-4-(3-methoxy-3-oxo-propyl)-phenoxy]-propoxy]-ethoxy]-anilino]-benzoate (780 mg, 90% purity) as yellow oil. ^1^H NMR: (Chloroform-d, 400 MHz); δ 9.42 (s, 1H), 7.98 (dd, *J* = 1.5, 8.1 Hz, 1H), 7.38 (ddd, *J* = 1.5, 7.2, 8.5 Hz, 1H), 7.24–7.20 (m, 1H), 7.18 (s, 2H), 7.12 (s, 2H), 6.81 (ddd, *J* = 1.1, 7.1, 8.0 Hz, 1H), 4.22–4.18 (m, 2H), 4.14–4.11 (m, 2H), 3.91 (s, 3H), 3.90–3.86 (m, 2H), 3.83 (t, *J* = 6.3 Hz, 2H), 3.68 (s, 3H), 2.89–2.82 (m, 2H), 2.63–2.57 (m, 2H), 2.15 (quin, *J* = 6.3 Hz, 2H).

Step 2: To a solution of methyl 2-[3,5-dichloro-4-[2-[3-[2,6-dichloro-4-(3-methoxy-3-oxopropyl)-phenoxy]-propoxy]-ethoxy]-anilino]-benzoate (580 mg, 898.73 μmol, 1 equiv) in THF (28 mL) and MeOH (14 mL) was added LiOH·H_2_O (226.28 mg, 5.39 mmol, 6 equiv) in H_2_O (14 mL). The mixture was stirred at 40 °C for 2 h and concentrated to give the residue, which was then diluted with H_2_O (50 mL) and extracted with DCM (30 mL*2). The organic layer was discarded. The aqueous layer was adjusted with HCl (1N) to pH 2 and extracted with DCM (70 mL*4). The organic layer was washed with brine (30 mL*3), dried over Na_2_SO_4_, filtered, and concentrated to give the residue, then purified by prep-HPLC (HCl condition) to afford B1 (301.9 mg, 99.57% purity) as a white solid. LCMS: (M+H^+^): 618.0 @ 2.193 min (10–80% ACN in H_2_O, 3.0 min). ^1^H NMR: (DMSO-d6, 400 MHz); δ 9.48 (s, 1H), 7.91 (dd, *J* = 1.6, 7.9 Hz, 1H), 7.44 (dt, *J* = 1.6, 7.8 Hz, 1H), 7.33 (d, *J* = 10.0 Hz,4H), 7.23 (d, *J* = 8.1 Hz, 1H), 6.92–6.84 (m, 1H), 4.14–4.07 (m, 2H), 4.02 (t, *J* = 6.5 Hz, 2H), 3.78–3.73 (m, 2H), 3.68 (t, *J* = 6.2 Hz, 2H), 2.79–2.73 (m, 2H), 2.56–2.52 (m, 2H), 2.00 (quin, *J* = 6.3 Hz, 2H).

### Synthesis of 2-[4-[4-[2-[4-(2-Carboxyethyl)-2,6-dichloro-phenoxy]-ethoxy]-butoxy]-3,5-dichloro-anilino]-Benzoic Acid (B2) (Scheme 4)

Step 1: to a mixture of 4-bromobutan-1-ol (5 g, 32.68 mmol, 1 equiv) and [chloro­(diphenyl)­methyl] benzene (13.66 g, 49.01 mmol, 1.5 equiv) in DCM (100 mL) was added TEA (6.61 g, 65.35 mmol, 9.10 mL, 2 equiv) at 0 °C. The reaction mixture was slowly warmed to 15 °C and stirred for 12 h. It was concentrated to give the residue, then purified by silica gel flash column chromatography (PE/EtOAc = 100:0–20:1) to afford [4-bromobutoxy­(diphenyl)­methyl]-benzene (5.7 g, 14.42 mmol, 44.12% yield) as a colorless oil. ^1^H NMR: (Chloroform-d, 400 MHz); δ 7.38–7.31 (m, 5 H), 7.25–7.10 (m, 10H), 3.29 (t, *J* = 6.8 Hz, 2H), 3.01 (t, *J* = 6.2 Hz,2H), 1.96–1.82 (m, 2H), 1.71–1.61 (m, 2H).

Step 2: To a solution of ***k1*** (10 g, 40.15 mmol, 1 equiv) in DMF (100 mL) was added Cs_2_CO_3_ (26.16 g, 80.29 mmol, 2 equiv) at 0 °C. After 5 min, 2-bromoethanol (7.53 g, 60.22 mmol, 4.28 mL, 1.5 equiv) and the mixture was stirred at 80 °C for 12 h. The reaction was diluted with H_2_O (50 mL) and extracted with EtOAc (70 mL*3). The organic layer was washed with brine (20 mL*2), dried over Na_2_SO_4_, filtered and concentrated to give the residue then purified by silica gel flash column chromatography (PE/EtOAc = 10:1–3:1) to afford methyl 3-[3,5-dichloro-4-(2-hydroxyethoxy)-phenyl]-propanoate (3.5 g, 11.94 mmol, 29.74% yield) as a colorless oil. ^1^H NMR: (Chloroform-d, 400 MHz); δ 7.20–7.09 (m,2H), 4.20–4.13 (m, 2H), 3.98–3.90 (m, 2H), 3.75–3.62 (m, 3H), 2.90–2.83 (m, 2H), 2.64–2.56 (m, 2H).

Step 3: To a solution of methyl 3-[3,5-dichloro-4-(2-hydroxyethoxy)-phenyl]-propanoate (2.5 g, 8.53 mmol, 1 equiv) in DMF (30 mL) was added NaH (1.02 g, 25.58 mmol, 60% purity, 3 equiv) at 0 °C. The reaction was stirred at 0 °C for 0.5 h and [4-bromobutoxy­(diphenyl)­methyl]-benzene (4.05 g, 10.23 mmol, 1.2 equiv) was added to the mixture at 0 °C. After stirring at 80 °C for 12 h, the black mixture was quenched with NH_4_Cl aq. (100 mL) and extracted with EtOAc (100 mL*5). The organic was washed with brine (100 mL), dried over Na_2_SO_4_, filtered and concentrated to give a residue then purified by silica gel flash column chromatography (PE/EtOAc = 100:0–20:1) to afford methyl 3-[3,5-dichloro-4-[2-(4-trityloxybutoxy)-ethoxy]-phenyl]-propanoate (1.4 g, 2.30 mmol, 27.02% yield) as a light brown oil.

Step 4: To a solution of methyl 3-[3,5-dichloro-4-[2-(4-trityloxybutoxy)-ethoxy]-phenyl]-propanoate (1.4 g, 2.30 mmol, 1 equiv) in EtOH (10 mL) was added PTSA (3.97 g, 23.04 mmol, 10 equiv). After stirring at 60 °C for 12 h, the reaction was concentrated to remove the solvent and the residue diluted with EtOAc (80 mL) and washed with H_2_O (20 mL*3), brine (20 mL*2). The organic layer was dried over Na_2_SO_4_, filtered and concentrated to give the residue then purified by silica gel flash column chromatography (PE/EtOAc = 20:1–3:1) to afford methyl 3-[3,5-dichloro-4-[2-(4-hydroxybutoxy)-ethoxy]­phenyl]-propanoate (500 mg, 1.10 mmol, 47.53% yield, 80% purity) as a light brown oil. LCMS: (M+H^+^): 365.0 @ 1.225 min (5–95% ACN in H_2_O, 2.0 min).

Step 5: To a solution of methyl 3-[3,5-dichloro-4-[2-(4-hydroxybutoxy)-ethoxy]-phenyl]-propanoate (500 mg, 1.37 mmol, 1 equiv) and TEA (692.61 mg, 6.84 mmol, 952.70 μL, 5 equiv) in DCM (20 mL) was added MsCl (0.6 g, 5.24 mmol, 405.41 μL, 3.83 equiv). After stirring at 15 °C for 12 h, the mixture was washed with H_2_O (20 mL*2) and brine (20 mL), dried over Na_2_SO_4_, filtered and concentrated to give methyl 3-[3,5-dichloro-4-[2-(4-methylsulfonyloxy butoxy)­ethoxy]­phenyl]­propanoate (500 mg, 1.11 mmol, 80.74% yield, 98% purity) as a yellow solid. LCMS: (M+H^+^): 464.9 @ 1.312 min (5–95% ACN in H_2_O, 3.0 min).

Step 6: A mixture of methyl 3-[3,5-dichloro-4-[2-(4-methylsulfonyloxybutoxy)-ethoxy]-phenyl]-propanoate (0.5 g, 1.13 mmol, 1 equiv), ***k3*** (352.04 mg, 1.13 mmol, 1 equiv), K_2_CO_3_ (467.62 mg, 3.38 mmol, 3 equiv), KI (187.22 mg, 1.13 mmol, 1 equiv) in DMF (10 mL) was degassed and purged with N_2_ for 3 times, and then the mixture was stirred at 80 °C for 2 h under N_2_ atmosphere. The reaction was diluted with H_2_O (30 mL) and extracted with DCM (50 mL*3). The organic layer was washed with brine (30 mL), dried over Na_2_SO_4_, filtered and concentrated to give methyl 2-[3,5-dichloro-4-[4-[2-[2,6-dichloro-4-(3-methoxy-3-oxo-propyl)-phenoxy]-ethoxy]-butoxy]-anilino]-benzoate (0.6 g, 309.38 μmol, 27.43% yield, 34% purity) as a brown solid. LCMS: (M+H^+^): 660.2 @ 1.631 min (5–95% ACN in H_2_O, 2.0 min).

Step 7: To a solution of methyl 2-[3,5-dichloro-4-[4-[2-[2,6-dichloro-4-(3-methoxy-3-oxopropyl)-phenoxy]-ethoxy]-butoxy]-anilino]-benzoate (0.6 g, 909.95 μmol, 1 equiv) in THF (20 mL) and MeOH (10 mL), H_2_O (10 mL) was added LiOH·H_2_O (229.11 mg, 5.46 mmol, 6 equiv). After stirring at 40 °C for 1 h, the reaction was concentrated to give the residue, then diluted with H_2_O (30 mL) and adjusted with 1 N HCl to pH ∼ 2. The mixture was extracted with DCM (50 mL*3). The organic layer was concentrated to give the residue, then purified by prep-HPLC (HCl condition) to afford B2 (294.7 mg, 466.79 μmol,51.30% yield, 100% purity) as an off white solid. LCMS: (M+H^+^): 632.0@ 2.225 min (10–80% ACN in H_2_O, 3.0 min). ^1^H NMR: (DMSO-d6, 400 MHz); δ 9.50 (s, 1H), 7.91 (dd, *J* = 1.5, 7.9 Hz, 1H), 7.44 (ddd, *J* = 1.5, 7.2, 8.5 Hz, 1H), 7.34 (d, *J* = 7.5 Hz, 4H), 7.24 (d, *J* = 7.9 Hz, 1H), 6.91–6.84 (m, 1H), 4.14–4.08 (m, 2H), 3.95 (t, *J* = 6.2 Hz, 2H), 3.78–3.70 (m, 2H), 3.54 (t, *J* = 6.1 Hz, 2H), 2.81–2.73 (m, 2H), 2.55 (t, *J* = 7.5 Hz, 2H), 1.86–1.67 (m, 4H).

### Synthesis of 2-[4-[(3*S*,4*S*)-6-[4-(2-Carboxyethyl)-2,6-dichloro-phenoxy]-3,4-dihydroxy-hexoxy]-3,5-dichloro-anilino]-benzoic acid (B4) and 2-[4-[(3*R*,4*R*)-6-[4-(2-carboxyethyl)-2,6-dichloro-phenoxy]-3,4-dihydroxy-hexoxy]-3,5-dichloro-anilino]-Benzoic Acid (B4A) (Scheme 5)

Step 1: A solution of NaHCO_3_ (130.98 mg, 1.56 mmol, 60.64 μL, 1 equiv), (DHQ)_2_PHAL (60.73 mg, 77.96 μmol, 0.05 equiv), K_2_OsO_4_·2H_2_O (11.49 mg, 31.18 μmol, 0.02 equiv), K_2_CO_3_ (538.72 mg, 3.90 mmol, 2.5 equiv), K_3_[Fe­(CN)_6_] (1.28 g, 3.90 mmol, 1.07 mL, 2.5 equiv) and MeSO_2_NH_2_ (148.31 mg, 1.56 mmol, 1 equiv) in H_2_O (20 mL) and ACN (20 mL) was stirred for 15 min at 15 °C. The mixture was cooled to 0 °C, and compound ***k5*** (1 g, 1.56 mmol, 1 equiv) in THF (20 mL) was added. The reaction was stirred at 15 °C for 12 h. Na_2_SO_3_ (10 g) in H_2_O (50 mL) was added, and the mixture was stirred at 15 °C for 10 min before extraction with EtOAc (100 mL*4). The combined organic layers were washed with brine (50 mL*2), dried over Na_2_SO_4_, and concentrated under vacuum. The residue was then purified by silica gel flash column chromatography (PE/EtOAc = 10:1–1:1) to give methyl 2-[3,5-dichloro-4-[(3S, 4S)-6-[2,6-dichloro-4-(3-methoxy-3-oxo-propyl)­phenoxy]-3,4-dihydroxyhexoxy]­anilino]­benzoate (900 mg, 99.8% purity, 86.2% enantiomeric excess, ee, value) as a colorless oil (SFC: retention time: P1:2.8 min; P2:3.6 min; 86.2% enantiomeric excess, ee, value).

Step 2: Separation of enantiomers. Methyl 2-[3,5-Dichloro-4-[(3*S*,4*S*)-6-[2,6-dichloro-4-(3-methoxy-3-oxo-propyl)­phenoxy]-3,4- dihydroxyhexoxy]­anilino]­benzoate (900 mg, 86.2% ee value) was subjected to SFC (Waters prep-SFC 80Q; column: Chiralpak AD-H, 250*25 mm i.d. 5 μm; mobile phase: A for CO_2_ and B for MeOH (0.1%NH3·H_2_O); gradient: B%=40%; flow rate: 70 mL/min; column temperature: 40 °C; system back pressure:100 bar) to give, both as colorless oils,: methyl 2-[3,5-dichloro-4-[(3*R*,4*R*)-6-[2,6-dichloro-4-(3-methoxy-3-oxo-propyl)­phenoxy]-3,4-dihydroxy-hexoxy]-anilino]­benzoate (P1), (50 mg, 81% purity, 100% ee value) (SFC: retention time: 2.80 min; 100% ee value); methyl 2-[3,5-dichloro-4-[(3*S*,4*S*)-6-[2,6-dichloro-4-(3-methoxy-3-oxo-propyl)­phenoxy]-3,4-dihydroxy-hexoxy]­anilino]­benzoate (P2), (700 mg, 99.7% purity,100% ee value) (SFC: retention time: 3.60 min; 100% ee value).

Step 3: To a solution of P2 (600 mg, 888.39 μmol, 1 equiv) in THF (20 mL), MeOH (10 mL) and H_2_O (10 mL) were added LiOH·H_2_O (223.68 mg, 5.33 mmol, 6 equiv). After stirring at 45 °C for 1.5 h, the reaction was concentrated to remove the solvent and the residue adjusted with 1 N HCl to pH 3–4 and extracted with EtOAc (80 mL*3). The combined organic layers were washed with brine (50 mL*3), dried over Na_2_SO_4_, filtered, and concentrated under reduced pressure to give a residue, then purified by prep-HPLC (HCl condition) to afford B4 (300 mg, 100% purity) as a white solid. LCMS: (M+H^+^): 648.0@ 3.000 min (10–80% ACN in H_2_O, 4.5 min). ^1^H NMR: (Methanol-d4, 400 MHz); δ 8.00 (dd, *J* = 1.5, 7.9 Hz, 1H), 7.44–7.37 (m, 1H), 7.28–7.21 (m, 5H), 6.84 (t, *J* = 7.6 Hz, 1H), 4.27–4.10 (m, 4H), 3.97–3.89 (m, 2H), 2.85 (t, *J* = 7.5 Hz, 2H), 2.63–2.55 (m, 2H), 2.20–2.09 (m, 2H), 2.00 (td, *J* = 6.0, 8.2 Hz, 2H). SFC: (Retention time: 7.777 min; 99.4% ee value).

Step 4: To a solution of P1 (50 mg, 74.03 μmol, 1 equiv) in THF (2 mL), MeOH (1 mL) and H_2_O (1 mL) were added LiOH·H_2_O (18.64 mg, 444.19 μmol, 6 equiv). The reaction was stirred at 45 °C for 1.5 h. The reaction was concentrated to remove the solvent. The residue was adjusted with 1 N HCl to pH 3–4 and extracted with EtOAc (50 mL*3). The combined organic layers were washed with brine (30 mL*2), dried over Na_2_SO_4_, filtered, and concentrated under reduced pressure to give a residue, then purified by prep-HPLC (HCl condition) to afford B4A (15.4 mg, 23.79 μmol, 32.13% yield, 100% purity) as a white solid. LCMS: (M+H^+^): 648.0@ 3.000 min (10–80% ACN in H_2_O, 4.5 min). ^1^H NMR: (Methanol-d4, 400 MHz); δ 8.00 (dd, *J* = 1.5, 8.0 Hz, 1H), 7.45–7.38 (m, 1H), 7.28–7.20 (m, 5H), 6.84 (t, *J* = 7.5 Hz, 1H), 4.27–4.10 (m, 4H), 3.98–3.89 (m, 2H), 2.85 (t, *J* = 7.4 Hz, 2H), 2.65–2.54 (m, 2H), 2.20–2.08 (m, 2H), 2.06–1.93­(m, 2H). SFC: Retention time: 6.656 min; 92.16% ee value.

### Synthesis of 2-[4-[2-[2-[2-(Carboxymethoxy)-ethoxy]-ethoxy]-ethoxy]-3,5-dichloro-anilino]-Benzoic Acid (B5) (Scheme 6)

Step 1: NaH (711.67 mg, 17.79 mmol, 60% purity, 1 equiv) was suspended in toluene (20 mL) and 2-[2-(2- chloroethoxy)­ethoxy]­ethanol (3 g, 17.79 mmol, 1 equiv) was gradually added at −70 °C. Ethyl-2-bromoacetate (2.97 g, 17.79 mmol, 1.97 mL, 1 equiv) was added into the above mixture at −70 °C dropwise. And after 30 min, the reaction mixture was allowed to 15 °C and stirred for 2 h. Acetic acid was added at 0 °C and the solution was neutralized to pH 6–7. After solvent evaporation under reduced pressure, the residue was purified by silica gel flash column chromatography (PE/EtOAc = 10:1–3:1) to afford ethyl 2-[2-[2-(2-chloroethoxy)-ethoxy]-ethoxy]-acetate (3.5 g, 7.70 mmol, 43.25% yield, 56% purity) as a colorless oil. ^1^H NMR: (Chloroform-d, 400 MHz); δ 4.22–4.07 (m, 4H), 3.78–3.60 (m, 12H), 1.31–1.25 (m, 3H).

Step 2: Ethyl 2-[2-[2-(2-Chloroethoxy)-ethoxy]-ethoxy]-acetate (1 g, 3.93 mmol, 1 equiv) was dissolved in NMP (5 mL), then bromoethane (4.28 g, 39.26 mmol, 2.93 mL, 10 equiv) and NaBr (80.79 mg, 785.22 μmol, 25.25 μL, 0.2 equiv) were added to the mixture further heated at 65 °C for 48 h. The reaction was diluted with H_2_O (20 mL) and extracted with EtOAc (50 mL*3). The organic layer was washed with brine (30 mL), dried over Na_2_SO_4_, filtered, and concentrated under reduced pressure to ethyl 2-[2-[2-(2-bromoethoxy)-ethoxy]-ethoxy]-acetate (500 mg, crude) as a brown oil.

Step 3: A mixture of ethyl 2-[2-[2-(2-bromoethoxy)-ethoxy]-ethoxy]-acetate (766.71 mg, 2.56 mmol, 2 equiv), compound ***k3*** (400 mg, 1.28 mmol, 1 equiv), K_2_CO_3_ (177.10 mg, 1.28 mmol, 1 equiv), Bu_4_NI (473.32 mg, 1.28 mmol, 1 equiv) in DMSO (10 mL) was degassed and purged with N_2_ for 3 times, and then stirred at 60 °C for 12 h under N_2_ atmosphere. The mixture was diluted with H_2_O (10 mL) and extracted with EtOAc (30 mL*3). The organic layer was washed with H_2_O (10 mL), 0.1 N HCl (10 mL), and H_2_O (20 mL). It was dried over Na_2_SO_4_, filtered, and concentrated to give the residue then purified by silica gel flash column chromatography (PE/EtOAc = 20:1–3:1) to afford methyl 2-[3,5-dichloro-4-[2-[2-[2-(2-ethoxy-2-oxo-ethoxy)-ethoxy]-ethoxy]-ethoxy]-anilino]-benzoate (430 mg, 88% purity) as a light yellow oil. LCMS: (M+H^+^): 530.0 @ 1.436 min (5–95% ACN in H_2_O, 2.0 min).

Step 4: To a solution of methyl 2-[3,5-dichloro-4-[2-[2-[2-(2-ethoxy-2-oxo-ethoxy)-ethoxy]-ethoxy]-ethoxy]-anilino]-benzoate (380 mg, 716.45 μmol, 1 equiv) in H_2_O (4.2 mL) and THF (5.6 mL) was added LiOH·H_2_O (180.39 mg, 4.30 mmol, 6 equiv) at 15 °C. The mixture was stirred at 15 °C for 12 h and then concentrated to give the residue, which was diluted with H_2_O (20 mL) and extracted with DCM (50 mL*2, discarded). The aqueous layer was adjusted to pH 3–4 with HCl (1N) and extracted with EtOAc (20 mL*3). The organic layer was concentrated to give the residue, then purified by prep-HPLC (HCl condition) to afford B5 (160.1 mg, 99.20% purity) as a light yellow solid. LCMS: (M+H^+^): 486.0 @ 2.435 min (10–80% ACN in H_2_O, 4.5 min). ^1^H NMR: (DMSO-d6, 400 MHz); δ 12.84 (br s, 2H), 9.51 (br s, 1H), 7.91 (dd, *J* = 1.8, 7.9 Hz, 1H), 7.46 (dt, *J* = 1.8, 7.9 Hz, 1H), 7.35 (s, 2H), 7.25 (d, *J* = 8.3 Hz, 1H), 6.88 (t, *J* = 7.5 Hz, 1H), 4.11–4.06 (m, 2H), 4.02 (s, 2H), 3.81–3.74 (m, 2H), 3.63–3.52­(m, 8H).

### Synthesis of 2-[4-[3-[3-[3-(2-Carboxy-ethoxy)-propoxy]-propoxy]-propoxy]-3,5-dichloro-anilino]-Benzoic Acid (B6) (Scheme 7)

Step 1: To a solution of 3-benzyloxypropan-1-ol (50 g, 300.81 mmol, 47.62 mL, 1 equiv) and TEA (60.88 g, 601.63 mmol, 83.74 mL, 2 equiv) in DCM (700 mL) was added TosCl (68.82 g, 360.98 mmol, 1.2 equiv) at 0 °C. The reaction mixture was stirred at 25 °C for 6 h and then concentrated to give a residue further purified by silica gel flash column chromatography (PE/EtOAc = 25:1) to afford 3-benzyloxypropyl 4-methylbenzenesulfonate (81 g, 252.81 mmol, 84.04% yield) as a colorless oil. ^1^H NMR: (Chloroform-d, 400 MHz); δ 7.81 (d, *J* = 8.3 Hz, 2H), 7.40–7.24 (m, 7H), 4.43 (s, 2H), 4.19 (t, *J* = 6.2 Hz, 2H), 3.53 (t, *J* = 5.9 Hz, 2H), 2.53–2.38 (m, 3H), 1.97 (quin, *J* = 6.1 Hz, 2H).

Step 2: To propane-1,3-diol (192.37 g, 2.53 mol, 183.21 mL, 10 equiv) was added NaH (11.12 g, 278.09 mmol, 60% purity, 1.1 equiv) at 0 °C. The mixture was stirred at 0 °C for 0.5 h. Then 3-benzyloxypropyl 4-methylbenzenesulfonate (81 g, 252.81 mmol, 1 equiv) was added at 0 °C. After stirring at 25 °C for 11.5 h, the reaction mixture was diluted with saturated NH_4_Cl solution (600 mL) and extracted with EtOAc (300 mL*2). The combined organic layers were washed with brine (300 mL), dried over Na_2_SO_4_, filtered and concentrated under reduced pressure to give a residue then purified by silica gel flash column chromatography (PE/EtOAc = 8:1–1:1) to afford 3-(3-benzyloxypropoxy)-propan-1-ol (47 g, 209.55 mmol, 82.89% yield) as colorless oil. ^1^H NMR: (Chloroform-d, 400 MHz); δ 7.39–7.27 (m, 5H), 4.51 (s, 2H), 3.76 (t, *J* = 5.6 Hz, 2H), 3.62 (t, *J* = 5.7 Hz, 2H), 3.56 (t, *J* = 6.4 Hz, 4H), 2.47–2.23 (m, 1H), 1.89 (quin, *J* = 6.3 Hz, 2H), 1.83 (quin, *J* = 5.7 Hz, 2H).

Step 3: To a solution of 3-(3-benzyloxypropoxy)-propan-1-ol (37 g, 164.96 mmol, 1 equiv) and TEA (33.38 g, 329.92 mmol, 45.92 mL, 2 equiv) in DCM (400 mL) was added 4-methylbenzenesulfonyl chloride (37.74 g, 197.95 mmol, 1.2 equiv) at 0 °C. The mixture was stirred at 25 °C for 12 h. The reaction mixture was quenched with H_2_O (500 mL), extracted with EtOAc (150 mL*2). The combined organic layers were washed with brine (300 mL), dried over Na_2_SO_4_, filtered and concentrated under reduced pressure to give a residue then purified by silica gel flash column chromatography (PE/EtOAc = 20:1–10:1) to afford 3-(3-benzyloxypropoxy)-propyl 4-methylbenzenesulfonate (57 g, 150.60 mmol, 91.30% yield) as colorless oil. ^1^H NMR: (Chloroform-d, 400 MHz); δ 7.79 (d, *J* = 8.4 Hz, 2H), 7.39–7.28 (m, 7H), 4.49 (s, 2H), 4.13 (t, *J* = 6.2 Hz, 2H), 3.51 (t, *J* = 6.3 Hz, 2H), 3.47–3.39 (m, 4H), 2.44 (s, 3H), 1.89 (quin, *J* = 6.1 Hz, 2H), 1.80 (quin, *J* = 6.3 Hz, 2H).

Step 4: To propane-1,3-diol (114.60 g, 1.51 mol, 109.14 mL, 10 equiv) was added NaH (6.63 g, 165.66 mmol, 60% purity, 1.1 equiv) at 0 °C. The mixture was stirred at 0 °C for 0.5 h. Then 3-(3-benzyloxypropoxy)-propyl 4-methylbenzenesulfonate (57 g, 150.60 mmol, 1 equiv) was added at 0 °C. The mixture was stirred at 60 °C for 11.5 h. The reaction mixture was quenched with saturated NH_4_Cl solution (500 mL) and extracted with EtOAc (300 mL*2). The combined organic layers were washed with brine 300 mL, dried over Na_2_SO_4_, filtered and concentrated under reduced pressure to give a residue then purified by silica gel flash column chromatography (PE/EtOAc = 10:1–1:1) to afford 3-[3-(3-benzyloxypropoxy)-propoxy]-propan-1-ol (32 g, 113.32 mmol, 75.25% yield) as colorless oil. 1H NMR: (Chloroform-d, 400 MHz); δ 7.38–7.25 (m, 5H), 4.51 (s, 2H), 3.76 (t, *J* = 5.7 Hz, 2H), 3.65–3.46 (m, 10H), 2.45 (br s, 1H), 1.93–1.77 (m, 6H).

Step 5: To a solution of 3-[3-(3-benzyloxypropoxy)-propoxy]-propan-1-ol (2 g, 7.08 mmol, 1 equiv) and *tert*-butyl prop-2-enoate (2.72 g, 21.25 mmol, 3.08 mL, 3 equiv) in DCM (40 mL) was added NaOH (16 mL, 50% purity) and TBAI (261.62 mg, 708.28 μmol, 0.1 equiv). The mixture was stirred at 25 °C for 12 h before dilution with H_2_O (80 mL) and extraction with EtOAc (40 mL*2). The combined organic layers were washed with brine (50 mL), dried over Na_2_SO_4_, filtered and concentrated under reduced pressure to give a residue then purified by silica gel flash column chromatography (PE/EtOAc = 20:1–10:1) to afford *tert*-butyl-3-[3-[3-(3-benzyloxypropoxy)-propoxy]-propoxy]-propanoate (2.3 g, 5.60 mmol, 79.10% yield) as colorless oil. ^1^H NMR: (Chloroform-d, 400 MHz); δ 7.31–7.19 (m, 5H), 4.44 (s, 2H), 3.59 (t, *J* = 6.5 Hz, 2H), 3.52–3.38 (m, 12H), 2.41 (t, *J* = 6.5 Hz, 2H), 1.86–1.71 (m, 6H), 1.39 (s, 9H).

Step 6: To a solution of *tert*-butyl 3-[3-[3-(3-benzyloxypropoxy)-propoxy]­propoxy]-propanoate (2.3 g, 5.60 mmol, 1 equiv) in EtOH (20 mL) was added Pd/C (2 g, 10% purity) under Ar. The suspension was degassed under vacuum and purged with H_2_ several times. The mixture was stirred under H_2_ (15 psi) at 25 °C for 12 h. The reaction mixture was filtered and concentrated under reduced pressure to give a residue then purified by silica gel flash column chromatography (PE/EtOAc = 10:1–3:1) to afford *tert*-butyl 3-[3-[3-(3-hydroxypropoxy)-propoxy]-propoxy]-propanoate (1.7 g, 5.31 mmol, 94.70% yield) as colorless oil. ^1^H NMR: (Chloroform-d, 400 MHz); δ 3.76 (t, *J* = 5.6 Hz, 2H), 3.62 (td, *J* = 6.1, 15.5 Hz, 4H), 3.55–3.43 (m, 8H), 2.47 (t, *J* = 6.5 Hz, 2H), 2.40–2.24 (m, 1H), 1.87–1.77 (m, 6H), 1.44 (s, 9H).

Step 7: To a solution of *tert*-butyl 3-[3-[3-(3-hydroxypropoxy)-propoxy]­propoxy]-propanoate (500 mg, 1.56 mmol, 1 equiv) and ***k3*** (487.09 mg, 1.56 mmol, 1 equiv) in THF (7.5 mL) was added PPh_3_ (613.93 mg, 2.34 mmol, 1.5 equiv) and DIAD (473.30 mg, 2.34 mmol, 455.10 μL, 1.5 equiv) at 0 °C. The reaction mixture was warmed to 25 °C and stirred for 12 h before dilution with H_2_O (30 mL) and extraction with EtOAc (20 mL*3). The combined organic layers were washed with brine (30 mL), dried over Na_2_SO_4_, filtered and concentrated under reduced pressure to give a residue then purified by silica gel flash column chromatography (PE/EtOAc = 100:0–80:20) to yield methyl 2-[4-[3-[3-[3-(3-tert-butoxy-3-oxo-propoxy)-propoxy]-propoxy]-propoxy]-3,5-dichloro-anilino]-benzoate (1 g) as yellow oil. ^1^H NMR: (Chloroform-d, 400 MHz); δ 9.42 (s, 1H), 7.98 (dd, *J* = 1.8, 7.9 Hz, 1H), 7.41–7.36 (m, 1H), 7.19 (s, 2H), 6.82 (t, *J* = 7.7 Hz, 1H), 6.33 (br s, 1H), 4.10 (t, *J* = 6.4 Hz, 2H), 3.91 (s, 3H), 3.73–3.63 (m, 4H), 3.52 (tt, *J* = 6.5, 12.8 Hz, 8H), 2.48 (t, *J* = 6.6 Hz, 2H), 2.11 (quin, *J* = 6.2 Hz, 2H), 1.90–1.78 (m, 4H), 1.46 (s, 9H).

Step 8: To a solution of methyl 2-[4-[3-[3-[3-(3-tert-butoxy-3-oxo-propoxy)-propoxy]-propoxy]-propoxy]-3,5-dichloro-anilino]-benzoate (900 mg, 1.46 mmol, 1 equiv) in DCM (10 mL) was added TFA (2 mL). The mixture was stirred at 25 °C for 12 h before dilution with H_2_O (20 mL) and extraction with DCM (20 mL*3). The combined organic layers were washed with brine (30 mL), dried over Na_2_SO_4_, filtered and concentrated under reduced pressure to afford 3-[3-[3-[3-[2,6-dichloro-4-(2-methoxy-carbonyl-anilino)-phenoxy]-propoxy]-propoxy]-propoxy]-propanoic acid (1 g) as dark green oil.

Step 9: To a solution of 3-[3-[3-[3-[2,6-dichloro-4-(2-methoxycarbonylanilino)-phenoxy]-propoxy]-propoxy]-propoxy]-propanoic acid (900 mg, 1.61 mmol, 1 equiv) in THF (35 mL) and MeOH (35 mL) was added LiOH·H_2_O (270.49 mg, 6.45 mmol, 4 equiv) in H_2_O (70 mL). The mixture was stirred at 40 °C for 2 h before dilution with H_2_O (20 mL), acidification to pH= 4 with HCl (1 N), and extraction with EtOAc (20 mL*2). The combined organic layers were washed with brine (20 mL), dried over Na_2_SO_4_, filtered, and concentrated under reduced pressure to give a residue, then purified by prep-HPLC (HCl condition) to afford B6 (326 mg) as a yellow solid. LCMS: (M+H^+^): 544.1 @ 2.000 min (10–80% ACN in H_2_O, 3 min). ^1^H NMR: (Chloroform-d, 400 MHz): δ 9.21 (s, 1H), 8.04 (dd, *J* = 1.5, 8.0 Hz, 1H), 7.41 (ddd, *J* = 1.7, 7.1, 8.6 Hz, 1H), 7.21–7.15 (m, 3H), 6.87–6.79 (m, 1H), 4.12 (t, *J* = 6.1 Hz, 2H), 3.78–3.66 (m, 4H), 3.57 (dt, *J* = 4.3, 6.2 Hz, 4H), 3.51 (dt, *J* = 2.8, 6.3 Hz, 4H), 2.64 (t, *J* = 6.1 Hz, 2H), 2.11 (quin, *J* = 6.2 Hz, 2H), 1.86 (sxt, *J* = 6.3 Hz, 4H).

### Synthesis of 2-[4-[3-[[4-(2-Carboxyethyl)-2,6-dichloro-phenoxy]-methoxy]-propoxy]-3,5-dichloro-anilino]-Benzoic Acid (B8) (Scheme 8)

Step 1: To a mixture of 3-benzyloxypropan-1-ol (5 g, 30.08 mmol, 4.76 mL, 1 equiv) in DMSO (90 mL) was added AcOH (18.97 g, 315.85 mmol, 18.06 mL, 10.5 equiv) and Ac_2_O (67.56 g, 661.79 mmol, 61.98 mL, 22 equiv), under stirring at 65 °C for 6 h. The reaction mixture was partitioned between H_2_O (500 mL) and EtOAc (400 mL). The organic layer was washed with NaHCO_3_ (sat., aq, 250 mL*3), dried over Na_2_SO_4_, and concentrated to give a residue, then purified by silica gel flash column chromatography (PE) to afford 3-(methylsulfanylmethoxy)­propoxymethylbenzene (2.5 g) as a colorless oil. ^1^H NMR: (Chloroform-d, 400 MHz); δ 7.45–7.27 (m, 5H), 4.63 (s, 2H), 4.52 (s, 2H), 3.65 (t, *J* = 6.3 Hz, 2H), 3.62–3.56 (m, 2H), 2.20–2.12 (m, 3H), 1.97–1.88 (m, 2H).

Step 2: To a solution of 3-(methylsulfanylmethoxy)­propoxy-methylbenzene (2.2 g, 9.72 mmol, 1 equiv) in DCM (22 mL) was added sulfuryl dichloride (1.31 g, 9.72 mmol, 971.80 μL, 1 equiv). The mixture was stirred at 25 °C for 50 min before concentration under reduced pressure to remove solvent to afford 3-(chloromethoxy)-propoxymethylbenzene (2.2 g, crude) as yellow oil.

Step 3: To a solution of 3-(chloromethoxy)-propoxymethylbenzene (2.39 g, 11.13 mmol, 1 equiv) and compound ***k1*** (2.77 g, 11.13 mmol, 1 equiv) in DMF (30 mL) was added K_2_CO_3_ (2.31 g, 16.70 mmol, 1.5 equiv). The mixture was stirred at 25 °C for 6 h before dilution with H_2_O (60 mL) and extraction with EtOAc (50 mL*2). The combined organic layers were washed with brine (60 mL), dried over Na_2_SO_4_, filtered and concentrated under reduced pressure to give a residue then purified by silica gel flash column chromatography (PE/EtOAc = 100:0–95:5) to afford methyl 3-[4-(3-benzyloxypropoxymethoxy)-3,5-dichloro-phenyl]-propanoate (2.5 g, 5.85 mmol, 52.55% yield) as colorless oil. ^1^H NMR: (Chloroform-d, 400 MHz); δ 7.39–7.27 (m, 5H), 7.15 (s, 2H), 5.19 (s, 2H), 4.51 (s, 2H), 4.02 (t, *J* = 6.4 Hz, 2H), 3.69 (s, 3H), 3.59 (t, *J* = 6.3 Hz, 2H), 2.90–2.84 (m, 2H), 2.63–2.58 (m, 2H), 1.96 (quin, *J* = 6.4 Hz, 2H).

Step 4: To a solution of methyl 3-[4-(3-benzyloxypropoxymethoxy)-3,5-dichloro-phenyl]-propanoate (1.2 g, 2.81 mmol, 1 equiv) in THF (12 mL) and MeOH (12 mL) was added Pd/C (2 g, 10% purity) under Ar. The suspension was degassed under vacuum and purged with H_2_ several times. The mixture was stirred under H_2_ (15 psi) at 25 °C for 0.5 h. The combined suspension (100 mg scale) was filtered through a pad of Celite and the filter cake was washed with MeOH (800 mL). The combined filtrates were concentrated to give crude product then purified by silica gel flash column chromatography (PE/EtOAc = 10:1–4:1) to afford methyl 3-[3,5-dichloro-4-(3-hydroxypropoxymethoxy)-phenyl]-propanoate (460 mg) as yellow oil. ^1^H NMR: (Chloroform-d, 400 MHz); δ 7.16 (s, 2H), 5.21 (s, 2H), 4.08 (t, *J* = 5.9 Hz, 2H), 3.79 (t, *J* = 5.8 Hz, 2H), 3.71–3.67 (m, 3H), 2.91–2.84 (m, 2H), 2.64–2.58 (m, 2H), 1.91 (quin, *J* = 5.9 Hz, 2H).

Step 5: To a solution of methyl 3-[3,5-dichloro-4-(3-hydroxypropoxy-methoxy)-phenyl]-propanoate (496.91 mg, 1.47 mmol, 1 equiv) and ***k3*** (460 mg, 1.47 mmol, 1 equiv) in THF (10 mL) was added PPh_3_ (579.79 mg, 2.21 mmol, 1.5 equiv) and DIAD (446.98 mg, 2.21 mmol, 429.79 μL, 1.5 equiv). The mixture was stirred at 25 °C for 12 h before concentration under reduced pressure to remove solvent. The residue was purified by silica gel flash column chromatography (PE/EtOAc = 100:0–95:5) to afford methyl 2-[3,5-dichloro-4-[3-[[2,6-dichloro-4-(3-methoxy-3-oxo-propyl)-phenoxy]-methoxy]-propoxy]-anilino]-benzoate (900 mg) as yellow oil.

Step 6: To a solution of methyl 2-[3,5-dichloro-4-[3-[[2,6-dichloro-4-(3-methoxy-3-oxo-propyl)-phenoxy]-methoxy]-propoxy]-anilino]-benzoate (800.00 mg, 1.27 mmol, 1 equiv) in MeOH (4 mL) and THF (4 mL) was added LiOH·H_2_O (319.02 mg, 7.60 mmol, 6 equiv) in H_2_O (8 mL). After stirring at 40 °C for 0.5 h, the reaction mixture (100 mg scale) was acidified to pH= 7 with HCl (2N) and extracted with EtOAc (20 mL*3). The combined organic layers were dried over Na_2_SO_4_, filtered and concentrated under reduced pressure to give a residue then purified by prep-HPLC (FA condition) to afford B8 (99.16% purity) (245.0 mg) as a white solid. LCMS: (M+H^+^): 603.0 @ 2.895 min (10–80% ACN in H_2_O, 4.5 min). ^1^H NMR: (DMSO-d6, 400 MHz); δ 9.52 (br s, 1H), 7.91 (dd, *J* = 1.6, 7.9 Hz, 1H), 7.45 (ddd, *J* = 1.7, 7.1, 8.5 Hz, 1H), 7.38 (s, 2H), 7.34 (s, 2H), 7.25 (dd, *J* = 0.6, 8.4 Hz, 1H), 6.91–6.85 (m, 1H), 5.20 (s, 2H), 4.09–4.00 (m, 4H), 2.81–2.74 (m, 2H), 2.55 (t, *J* = 7.5 Hz, 2H), 2.06 (quin, *J* = 6.4 Hz, 2H).

### Synthesis of 2-[4-[(E)-6-[4-(2-Carboxyethyl)-2,6-dichloro-phenoxy]-hex-3-enoxy]-3,5-dichloro-anilino]-Benzoic Acid (B14) (Scheme 9)

To a solution of compound ***k5*** (synthesis, 300 mg, 467.75 μmol, 1 equiv) in THF (12.5 mL) and MeOH (12.5 mL) was added LiOH·H_2_O (117.76 mg, 2.81 mmol, 6 equiv) in H_2_O (15 mL). The mixture was stirred at 40 °C for 2 h before dilution with H_2_O (30 mL), acidification with HCl (6 N) to pH = 4 and, extraction with EtOAc (30 mL*2). The combined organic layers were washed with brine (50 mL), dried over Na_2_SO_4_, filtered, and concentrated under reduced pressure to give a residue, then purified by prep-HPLC (neutral condition) to afford B14 (58 mg, 93.97 μmol, 20.09% yield, 99.37% purity) as a yellow solid. LCMS: (M+H^+^): 614.0 @ 3.119 min (10–80% ACN in H_2_O, 4.5 min). ^1^H NMR: (Acetone-d6, 400 MHz); δ 8.04 (d, *J* = 7.9 Hz, 1H), 7.49–7.43 (, 1H), 7.36–7.26 (m, 5H), 6.88 (t, *J* = 7.5 Hz, 1H), 5.78 (t, *J* = 3.6 Hz, 2H), 4.09–3.99 (m, 4H), 2.91–2.85 (m, 2H), 2.68–2.62 (m, 2H), 2.61–2.54 (m, 4H).

### Synthesis of 2-[4-[(3*R*,4*R*)-6-[4-(2-Carboxyethyl)-2,6-dichloro-phenoxy]-3,4-dihydroxy-hexoxy]-3-chloro-anilino]-Benzoic Acid (B20) (Scheme 10)

Step 1: To a solution of compound *k6* (1.20 g, 4.32 mmol, 1 equiv), compound *k4* (1.5 g, 4.32 mmol, 1 equiv), PPh_3_ (1.70 g, 6.48 mmol, 1.5 equiv) in THF (25 mL) was added DIAD (1.31 g, 6.48 mmol, 1.26 mL, 1.5 equiv). The mixture was stirred at 15 °C for 12 h. The reaction mixture (500 mg) was concentrated under reduced pressure to remove solvent. The crude product was purified by reversed-phase MPLC column (HCl condition, H_2_O/MeOH = 50:50–0:100) to afford methyl 2-[3-chloro-4-[(E)-6-[2,6-dichloro-4-(3-methoxy-3-oxo-propyl)-phenoxy]-hex-3-enoxy]-anilino]-benzoate (1 g) as a yellow solid.

Step 2: A mixture of K_2_OsO_4_·2H_2_O (11.53 mg, 31.31 μmol, 0.02 equiv), (DHQD)_2_PHAL (60.97 mg, 78.26 μmol, 0.05 equiv), NaHCO_3_ (131.49 mg, 1.57 mmol, 60.88 μL, 1 equiv), MeSO_2_NH_2_ (148.89 mg, 1.57 mmol, 1 equiv), K_2_CO_3_ (540.83 mg, 3.91 mmol, 2.5 equiv) and K_3_[Fe­(CN)_6_] (1.29 g, 3.91 mmol, 1.07 mL, 2.5 equiv) in H_2_O (20 mL) and ACN (20 mL) was stirred for 15 min at 15 °C. The mixture was cooled to 0 °C before adding methyl 2-[3-chloro-4-[(E)-6-[2,6-dichloro-4-(3-methoxy-3-oxo-propyl)-phenoxy]-hex-3-enoxy]-anilino]-benzoate (950 mg, 1.57 mmol, 1 equiv) in THF (120 mL). After stirring at 0 °C for 3 h and 15 °C for 8 h 45 min, the reaction mixture was quenched by the addition of Na_2_SO_3_ 10 g in H_2_O (70 mL) and extracted with EtOAc (60 mL*3). The combined organic layers were washed with brine (70 mL), dried over Na_2_SO_4_, filtered, and concentrated under reduced pressure to give a residue. The residue was purified by silica gel flash column chromatography (PE/EtOAc = 100:0–50:50) to afford methyl 2-[3-chloro-4-[(3*R*,4*R*)-6-[2,6-dichloro-4-(3-methoxy-3-oxo-propyl)-phenoxy]-3,4-dihydroxy-hexoxy]-anilino]-benzoate (760 mg, 1.17 mmol, 75.00% yield) as a yellow solid. SFC: (retention time: 2.22 min; 100% ee value); ^1^H NMR: (DMSO-d6, 400 MHz); δ 9.13 (s, 1H), 7.87 (dd, *J* = 1.4, 8.0 Hz, 1H), 7.42–7.34 (m, 3H), 7.32 (d, *J* = 2.2 Hz, 1H), 7.22–7.13 (m, 2H), 7.00 (d, *J* = 8.4 Hz, 1H), 6.77 (t, *J* = 7.5 Hz, 1H), 4.57 (dd, *J* = 5.7, 17.0 Hz, 2H), 4.17 (br t, *J* = 6.4 Hz, 2H), 4.13–4.03 (m, 2H), 3.85 (s, 3H), 3.65 (br s, 2H), 3.58 (s, 3H), 2.84–2.77 (m, 2H), 2.69–2.61 (m, 2H), 2.05–1.91 (m, 2H), 1.88–1.74 (m, 2H).

Step 3: To a solution of methyl 2-[3-chloro-4-[(3*R*,4*R*)-6-[2,6-dichloro-4-(3-methoxy-3-oxo-propyl) phenoxy]-3,4-dihydroxy-hexoxy]-anilino]-benzoate (690 mg, 1.08 mmol, 1 equiv) in THF (30 mL) and MeOH (15 mL) was added LiOH·H_2_O (271.03 mg, 6.46 mmol, 6 equiv) in H_2_O (15 mL). After stirring at 40 °C for 6 h, the reaction mixture (70 mg) was diluted with H_2_O (40 mL), acidified to pH = 6 with HCl (1 N) before extraction with EtOAc (30 mL*3). The combined organic layers were washed with brine (60 mL), dried over Na_2_SO_4_, filtered and concentrated under reduced pressure to give a residue then purified by prep-HPLC (Phenomenex Luna C18 200*40 mm*10 μ m; mobile phase: [water­(0.05% HCl)-ACN]; B%: 35%–65%,10 min) to afford B20 (547 mg) as a light yellow solid. LCMS: (M+H^+^): 612.1@ 2.603 min (10–80% ACN in H_2_O, 4.5 min). ^1^H NMR: (DMSO-d6, 400 MHz); δ 13.26–11.93 (m, 2H), 9.44 (s, 1H), 7.88 (dd, *J* = 1.6, 7.9 Hz, 1H), 7.40–7.31 (m, 4H), 7.22–7.13 (m, 2H), 7.00 (d, *J* = 8.6 Hz, 1H), 6.78–6.71 (m, 1H), 4.57 (br s, 2H), 4.17 (br t, *J* = 6.4 Hz, 2H), 4.13–4.02 (m, 2H), 3.65 (br d, *J* = 7.6 Hz, 2H), 2.81–2.73 (m, 2H), 2.55 (t, *J* = 7.6 Hz, 2H), 2.05–1.92 (m, 2H), 1.88–1.73 (m, 2H). SFC: (retention time: 4.56 min; 100% ee value).

### Synthesis of 2-[4-[6-[4-(2-Carboxyethyl)-2-chloro-phenoxy]-3,4-dihydroxy-hexoxy]-3,5-dichloro- anilino]-Benzoic Acid (B21) (Scheme 11)

Step 1: To a mixture of compound ***k7*** (1 g, 3.20 mmol, 1 equiv), compound *k3* (997.95 mg, 3.20 mmol, 1 equiv) and PPh_3_ (1.68 g, 6.39 mmol, 2 equiv) in THF (10 mL) was added DIAD (1.29 g, 6.39 mmol, 1.24 mL, 2 equiv) at 0 °C. The reaction mixture was stirred at 15 °C for 12 h before concentration to give the residue then purified by reversed-phase HPLC (MeOH/0.1% TFA condition) to afford methyl 2-[3,5-dichloro-4-[(E)-6-[2-chloro-4-(3-methoxy-3-oxo-propyl)-phenoxy]-hex-3-enoxy]-anilino]-benzoate (1 g, 1.59 mmol, 49.66% yield, 96.35% purity) as a yellow solid.

Step 2: A solution of NaHCO_3_ (166.10 mg, 1.98 mmol, 76.90 μL, 1 equiv), (DHQD)_2_PHAL (77.01 mg, 98.86 μmol, 0.05 equiv), K_2_OsO_4_·2H_2_O (14.57 mg, 39.54 μmol, 0.02 equiv), K_2_CO_3_ (683.17 mg, 4.94 mmol, 2.5 equiv), K_3_[Fe­(CN)_6_] (1.63 g, 4.94 mmol, 1.36 mL, 2.5 equiv) and MeSO_2_NH_2_ (188.07 mg, 1.98 mmol, 1 equiv) in water (20 mL) and ACN (20 mL) at 15 °C was stirred for 15 min. The clear solution was cooled to 0 °C and a solution of methyl 2-[3,5-dichloro-4-[(E)-6-[2-chloro-4-(3-methoxy-3-oxo-propyl)-phenoxy]-hex-3-enoxy]-anilino]-benzoate (1.2 g, 1.98 mmol, 1 equiv) in THF (20 mL) was added. The suspension was diluted with THF (200 mL) before stirring at 15 °C for 12 h. Na_2_SO_3_ (5 g) in H_2_O (50 mL) was added, and the mixture stirred at 15 °C for 10 min before extraction with EtOAc (70 mL*4). The combined organic layers were washed with brine (30 mL), dried over Na_2_SO_4_ and concentrated under vacuum to give a residue then purified by silica gel flash column chromatography (PE/EtOAc = 20:1–1:1) to afford methyl 2-[3,5-dichloro-4- [(3*R*,4*R*)-6-[2-chloro-4-(3-methoxy-3-oxo-propyl)­phenoxy]-3,4-dihydroxyhexoxy]­anilino]­benzoate (1.0 g, 1.51 mmol, 76.54% yield, 97% purity) as a light yellow solid. The product was checked by chiral SFC (retention time: 3.242 min, 97.8% ee) and ^1^H NMR: (400 MHz, DMSO-d6); δ 9.12 (s, 1H), 7.89 (dd, *J* = 1.5, 8.0 Hz, 1H), 7.50–7.43 (m, 1H), 7.31 (s, 2H), 7.28–7.22 (m,2H), 7.14–7.10 (m, 1H), 7.06–7.02 (m, 1H), 6.93–6.87 (m, 1H), 4.55 (dd, *J* = 5.8, 13.9 Hz, 2H), 4.16–4.05 (m,4H), 3.84 (s, 3H), 3.67–3.59 (m, 2H), 3.56 (s, 3H), 2.80–2.73 (m, 2H), 2.62–2.56 (m, 2H), 1.99–1.72 (m, 4H).

Step 3: To a solution of methyl 2-[3,5-dichloro-4-[6-[2-chloro-4-(3-methoxy-3-oxo-propyl)-phenoxy]-3,4-dihydroxyhexoxy]-anilino]-benzoate (1 g, 1.56 mmol, 1 equiv) in THF (30 mL) was added LiOH·H_2_O (327.36 mg, 7.80 mmol, 5 equiv). After stirring at 40 °C for 2 h, the reaction was diluted with H_2_O (10 mL) and extracted with EtOAc (10 mL*2). The aqueous layers were acidified with HCl (1N) to pH 5–6. The suspension was extracted with 2-methyltetrahydrofuran (50 mL*3) and washed with brine (20 mL), dried over Na_2_SO_4_ and filtered and concentrated to give the residue then purified by prep-HPLC (HCl condition) to afford B21 (661.1 mg, 1.08 mmol, 99.73% purity, 69.14% yield) as a white solid. LCMS: (M+H^+^): 614.1 @ 1.986 min (5–90% ACN in H_2_O, 4.5 min). ^1^H NMR: (400 MHz, Methanol-d4); δ 8.00 (dd, *J* = 1.6, 7.9 Hz, 1H), 7.41 (dt, *J* = 1.7, 7.8 Hz, 1H), 7.26–7.20 (m, 4H), 7.11 (dd, *J* = 2.0, 8.4 Hz, 1H), 7.00 (d, *J* = 8.4 Hz, 1H), 6.84 (t, *J* = 7.6 Hz, 1H), 4.27–4.11 (m, 4H), 3.93–3.86 (m, 2H), 2.83 (t, *J* = 7.5 Hz, 2H), 2.60–2.51 (m, 2H), 2.19–1.93 (m, 4H). SFC: (retention time: 3.10 min,100% ee).

### Synthesis of 2-[4-[(3*R*,4*R*)-6-[4-(2-Carboxyethyl)-2-chloro-phenoxy]-3,4-dihydroxy-hexoxy]-3-chloro-anilino]­Benzoic Acid (B22) (Scheme 12)

Step 1: To a solution of compound ***k6*** (887.83 mg, 3.20 mmol, 1 equiv), compound ***k7*** (1 g, 3.20 mmol, 1 equiv) and PPh_3_ (1.26 g, 4.80 mmol, 1.5 equiv) in THF (20 mL) was added DIAD (969.72 mg, 4.80 mmol, 932.42 μL, 1.5 equiv). After stirring at 15 °C for 12 h, the combined reaction mixtures (100 mg scale) were concentrated under reduced pressure to remove solvent. The crude product was purified by reversed-phase MPLC column (HCl condition, H_2_O/MeOH = 50:50–0:100) to afford methyl 2-[3-chloro-4-[(E)-6-[2-chloro-4-(3-methoxy-3-oxo-propyl)-phenoxy]-hex-3-enoxy]-anilino]-benzoate (1.15 g) as a yellow solid.

Step 2: A mixture of K_2_OsO_4_·2H_2_O (12.90 mg, 35.00 μmol, 0.02 equiv), (DHQD)_2_PHAL (68.16 mg, 87.50 μmol, 0.05 equiv), NaHCO_3_ (147.02 mg, 1.75 mmol, 68.06 μL, 1 equiv), MeSO_2_NH_2_ (166.46 mg, 1.75 mmol, 1 equiv), K_2_CO_3_ (604.67 mg, 4.38 mmol, 2.5 equiv) and K_3_[Fe­(CN)_6_] (1.44 g, 4.38 mmol, 1.20 mL, 2.5 equiv) in H_2_O (40 mL) and ACN (40 mL) was stirred for 15 min at 15 °C. The mixture was cooled to 0 °C before adding and methyl 2-[3-chloro-4-[(E)-6-[2-chloro-4-(3-methoxy-3-oxo-propyl)-phenoxy]-hex-3-enoxy]-anilino]-benzoate (1 g, 1.75 mmol, 1 equiv) in THF (80 mL).

After stirring at 0 °C for 3 h and at 15 °C for 8 h 45 min, the reaction mixture was quenched by addition Na_2_SO_3_ 10 g in H_2_O (70 mL) and extracted with EtOAc (60 mL*3). The combined organic layers were washed with brine (70 mL), dried over Na_2_SO_4_, filtered and concentrated under reduced pressure to give a residue then purified by silica gel flash column chromatography (PE/EtOAc = 100:0–50:50) to afford methyl 2-[3-chloro-4-[(3*R*,4*R*)-6-[2-chloro-4-(3-methoxy-3-oxo-propyl)-phenoxy]-3,4-dihydroxy-hexoxy]-anilino]-benzoate (880 mg, 1.42 mmol, 81.25% yield, 98% purity) as a yellow solid. SFC: (retention time: 2.22 min; 98.8% ee value); ^1^H NMR: (DMSO-d6, 400 MHz); δ 9.13 (s, 1H), 7.87 (dd, *J* = 1.5, 8.0 Hz, 1H), 7.41–7.35 (m, 1H), 7.32 (d, *J* = 2.3 Hz, 1H), 7.27 (d, *J* = 2.1 Hz, 1H), 7.21–7.11 (m, 3H), 7.06–7.03 (m, 1H), 7.00 (d, *J* = 8.4 Hz, 1H), 6.79–6.74 (m, 1H), 4.59 (dd, *J* = 2.3, 5.9 Hz, 2H), 4.16 (td, *J* = 6.5, 13.0 Hz, 4H), 3.85 (s, 3H), 3.66–3.60 (m, 2H), 3.57 (s, 3H), 2.80–2.74 (m, 2H), 2.62–2.57 (m, 2H), 1.98–1.89 (m, 2H), 1.85–1.75 (m, 2H).

Step 3: To a solution of methyl 2-[3-chloro-4-[(3*R*,4*R*)-6-[2-chloro-4-(3-methoxy-3-oxo-propyl) phenoxy]-3,4-dihydroxy-hexoxy]-anilino]-benzoate (800.00 mg, 1.32 mmol, 1 equiv) in THF (30 mL) and MeOH (15 mL) was added LiOH·H_2_O (332.09 mg, 7.91 mmol, 6 equiv) in H_2_O (15 mL). The mixture was stirred at 40 °C for 6 h before dilution with H_2_O (40 mL), acidification to pH 6 with HCl (1 N), and extraction with EtOAc (30 mL*3). The combined organic layers were washed with brine (60 mL), dried over Na_2_SO_4_, filtered and concentrated under reduced pressure to give a residue then was purified by prep-HPLC (Phenomenex Luna C18 200*40 mm*10 μm; mobile phase: [water­(0.05%HCl)-ACN]; B%: 35%–55%,10 min,) to afford B22 (420 mg) as a white solid. LCMS: (M+H^+^): 578.0@ 2.860 min (10–80% ACN in H_2_O, 4.5 min). ^1^H NMR: (Methanol-d4, 400 MHz); δ 7.96 (dd, *J* = 1.5, 8.0 Hz, 1H), 7.31 (dt, *J* = 1.6, 7.8 Hz, 1H), 7.24–7.18 (m, 2H), 7.14–7.06 (m, 3H), 7.00 (dd, *J* = 8.4, 14.0 Hz, 2H), 6.74–6.67 (m, 1H), 4.26–4.15 (m, 4H), 3.90–3.82 (m, 2H), 2.86–2.78 (m, 2H), 2.55 (t, *J* = 7.5 Hz, 2H), 2.16–2.05 (m, 2H), 2.04–1.93 (m, 2H). SFC: retention time: 5.24 min; 100% ee value.

### Synthesis of 5-[4-[2-[3-[4-(2-Carboxyethyl)-2,6-dichloro-phenoxy]-propoxy]-ethoxy]-3,5-dichloro-anilino]-Pyridazine-4-Carboxylic acid (B24) (Scheme 13)

Step 1:4-Bromo-2,6-dichloro-phenol (1 g, 4.13 mmol, 1 equiv) was dissolved in Acetone (20 mL). K_2_CO_3_ (571.34 mg, 4.13 mmol, 1eq) and BnBr (707.05 mg, 4.13 mmol, 491.01 μL, 1 equiv) were added to the suspension at 25 °C. After stirring at 25 °C for 6 h, the suspension was filtered, and the cake washed with acetone (100 mL). The filtrate was concentrated to give 2-benzyloxy-5-bromo-1,3-dichloro-benzene (1.2 g, crude) as a slight yellow solid. ^1^H NMR: (400 MHz, DMSO-d6); δ 7.82 (s, 2H), 7.53–7.49 (m, 2H), 7.45–7.37 (m, 3H), 5.02 (s, 2H).

Step 2: The solution of H_2_SO_4_ (6.44 g, 65.66 mmol, 3.5 mL, 18.27 equiv) in MeOH (15 mL) was cooled to 0 °C and the suspension of 5-aminopyridazine-4-carboxylic acid (500 mg, 3.59 mmol, 1 equiv) in MeOH (2 mL) was slowly added at 0 °C. The mixture was then heated at 75 °C for 12 h, cooled to room temperature, poured into ice–water (100 mL) and neutralized with solid Na_2_CO_3_ to pH 7–8. The aqueous mixture was extracted with 2-Me THF (100 mL*10) and the organic extracts were dried over Na_2_SO_4_, filtered, and concentrated to give the crude product then purified by silica gel flash column chromatography (MeOH/EtOAc = 100:0–80:20) to give methyl 5-aminopyridazine-4-carboxylate (500 mg, 3.27 mmol, 90.84% yield) as a slight yellow solid. ^1^H NMR: (400 MHz, Methanol-d4); δ 8.90 (s, 1H), 8.70 (d, *J* = 0.6 Hz, 1H), 3.94 (s, 3H).

Step 3: In a glovebox, methyl 5-aminopyridazine-4-carboxylate (170 mg, 1.11 mmol, 1 equiv), 2-benzyloxy-5-bromo-1,3-dichloro-benzene (368.58 mg, 1.11 mmol, 1 equiv), [2-(2-aminophenyl)-phenyl]-methyl sulfonyloxy-palladium;di*tert*-butyl-[2-(2,4,6-tri-isopropyl-phenyl)-phenyl]-phosphane (88.18 mg, 111.01 μmol, 0.1 equiv) were taken up into a sealed bottle with 2-methyl-2-butanol (5.5 mL) and, NaOtBu (2 M, 1.11 mL, 2 equiv) was added to the suspension. After removing the sealed bottle from the glovebox, it was heated at 100 °C for 18 h. The reaction mixture was diluted with H_2_O (100 mL) and acidified with HCl (1M) to pH 5–6 then extracted with 2-MeTHF (70 mL*5). The combined organic phase was washed with saturated brine (50 mL*2), dried over anhydrous Na_2_SO_4_, filtered, and concentrated. Methyl 5-(4-benzyloxy-3,5-dichloro- anilino)-pyridazine-4-carboxylate (1.6 g, crude, 11.9% purity) was obtained as a brown-black oil without further purification.

Step 4: After cooling H_2_SO_4_ (18.40 g, 187.60 mmol, 10 mL, 45.75 equiv) in MeOH (15 mL) to 0 °C, a solution of 5-(4-benzyloxy-3,5-dichloro-anilino)-pyridazine-4-carboxylic acid (1.6 g, 4.10 mmol, 1 equiv) in THF (10 mL) was added. After stirring the mixture at 75 °C for 12 h, it was cooled to room temperature, poured into ice–water (100 mL) and neutralized with NaOH (1M) to pH 7–8. The aqueous mixture was extracted with 2-Me THF (100 mL*10) and the organic extracts were dried over Na_2_SO_4_, filtered, and concentrated to give the crude product then purified by silica gel flash column chromatography (PE/EtOAc/MeOH = 50:50:0–0:90:10) to give methyl 5-(4-benzyloxy-3,5-dichloro -anilino)-pyridazine-4-carboxylate (170 mg, 365.87 μmol, 8.92% yield, 87% purity) as a brown solid. LCMS: (M+H^+^): 314.0 @ 0.656 min (5–95% ACN in H_2_O, 2.0 min).

Step 5: A mixture of methyl 5-(3,5-dichloro-4-hydroxy-anilino)-pyridazine-4-carboxylate (180 mg, 573.02 μmol, 1 equiv), methyl (E)-3-[3,5-dichloro-4-[3-[2-(p-tolylsulfonyloxy)-ethoxy]-propoxy]-phenyl]-prop-2-enoate (288.45 mg, 573.02 μmol, 1 equiv), KI (95.12 mg, 573.02 μmol, 1 equiv), K_2_CO_3_ (237.59 mg, 1.72 mmol, 3 equiv) in DMF (5 mL) was degassed and purged with N_2_ for 3 times, and then the mixture was stirred at 25 °C for 20 h under N_2_ atmosphere. The reaction was filtered and concentrated to give the residue. The reaction was diluted with H_2_O (50 mL) and then extracted with EtOAc (50 mL*3). The combined organic phase was washed with saturated brine (50 mL*2), dried over anhydrous Na_2_SO_4_, filtered, and concentrated under vacuum. The residue was purified by silica gel flash column chromatography (PE/EtOAc = 100:0–50:50) to give methyl 5-[3,5-dichloro-4-[2-[3-[2,6-dichloro-4-(3-methoxy-3-oxo-propyl)-phenoxy]-propoxy]-ethoxy]-anilino]-pyridazine-4-carboxylate (120 mg, 94.54 μmol, 16.50% yield, 51% purity) as slight yellow solid. LCMS: (M+H^+^): 648.0 @ 1.944 min (5–95% ACN in H_2_O, 4.5 min).

Step 6: To a solution of methyl 5-[3,5-dichloro-4-[2-[3-[2,6-dichloro-4-(3-methoxy-3-oxopropyl) phenoxy]­propoxy]­ethoxy]­anilino]­pyridazine-4-carboxylate (120 mg, 185.38 μmol, 1 equiv) in MeOH (3 mL)/H_2_O­(3 mL)/THF (3 mL) was added LiOH·H_2_O (77.78 mg, 1.85 mmol, 10 equiv). The reaction was stirred at 25 °C for 12 h before acidification with HCl (1M) to pH 5–6 and extraction with EtOAc (50 mL*3). The combined organic phase was washed with saturated brine (50 mL*2), dried over anhydrous Na_2_SO_4_, filtered, and concentrated under vacuum. The residue was purified by prep-HPLC (column: Phenomenex Luna C18 150*30 mm*5 μm; mobile phase: [water (0.04% HCl)-ACN]; B%: 35%–65%,10 min;) to give B24 (23.2 mg, 37.09 μmol, 20.01% yield, 99% purity) as a yellow solid. LCMS: (M+H^+^): 620.1 @ 2.11 min (5–95% ACN in H_2_O, 6.0 min). ^1^H NMR: (400 MHz, DMSO-d6); δ 11.20 (br s, 1H), 9.27 (s, 1H), 8.88 (s, 1H), 7.65 (s, 2H), 7.36 (s, 2H), 4.22–4.17 (m, 2H), 4.03 (t, *J* = 6.4 Hz, 2H), 3.81–3.77 (m, 2H), 3.72–3.68 (m, 3H), 2.79–2.74 (m, 2H), 2.57–2.54 (m, 2H), 2.00 (quin, *J* = 6.3 Hz,2H).

### Synthesis of 4-[4-[2-[3-[4-(2-Carboxyethyl)-2,6-dichloro-phenoxy]-propoxy]-ethoxy]-3,5-dichloro-anilino]-Pyridazine-3-Carboxylic acid (B25) (Scheme 14)

Step 1: To a mixture of 2,6-dichloro-4-nitro-phenol (5 g, 24.04 mmol, 1 equiv) and Cs_2_CO_3_ (23.50 g, 72.12 mmol, 3 equiv) in NMP (50 mL) was added BnBr (4.93 g, 28.85 mmol, 3.43 mL, 1.2 equiv) and stirred at 20 °C for 12 h. The reaction mixture was diluted with H_2_O (100 mL) and extracted with EtOAC (70 mL*3). The combined organic layers were washed with brine (50 mL*2), dried over Na_2_SO_4_, filtered and concentrated under reduced pressure to give a residue which was purified by silica gel flash column chromatography (PE/EtOAc = 100:0–98:2) to afford 2-benzyloxy-1,3-dichloro-5-nitro-benzene (6 g, 20.13 mmol, 83.72% yield) as a brown oil.

Step 2: To a solution of 2-benzyloxy-1,3-dichloro-5-nitro-benzene (6 g, 20.13 mmol, 1 equiv) in EtOH (40 mL)/H_2_O (8 mL) was added NH_4_Cl (1.08 g, 20.13 mmol, 1 equiv). Fe (5.62 g, 100.63 mmol, 5 equiv) was added in batches. After stirring at 60 °C for 2 h, the reaction was filtered through the Celite, washed with MeOH (50 mL), and concentrated. The residue was purified by silica gel flash column chromatography (PE/EtOAc = 100:0–80:20) to afford 4-benzyloxy-3,5-dichloro-aniline (4.2 g, 15.66 mmol, 77.83% yield) as a white solid. ^1^H NMR: (400 MHz, Chloroform-d); δ 7.56 (d, *J* = 7.1 Hz, 2H), 7.44–7.33 (m, 3H), 6.64 (s, 2H), 4.96 (s, 2H), 3.64 (br s, 2H).

Step 3: Ethyl 4,6-dichloropyridazine-3-carboxylate (350 mg, 1.58 mmol, 1 equiv), 4-benzyloxy-3,5- dichloro-aniline (424.58 mg, 1.58 mmol, 1 equiv), and DIPEA (409.28 mg, 3.17 mmol, 551.59 μL, 2 equiv) were taken up into a microwave tube in ACN (10 mL). The sealed tube was heated at 140 °C for 12 h. The reaction was concentrated to give the residue, then purified by silica gel flash column chromatography (PE/EtOAc = 100:0–75:25) to afford ethyl 4-(4-benzyloxy-3,5-dichloro-anilino)-6-chloro-pyridazine-3- carboxylate (200 mg, 397.60 μmol, 25.11% yield, 90% purity) as a brown solid. ^1^H NMR: (400 MHz, DMSO-d6); δ 9.49 (s, 1H), 7.59 (s, 2H), 7.55 (br d, *J* = 6.6 Hz, 2H), 7.47–7.36 (m, 3H), 7.19 (s, 1H), 5.05­(s, 2H), 4.48–4.35 (m, 2H), 1.37 (t, *J* = 7.1 Hz, 3H).

Step 4: To a solution of ethyl 4-(4-benzyloxy-3,5-dichloro-anilino)-6-chloro-pyridazine-3- carboxylate (200 mg, 441.78 μmol, 1 equiv), 4,4,5,5-tetramethyl-2-(4,4,5,5-tetramethyl-1,3,2- dioxaborolan-2-yl)-1,3,2-dioxaborolane (224.37 mg, 883.55 μmol, 2 equiv) in dioxane (20 mL) was added Pd­(dppf)­Cl_2_·CH_2_Cl_2_ (72.15 mg, 88.36 μmol, 0.2 equiv) and KOAc (130.07 mg, 1.33 mmol, 3 equiv). After stirring at 90 °C for 6 h, the reaction was filtered and concentrated to give the residue then purified by silica gel flash column chromatography (PE/EtOAc = 100:0–30:70) and, by prep-TLC (SiO_2_, PE/EtOAc = 2:3) to give ethyl 4-(4-benzyloxy-3,5-dichloro- anilino)-pyridazine-3-carboxylate (110 mg) as a brown solid.

Step 5: To a solution of ethyl 4-(4-benzyloxy-3,5-dichloro-anilino)-pyridazine-3-carboxylate (55 mg, 131.49 μmol, 1 equiv) in THF (10 mL) was added Pd/C (5 mg, 10% purity) under N_2_. The suspension was degassed under vacuum and purged with H_2_ several times. The mixture was stirred under H_2_ (15 psi) at 25 °C for 20 min. The reaction mixture was filtered and the filter was concentrated to give 75 mg (60.9% purity). The crude product was triturated with MTBE (10 mL) at 25 °C for 2 min and filtered to give ethyl 4-(3,5-dichloro-4-hydroxy- anilino)-pyridazine-3-carboxylate (54 mg, 116.84 μmol, 44.43% yield, 71% purity) as a light yellow solid.

Step 6: A mixture of ethyl 4-(3,5-dichloro-4-hydroxy-anilino)-pyridazine-3-carboxylate (56 mg, 170.65 μmol, 1 equiv), *Tos-k2* (103.50 mg, 204.78 μmol, 1.2 equiv), KI (28.33 mg, 170.65 μmol, 1 equiv), K_2_CO_3_ (70.76 mg, 511.96 μmol, 3 equiv) in DMF (1 mL) was degassed and purged with N_2_ for 3 times, and then the mixture was stirred at 80 °C for 2 h under N_2_ atmosphere. The reaction mixture was filtered and concentrated to give the residue, then diluted with H_2_O (50 mL) and extracted with 2-Me THF (50 mL*5). The combined organic phase was washed with saturated brine (50 mL*2), dried over anhydrous Na_2_SO_4_, filtered and concentrated under vacuum to give the residue (200 mg, 17% purity) then purified by silica gel flash column chromatography (PE/EtOAc = 100:0–0:100) and, by prep-TLC (SiO_2_, DCM/MeOH = 20:1) to afford: batch 1, ethyl 4-[3,5-dichloro-4-[2-[3-[2,6-dichloro-4-(3-methoxy-3-oxo-propyl)­phenoxy] propoxy]­ethoxy]­anilino]­pyridazine-3-carboxylate (∼10 mg, 83% purity) as slight yellow solid; batch 2, ethyl 4-[3,5-dichloro-4-[2-[3-[2,6-dichloro-4-(3-methoxy-3-oxo-propyl)­phenoxy] propoxy]­ethoxy]­anilino]­pyridazine-3-carboxylate (∼20 mg, 31% purity) as a brown oil.

Step 7: To a solution of ethyl 4-[3,5-dichloro-4-[2-[3-[2,6-dichloro-4-(3-methoxy-3-oxopropyl)-phenoxy]-propoxy]-ethoxy]-anilino]-pyridazine-3-carboxylate (10 mg, 15.12 μmol, 1 equiv) in MeOH (1 mL)/H_2_O (1 mL)/dioxane (1 mL) was added LiOH·H_2_O (6.35 mg, 151.20 μmol, 10 equiv). After stirring at 40 °C for 0.5 h, the reaction was acidified with HCl (1M) to pH 5–6 and directly purified by prep-HPLC (column: Phenomenex Luna C18 150*30 mm*5 μm; mobile phase: [water (0.04% HCl)-ACN]; B%: 20%–50%,10 min;) to afford B25 (6.6 mg, 98.8% purity) as an off-white solid. LCMS: (M+H^+^): 620.0 @ 2.123 min (15–100% ACN in H_2_O, 4.5 min). ^1^H NMR: (400 MHz, Methanol-d4); δ 8.77 (d, *J* = 7.2 Hz, 1H), 7.53 (s, 2H), 7.41 (d, *J* = 7.3 Hz, 1H), 7.25 (s, 2H), 4.33–4.27 (m, 2H), 4.08 (t, *J* = 6.3 Hz, 2H), 3.92–3.85 (m, 2H), 3.79 (t, *J* = 6.2 Hz, 2H), 2.88–2.81 (m, 2H), 2.63–2.55 (m, 2H), 2.08 (quin, *J* = 6.2 Hz, 2H).

### Synthesis of 3-[4-[2-[3-[4-(2-Carboxyethyl)-2,6-dichloro-phenoxy]-propoxy]-ethoxy]-3,5-dichloro-anilino]-Pyridine-4-Carboxylic Acid (B26) (Scheme 15)

Step 1: A suspension of methyl 3-bromopyridine-4-carboxylate (101.42 mg, 469.46 μmol, 1.2 equiv), compound *k8* (200 mg, 391.22 μmol, 1 equiv), Cs_2_CO_3_ (178.45 mg, 547.71 μmol, 1.4 equiv), Pd_2_(dba)_3_ (71.65 mg, 78.24 μmol, 0.2 equiv) and Xantphos (135.82 mg, 234.73 μmol, 0.6 equiv) in anhydrous toluene (10 mL) was heated to 135 °C for 2 h under microwave irradiation. The reaction was filtered and concentrated to give the residue, then diluted with H_2_O (50 mL) and extracted with EtOAc (50 mL*3). The combined organic phase was washed with saturated brine (50 mL*2), dried over anhydrous Na_2_SO_4_, filtered, and concentrated under vacuum.

The residue was purified by silica gel flash column chromatography (PE/EtOAc = 100:0–75:25) to give methyl 3-[3,5-dichloro-4-[2-[3-[2,6-dichloro-4-(3-methoxy-3-oxo-propyl)­phenoxy]­propoxy]­ethoxy]­anilino]­pyridine-4-carboxylate (310 mg) as a light brown oil. ^1^H NMR: (400 MHz, Chloroform-d); δ 9.01 (s, 1H), 8.66 (s, 1H), 8.11 (d, *J* = 5.1 Hz, 1H), 7.70 (d, *J* = 5.1 Hz, 1H), 7.21 (s,2H), 7.12 (s, 2H), 4.25–4.17 (m, 2H), 4.12 (t, *J* = 6.3 Hz, 2H), 3.95 (s, 3H), 3.91–3.86 (m, 2H), 3.85 −3.80 (m, 2H), 3.68 (s, 3H),2.90–2.82 (m, 2H), 2.64–2.55 (m, 2H), 2.19–2.09 (m, 2H).

Step 2: To a solution of methyl 3-[3,5-dichloro-4-[2-[3-[2,6-dichloro-4-(3-methoxy-3-oxopropyl)-phenoxy]-propoxy]-ethoxy]-anilino]-pyridine-4-carboxylate (310 mg, 479.62 μmol, 1 equiv) in THF (5 mL)/MeOH (5 mL)/H_2_O (2 mL) was added LiOH·H_2_O (201.27 mg, 4.80 mmol, 10 equiv). The reaction was stirred at 25 °C for 12 h. Subsequent acidification with HCl (1M) to pH 5–6 led to a solid formation. The suspension was filtered to give the cake then dissolved in DMSO/MeOH/THF/H_2_O (1:1:1:1, 10 mL), adjusted with NaHCO_3_ (sat., aq.) to pH 8–9. The residue was purified by prep-HPLC (column: Phenomenex Luna C18 150*30 mm*5 μm; mobile phase: [water (0.04% HCl)-ACN]; B%: 40%–70%, 10 min, to give B26 (100.5 mg, 161.68 μmol, 33.71% yield, 99.47% purity)) as a yellow solid. LCMS: (M+H^+^): 618.9 @ 1.815 min (15–100% ACN in H_2_O, 4.5 min). ^1^H NMR: (400 MHz, DMSO-d6); δ 9.10 (s, 1H), 8.59 (s, 1H), 8.15 (d, *J* = 5.1 Hz, 1H), 7.78 (d, *J* = 5.1 Hz, 1H), 7.40 (s, 2H), 7.35­(s, 2H), 4.15–4.09 (m, 2H), 4.02 (t, *J* = 6.3 Hz, 2H), 3.79–3.75 (m, 2H), 3.68 (br t, *J* = 6.2 Hz, 2H), 2.79–2.72 (m, 2H), 2.56–2.54 (m, 2H), 2.00 (quin, *J* = 6.2 Hz, 2H).

### Synthesis of 4-[4-[2-[3-[4-(2-Carboxyethyl)-2,6-dichloro-phenoxy]-propoxy]-ethoxy]-3,5-Dichloro-anilino]-Pyridine-3-Carboxylic Acid (B27) (Scheme 16)

Step 1: Compound *k8* (200 mg, 391.22 μmol, 1 eq), 4-chloropyridine-3-carboxylic acid (92.46 mg, 586.83 μmol, 1.5 equiv) were taken up into a microwave tube in ACN (5 mL). After stirring at 80 °C for 1 h under microwave, the reaction mixture was concentrated to remove solvent and afford 4-[3,5-dichloro-4-[2-[3-[2,6-dichloro-4-(3-methoxy-3-oxo-propyl)-phenoxy]-propoxy]-ethoxy]-anilino]-pyridine-3-carboxylic acid (210 mg) as a yellow solid. The crude product was used in the next step without further purification.

Step 2: to a solution of 4-[3,5-dichloro-4-[2-[3-[2,6-dichloro-4-(3-methoxy-3-oxo-propyl)-phenoxy]-propoxy]-ethoxy]-anilino]-pyridine-3-carboxylic acid (190 mg, 300.48 μmol, 1 equiv) in dioxane (5 mL)/MeOH (5 mL) /H_2_O (3 mL) was added LiOH·H_2_O (12.61 mg, 300.48 μmol, 1 equiv) at 25 °C. The reaction was stirred at 25 °C for 3 h before acidification with HCl (1M) to pH 5–6 that led to a solid formation. The suspension was filtered to give the cake then dissolved in DMSO/MeOH/THF/H_2_O (1:1:1:1, 10 mL), adjusted with NaHCO_3_ (sat., aq.) to pH 8–9. The residue was purified by prep-HPLC (column: Phenomenex Luna C18 150*30 mm*5 μm; mobile phase: [water (0.04% HCl)-ACN]; B%: 30%–55%, 10 min, to give B27 (65.1 mg, 99.2% purity)) as a white solid. LCMS: (M+H^+^): 618.9 @ 2.792 min (0–100% ACN in H_2_O, 4.5 min). ^1^H NMR: (400 MHz, DMSO-d6); δ 11.21 (br s, 1H), 8.90 (s, 1H), 8.27 (br d, *J* = 6.9 Hz, 1H), 7.66–7.53 (m, 2H), 7.36 (s, 2H),7.16–6.99 (m, 1H), 4.22–4.16 (m, 2H), 4.02 (t, *J* = 6.4 Hz, 2H), 3.82–3.74 (m, 2H), 3.69 (t, *J* = 6.2 Hz, 2H), 2.81–2.72 (m, 2H), 2.57–2.54 (m, 2H), 2.00 (quin, *J* = 6.3 Hz, 2H).

### Transthyretin Preparation

Recombinant TTR was produced by recombinant technology using a peTM11 plasmid coding for an N-terminal His6-tag and a TEV cleavage site. The plasmid was transformed into *E. coli* BL21 (DE3) cells. To generate TTR (for ligand screening), cells were grown in Luria–Bertani medium in the presence of 30 μg/mL kanamycin.

For NMR studies triple-labeled TTR was produced using a deuterated background and Ross medium containing ^15^N ammonium sulfate and ^13^C-glucose as the only sources of nitrogen and carbon, respectively. Both unlabeled and labeled TTR were expressed and purified as previously described.^23^

### Displacement of ^125^I-T4 from Isolated Wild-Type TTR

Competition of ligands with thyroxine for binding by TTR was assayed quantitatively by a previously described procedure.^22^ Briefly, a solution of 125 nM TTR in 0.1 M Tris-HCl, 0.1 M NaCl and 0.001 M EDTA buffer, pH 8.0, was incubated overnight at 4 °C in the presence of 1.07 nM ^125^I-T4 (1,200 μCi/μg, 200 μCi/ml, PerkinElmer) and increasing concentrations of inhibitor (0–5 μM) in 40 μL final volume. ^125^I-T-4bound by TTR was separated from unbound thyroxine by gel filtration chromatography using Micro Bio Spin 6 columns (Bio-Rad) previously equilibrated with the reaction buffer containing 1% w/v BSA. After counting with a Wizard2 γ counter (PerkinElmer) for 60 s, percentage binding was plotted against the logarithm of the inhibitor concentration, and IC_50_ (concentration of ligand reducing the binding of thyroxine by TTR by 50%) was determined using a four-parameter dose–response curve with GraphPad Prism 9.

### Displacement of ^125^I-T4 from TTR in Normal Human Plasma

The displacement of ^125^I-thyroxine from TTR in whole plasma, from individuals homozygous for wild-type TTR, was studied by incubating plasma with each ligand at increasing concentrations for 30 min at 37 °C, followed by incubation with ^125^I-T4 and measurement of bound ^125^I-T4 in the immunoprecipitate TTR-ligand complex. Briefly, 5 μL of plasma was incubated for 30 min at 37 °C with 1 μL of ligand at different final molar concentrations (0–250 μM), followed by a 15 min incubation with 1 μL of ^125^I-T4 (1200 μCi/μg, 200 μCi/ml, PerkinElmer) in a 1/10 dilution with 0.25 μg/mL cold thyroxine (Sigma-Aldrich) in 0.1 M Tris-HCl, 0.1 M NaCl, pH 8.0 (reaction buffer). Immunoprecipitation was carried out overnight at 4 °C with 2.5 μL of affinity-purified sheep polyclonal antihuman TTR antibody (6.26 mg/mL, The Binding Site), 4 μL of 20% w/v PEG6000 in 0.1 M Tris-HCl, 0.1 M NaCl, pH 8.0 and 10 μL Sepharose G15 beads in the same buffer. The immunoprecipitate was washed twice with the reaction buffer containing 2% PEG6000 by centrifugation (11,7000 *g* for 15 min) and then counted with a Wizard 2 γ counter (PerkinElmer) for 60 s. Percentage binding was plotted against the logarithm of the inhibitor concentration, and IC_50_ (concentration of ligand reducing the binding of thyroxine by TTR by 50%) was determined using a four-parameter dose–response curve with GraphPad Prism 9.

### Inhibition of Mechano-Enzymatic Fibrillogenesis

Recombinant V122I TTR at 0.5 mg/mL (9 μM) in PBS, pH 7.4 was preincubated (37 °C for 30 min) with an equimolar concentration of ligand (or DMSO alone) before adding trypsin (5 ng/μL). Protein fibrillogenesis was carried out in glass vials stirred at 1400 rpm (IKA magnetic stirrer), 37 °C and spectrophotometric turbidity at 400 nm was used to monitor fibril formation over time until it reached a plateau at 96 h.

Analysis of amyloid material harvested from each sample at the end of the fibrillogenesis was carried out using Thioflavin T (ThT) emission fluorescence and Congo red staining. For these analyses, samples were centrifuged 20 min at 11,600*g* and pellets were thoroughly rinsed with PBS to remove nonbound ligand, resuspended with 100 μL PBS, pH 7.4, containing 10 μM Thioflavin T (ThT) in Costar 96-well black-wall plates. Bottom fluorescence values (excitation 440 nm, emission 480 nm) were recorded using a BMG LABTECH FLUostar Omega plate reader. Each value was normalized to the ThT signal of the TTR sample without ligand.

The same experimental conditions were used to compare inhibition of V122I TTR mechano-enzymatic fibrillogenesis in the presence of 4.5, 9, 18, and 36 μM of B26 or monovalent tafamidis or AG10. A two-way Anova Tukey’s pairwise multiple comparisons test was carried out between B26 and tafamidis (or AG10) at each ligand concentration with GraphPad Prism 9.

### NMR

2D [^15^N, ^1^H] TROSY experiments were recorded at 37 °C with a 700 MHz Bruker AVANCE NEO (Bruker, Billerica, MA) on 125 mM U-[^2^H,^13^C,^15^N] recombinant wild-type TTR samples in PBS at pH 7.4 in a mixture of H_2_O/D_2_O 95/5 v/v. 2D TROSY^35^ were acquired at 0.3, 0.6, and 1 molar ratio B26/wild- type TTR. The assignment of the holo TTRs was confirmed by 3D [^13^C,^15^N,^1^H] HNCA spectra. The chemical shift perturbation of the peaks was analyzed at 1:1 ratio and calculated as

$$\Delta \delta =\sqrt{{\left(\Delta \delta_{\mathrm{HN}}\right)}^{2}+{\left(\frac{\Delta \delta_{N}}{6.5}\right)}^{2}}$$

Spectra were processed with Topspin 4.09 (Bruker, Billerica, MA) and analyzed in NMRFAM-SPARKY.^36^

### Docking

Autodock4 was used for docking mds84 and B26 to TTR.^30^ The parameters were refined for mds84, for which we have the crystal structure of the complex (pdb: 3IPE)^22^ and the same protocol was applied for the docking of B26. We reduced the number of rotatable bonds from 15 to 6 to avoid distortion of the ligand and set the size of the grid 20 Å × 30 Å × 40 Å. The box was centered in the middle of the binding channel. A population of 50 individuals was created and a Genetic Lamarkian Algorithm was used for each to search for the best position with a maximum of 2,500,000 energy evaluations. The energy was evaluated using the semiempirical Autodock4 free energy force field. The generated structures were then clustered with a maximum tolerance for the rmsd of 2 Å.

### Kinetics of Ligand Binding by Fluorescence Emission

Kinetics of binding within the first second of reaction was evaluated with an SFM 3000 stopped flow device coupled to a MOS500 spectrometer with a fluorescence detection system (Bio Logic, Claix, France) and a cell path length of 1.5 mm (dead-time <5 ms). Total fluorescence emission over 325 nm, using a cut off filter, was monitored after excitation at 280 nm at 37 °C and 1 μM recombinant wild-type TTR in the presence of 1 μM of ligand in PBS pH 7.4 at a final concentration of 0.9% DMSO. Kinetic data were acquired as the average of at least 3 experimental mixings and fitted using the following equation

y(t)=q+Aexp(−kt)

where *y*(*t*) is the observed fluorescence *q* the offset of the baseline. *A* and *k* are, respectively, amplitude and rate constant of the observed fluorescence change.

## Supplementary Material







## Notes

Movie S1 reporting the propagation effect of B26 (MP4)
Molecular Formula Strings (CSV)
Figures showing overlay of wild-type and V122I TTR structures (S1); effect of B26 on proteolysis of V122I TTR (S2) and effect electrostatics surfaces of TTR (S3); electrostatics surfaces of TTR (S2); LCMS and ^1^HNMR traces of all B-ligands (PDF)

## ††

Department of Molecular Medicine and Medical Biotechnology, University of Naples Federico II, 80131, Naples, Italy

## Abbreviations Used

ACN: acetonitrile
AcOH: acetic acid
ATTR: transthyretin amyloidosis
ATTR_v_: hereditary transthyretin amyloidosis
ATTR_wt_: wild-type transthyretin amyloidosis
bIC_50_: ligand concentration reducing binding of ^125^I-thyroxine by isolated recombinant wild-type TTR by 50%
BINAP: [1-(2-diphenylphosphanylnaphthalen-1-yl)­naphthalen-2-yl]-diphenylphosphane
BnBr: benzyl bromide
Bu_4_NI: tetrabutylammonium iodide
cLogD: computed coefficient of distribution at pH 7.4
CSP: chemical shift perturbation
DCM: dichloromethane
(DHQ)_2_PHAL: 1,4-bis­[(R)-[(2S,4S,5R)-5-ethyl-1-azabicyclo­[2.2.2]­octan-2-yl]-(6-methoxyquinolin-4-yl)­methoxy]­phthalazine
(DHQD)_2_PHAL: 1,4-bis­[(S)-[(2R,4S,5R)-5-ethyl-1-azabicyclo­[2.2.2]­octan-2-yl]-(6-methoxyquinolin-4-yl)­methoxy]­phthalazine
DIAD: diisopropyl azodicarboxylate
DIPA: diisopropylamine
DIPEA: N,N-diisopropylethylamine
DMF: dimethylformamide
DMSO: dimethyl sulfoxide
EDTA: ethylenediaminetetraacetic acid
EtOAc: ethyl acetate
HBA: hydrogen-bond acceptor count
HBD: hydrogen-bond donor count
HBPs: halogen binding pockets
HFpEF: heart failure with preserved ejection fraction
HNCA: 3D-triple resonance NMR experiment
HPLC: high-performance liquid chromatography
*i*-PrOH: isopropyl alcohol
KOAc: potassium acetate
LAH: lithium aluminum hydride
LCMS: liquid chromatography–mass spectrometry
MeSO_2_NH_2_: methanesulfonamide
2MeTHF: 2-methyltetrahydrofuran
MPLC: medium-pressure liquid chromatography
MsCl: methanesulfonyl chloride
MTBE: methyl *tert*-butyl ether
NMP: *N*-methyl-2-pyrrolidone
NMR: nuclear magnetic resonance
NMRFAMSPARKY: enhanced software for biomolecular NMR spectroscopy
PBS: phosphate-buffered saline
Pd­(dppf)­Cl_2_CH2Cl_2_: 1,1′-bis­(diphenylphosphino)­ferrocene
PE: petroleum ether
PEG: polyethylene glycol
pIC_50_: ligand concentration reducing binding of ^125^I-thyroxine by wild-type TTR by 50% in normal human plasma
PPh_3_: triphenylphosphine
PTSA: p-toluenesulfonic acid
RBP: retinol binding protein
RMSD: root-mean-square deviation
T4: thyroxine
t-AmylOH: 2-methyl-2-butanol
TBAI: tetrabutylammonium iodide
TEA: trimethylamine
TEV: *Tobacco Etch Virus* nuclear inclusion-a endopeptidase
THF: tetrahydrofuran
ThT: thioflavin T
TLC: thin-layer chromatography
t-NaOBu: sodium tert-butoxide
TosCl: 4-toluenesulfonyl chloride
TPSA: topological polar surface area
TTR: transthyretin
Xantphos: (9,9-Dimethyl-9H-xanthene-4,5-diyl)­bis­(diphenylphosphane)
