# Supplementary material for: Design and Mechanistic Analysis of a Potent Bivalent Inhibitor of Transthyretin Amyloid Fibrillogenesis
Source: J Med Chem. 2025 May 27;68(11):11543–71. doi: 10.1021/acs.jmedchem.5c00430 (PMC12169680; doi:10.1021/acs.jmedchem.5c00430)
Supplement: Supplementary file 3 [file jm5c00430_si_003.pdf]

SUPPORTING INFORMATION

Design and mechanistic analysis of a potent bivalent inhibitor of transthyretin amyloid fibrillogenesis

P. Patrizia Mangione[a,b,‡], Guglielmo Verona[a,c,‡], Cristina Cantarutti[d], Paola Nocerino[a,#], Maria Chiara Mimmi[e], Christopher J. Swain[f], Diana Canetti[c], Sofia Giorgetti[a,b], Iain Uings[g], Julian D. Gillmore[c], Graham W. Taylor[c,h], Mark B. Pepys[h], Vittorio Bellotti[a,b], Alessandra Corazza[d,i,*].

[a] Department of Molecular Medicine, University of Pavia, 27100 Pavia, Italy

[b] Research Department, Fondazione IRCSS Policlinico San Matteo, 27100 Pavia, Italy

[c] Centre for Amyloidosis, University College London, NW3 2PF London, UK

[d] Department of Medicine, University of Udine, 33100 Udine, Italy

[e] Transplant Research Area and Centre for Inherited Cardiovascular Diseases, Fondazione IRCCS Policlinico San Matteo, 27100 Pavia, Italy

[f] Cambridge MedChem Consulting, CB22 4RN Cambridge, UK

[g] GSK Medicines Research Centre, SG1 2NY Stevenage, UK

[h] Wolfson Drug Discovery Unit, University College London, NW3 2PF, UK

[i] Istituto Nazionale Biostrutture e Biosistemi, 00136 Rome, Italy

* Corresponding author: Alessandra Corazza, alessandra.corazza@uniud.it

[#] Current address: Department of Molecular Medicine and Medical Biotechnology, University of Naples Federico II, 80131, Naples, Italy

[‡] P.P.M and G.V. contributed equally to this work.

CONTENT OF SUPPLEMENTARY INFORMATION

Figure S1: Structural comparison between wild type and V122I variant TTR (page S3)

Figure S2: SDS PAGE of V122I TTR before and after 96 h aggregation in the presence of different equivalents of B26 (page S4)

Figure S3: Electrostatics surfaces of TTR (page S5)

Movie M1 legend: (page S6)

HPLC and MS analysis (LCMS) of all B-ligands (pages S7-S21)

^{1}H NMR spectra for all B-series ligands (pages S22-S29)

SUPPLEMENTARY FIGURES

Figure S1. Close structural similarity between wild type and V122I variant TTR. The superposition of structures of wild type (pdb code: 5CN3) and V122I TTR (pdb code: 1TTR) proves the close similarity of the two proteins, which can be quantified by an RMSD value of 0.801 Å calculated for all atoms.

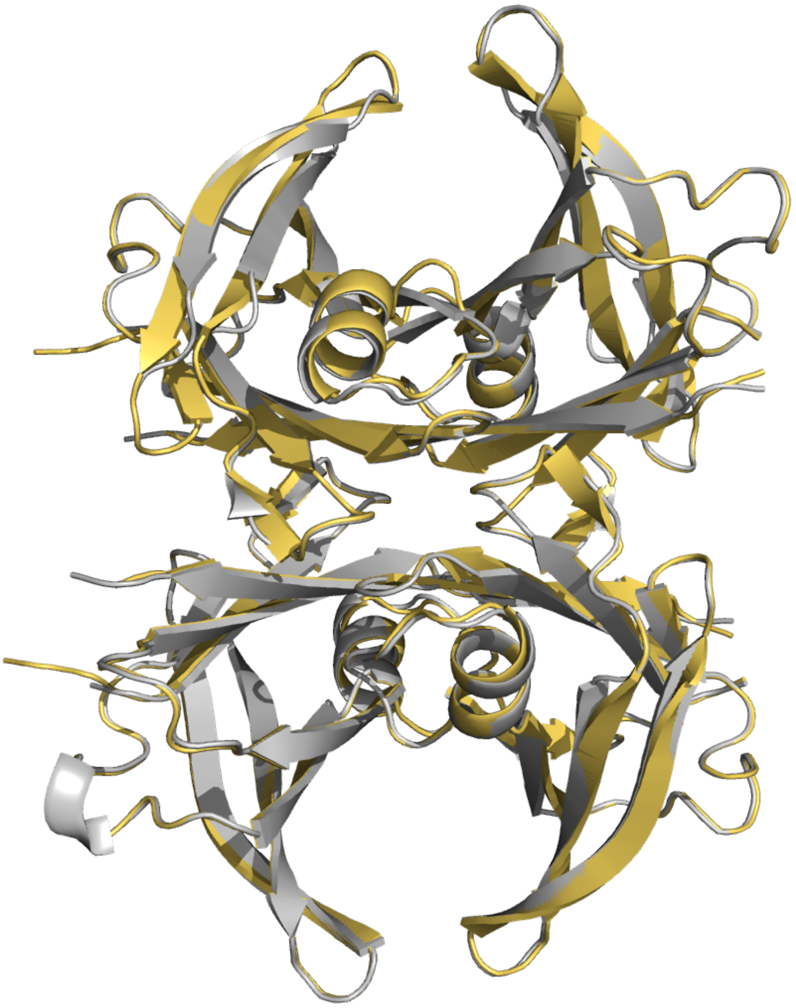

Figure S2. Effect of B26 on proteolysis of V122I TTR. Homogenous SDS-12% PAGE under reducing conditions of 9 μM V122I TTR before (a) and after 96 h aggregation in the presence of 0 (b), 4.5 μM (c), 9 (d), 18 (e) and 36 μM (f) of B26 respectively in PBS pH 7.4 at 37°C. Aggregation was carried out under fluid agitation and addition of trypsin at an enzyme: substrate ratio of 1:200. LMW marker (250 kDa, 150 kDa, 100 kDa, 75 kDa, 50 kDa, 37 kDa, 25 kDa, 20 kDa, 15 kDa, 10 kDa).

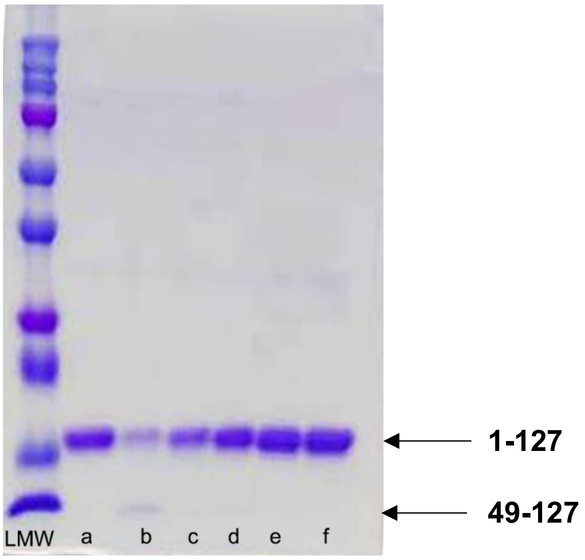

Figure S3. Electrostatics surfaces of TTR. The electrostatic potential is represented in (A) on the dimeric TTR showing the surface of the channel and the bivalent ligand B26 in stick and balls. In (B), the electrostatic potential is represented on the full tetramer, highlighting, on the surface, the positively charged region at the entrance of the binding pocket. The electrostatic potential is calculated using the APBS software[1] and the spectrum bar shows the colour grade in units of kT/e.

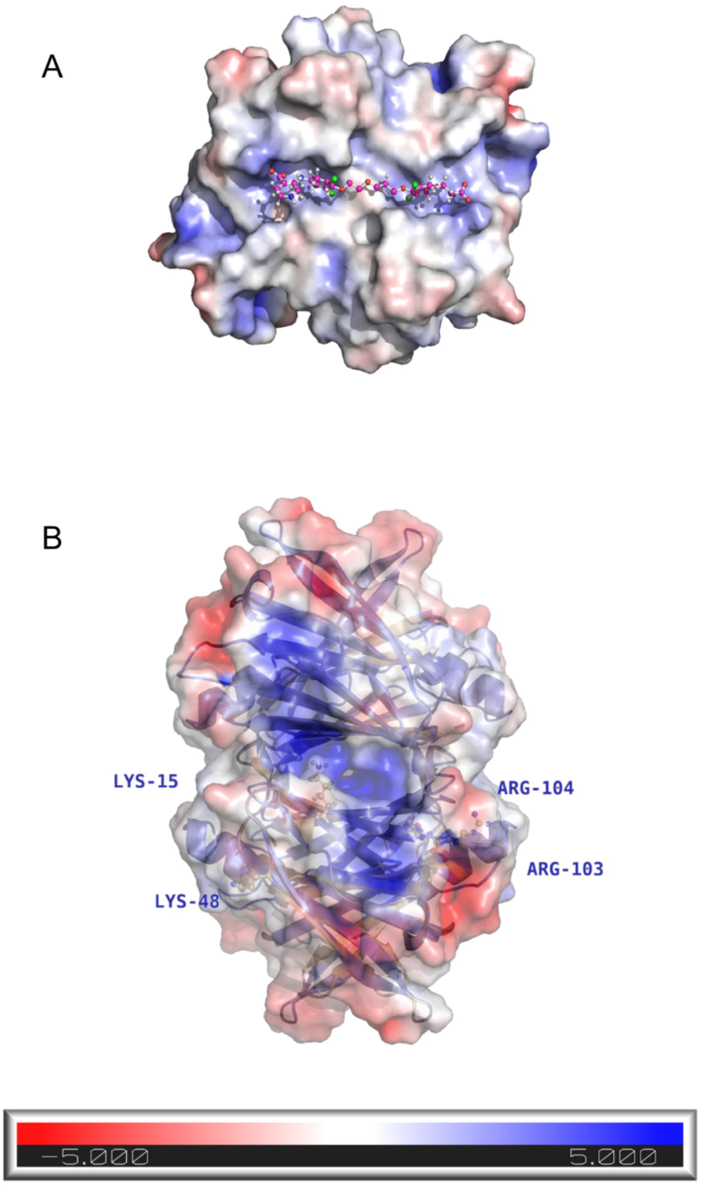

(1) Jurrus, E.; Engel, D.; Star, K.; Monson, K.; Brandi, J.; Felberg, L. E.; Brookes, D. H.; Wilson, L.; Chen, J.; Liles, K.; et al. Improvements to the APBS biomolecular solvation software suite. *Protein Sci.* **2018**, *27* (1), 112-128.

SUPPLEMENTARY MOVIE S1 LEGEND

Movie illustrating the propagation of the B26 binding effect from the HBPs to the serine protease cleavage site of TTR. TTR subunits are illustrated in green as a cartoon, while the ligand is represented in ball and stick, coloured purple (carbons), red (oxygens), blue (nitrogens), and green (chlorines) and sourced from docking results. Residues not belonging to the binding pocket (except K15) but exhibiting an above-average chemical shift are coloured in orange, depicted in ball and stick and labelled with one-letter code. The 48-49 cleavage site is depicted in ball and stick, coloured deep teal, and labelled. Hydrogen bonds connecting the binding site and the cleavage site are depicted as yellow dashed lines.

LCMS

B1

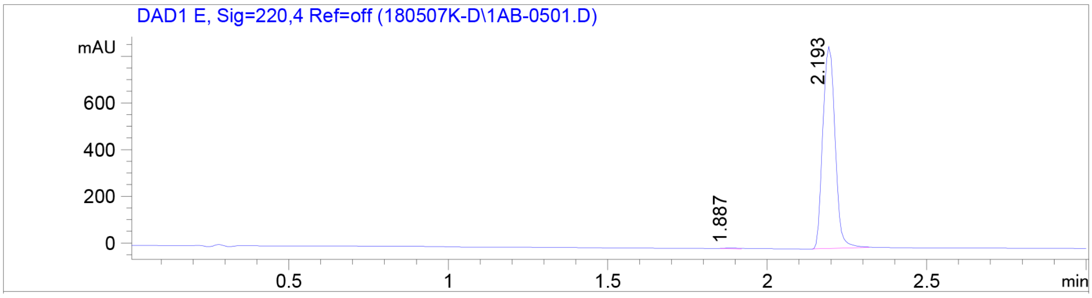

Integration Result

Signal 1 : DAD1 E, Sig=220,4 Ref=off

Peak #	RT [min]	Area	Height	Height %	Width [min]	Area %
1	1.887	10.039	3.295	0.378	0.051	0.431
2	2.193	2321.229	868.719	99.622	0.045	99.569

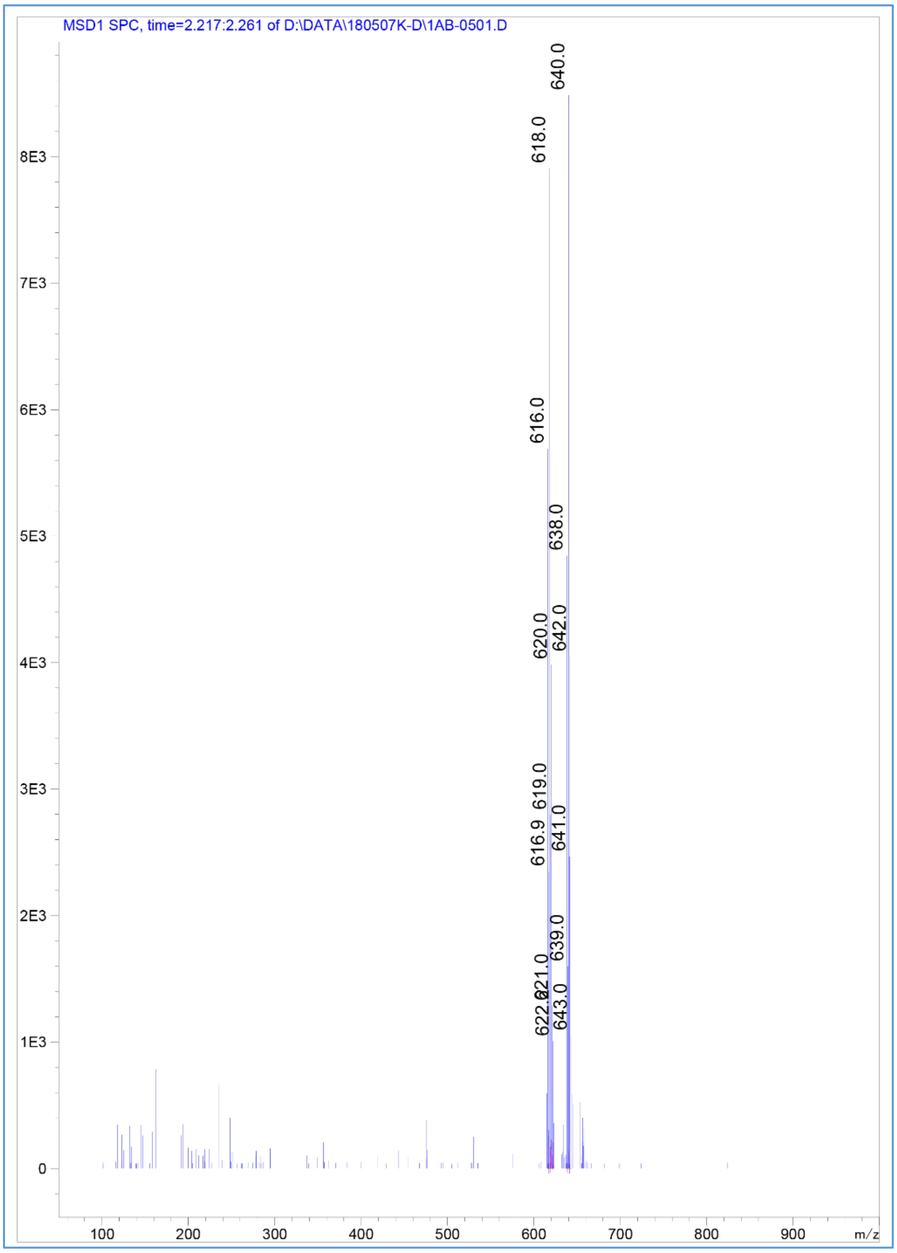

LCMS

B2

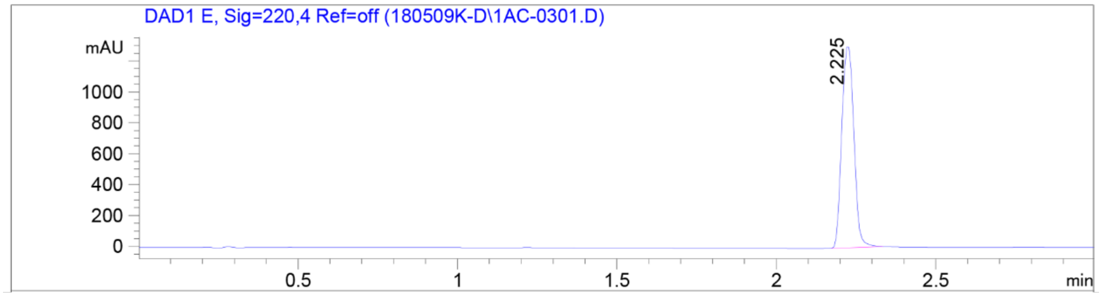


```
=====================================================================
                         Integration Result
=====================================================================
   Signal 1 : DAD1 E, Sig=220,4 Ref=off
   Peak    RT       Area     Height   Height %    Width    Area %
    #     [min]                                   [min]
   --------------------------------------------------------
     1     2.225  3450.124  1320.984   100.000     0.044   100.000
   --- --------- --------- --------- --------- --------- ---------
```

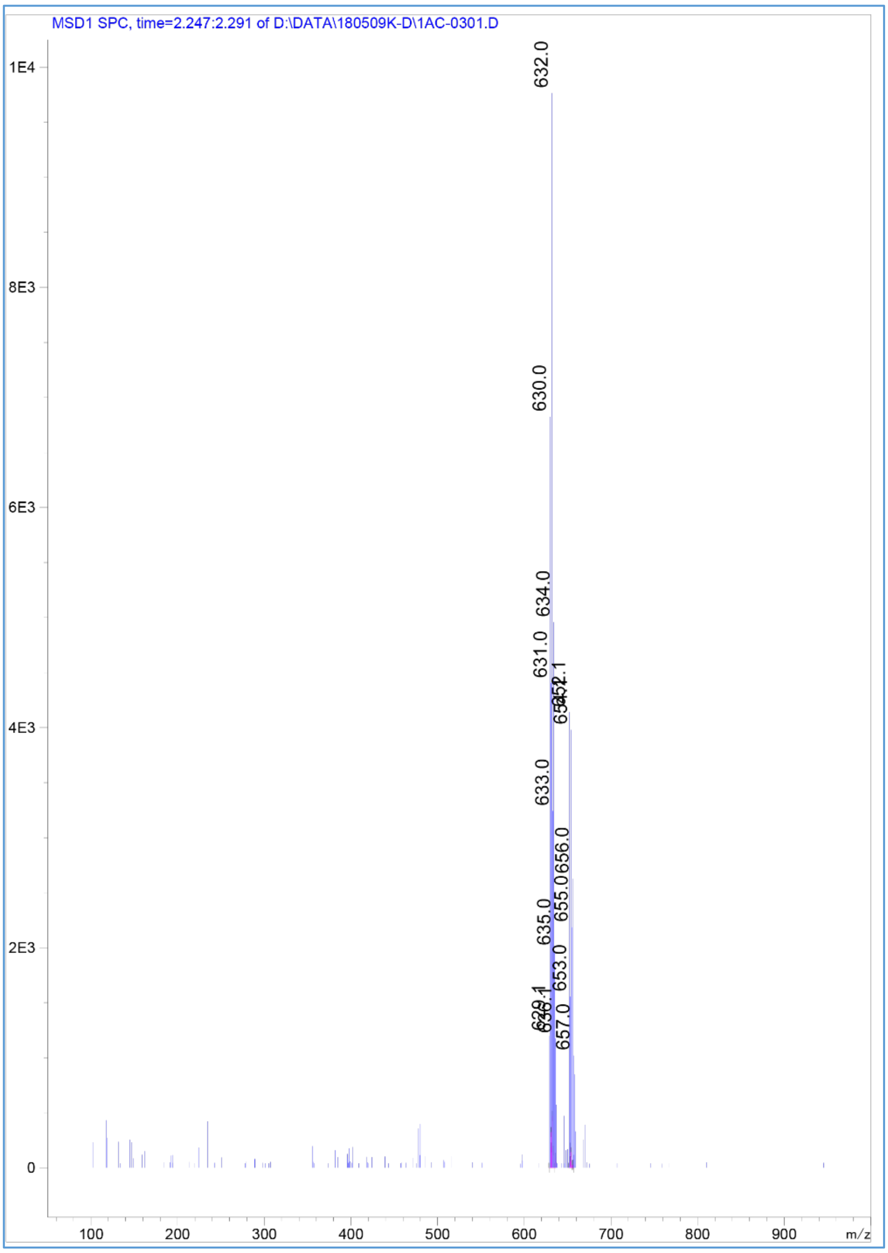

LCMS

B4

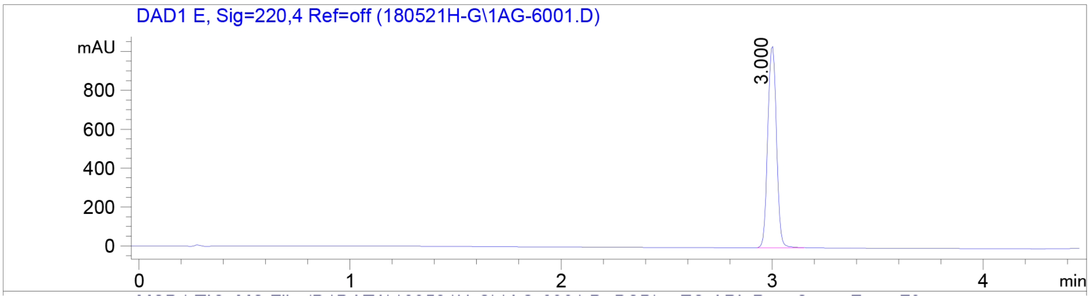

Integration Result

Signal 1 : DAD1 E, Sig=220,4 Ref=off

Peak #	RT [min]	Area	Height	Height %	Width [min]	Area %
1	3.000	2982.963	1042.537	100.000	0.045	100.000

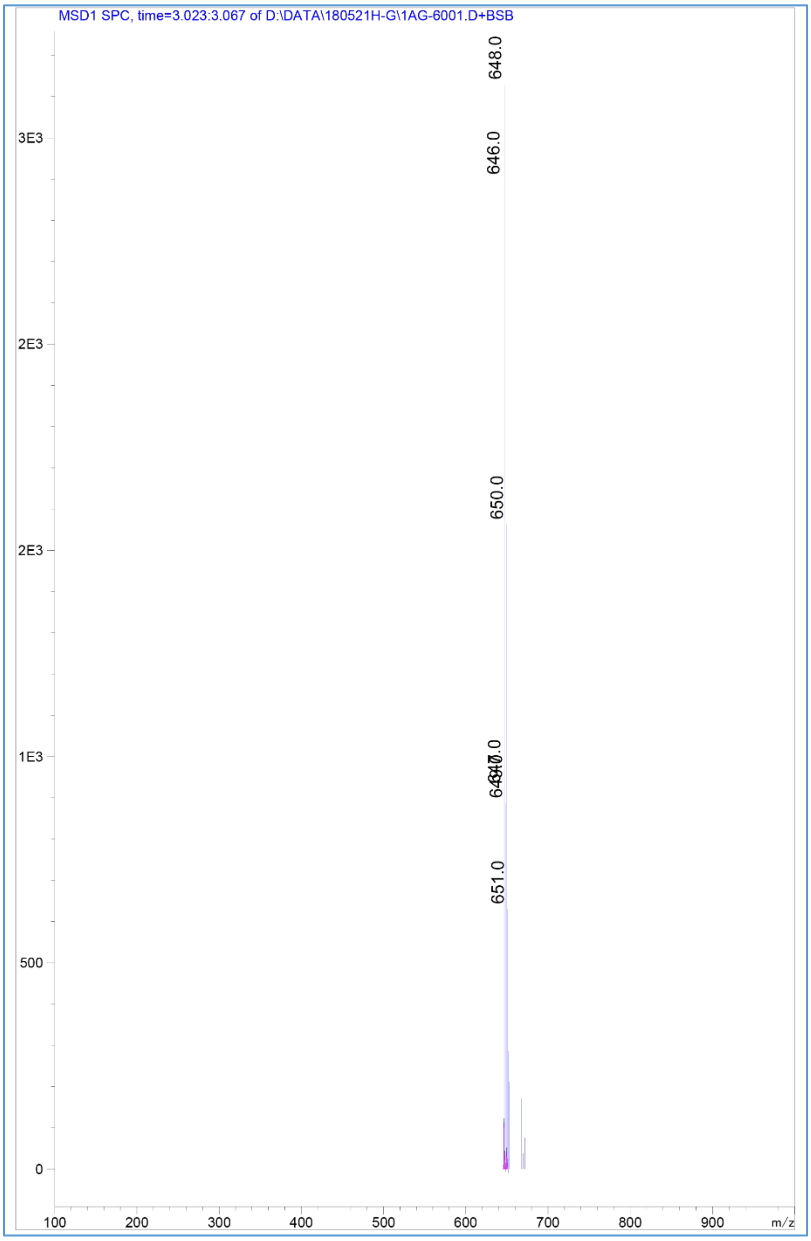

LCMS

B4A

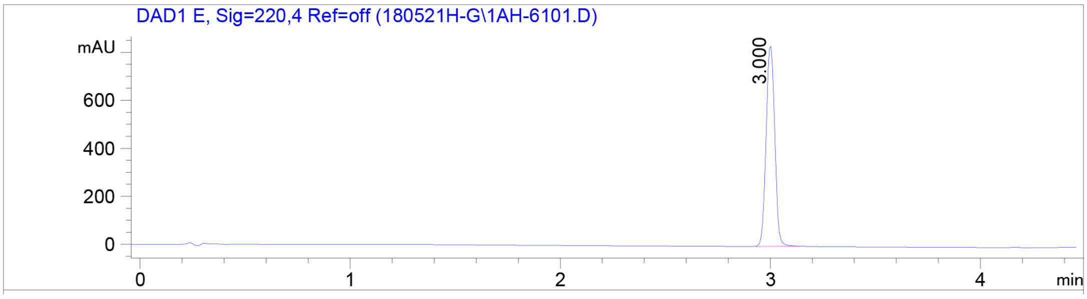

Integration Result

Signal 1 : DAD1 E, Sig=220,4 Ref=off

Peak #	RT [min]	Area	Height	Height %	Width [min]	Area %
1	3.000	2398.711	840.539	100.000	0.045	100.000

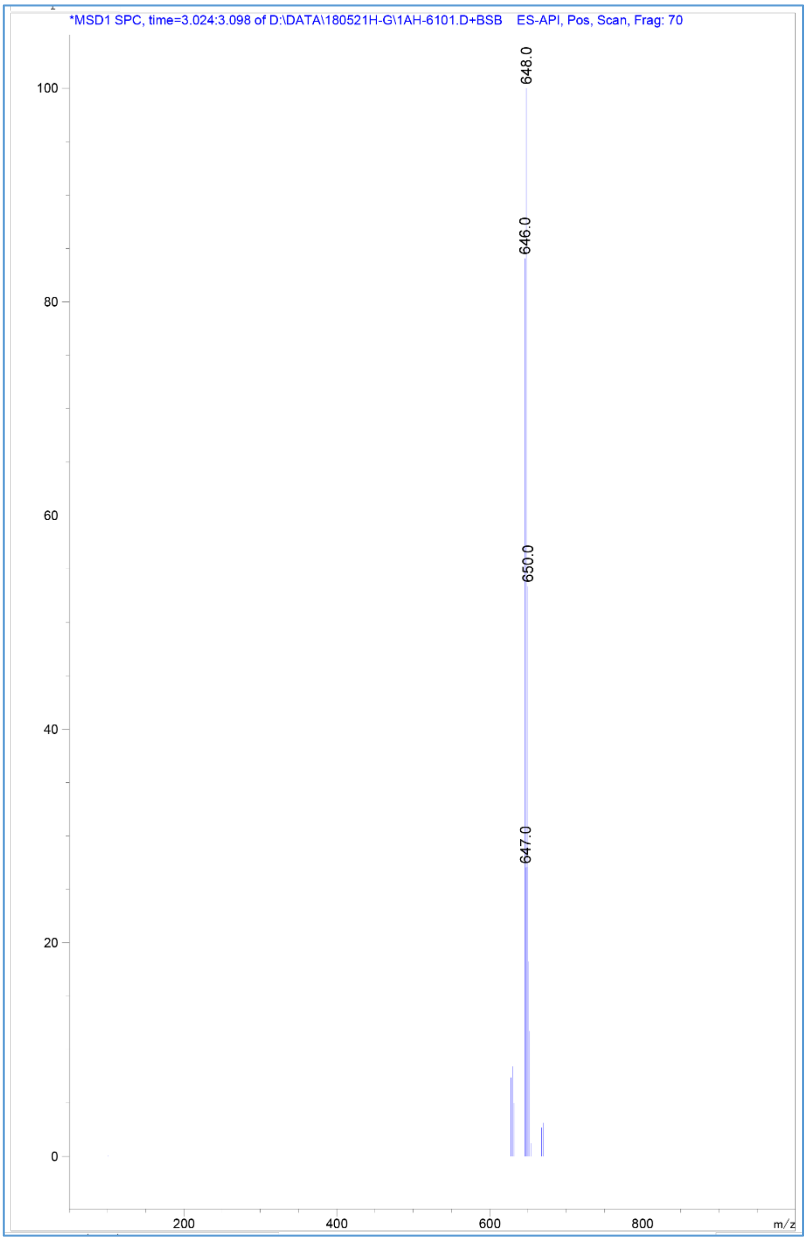

LCMS

B5

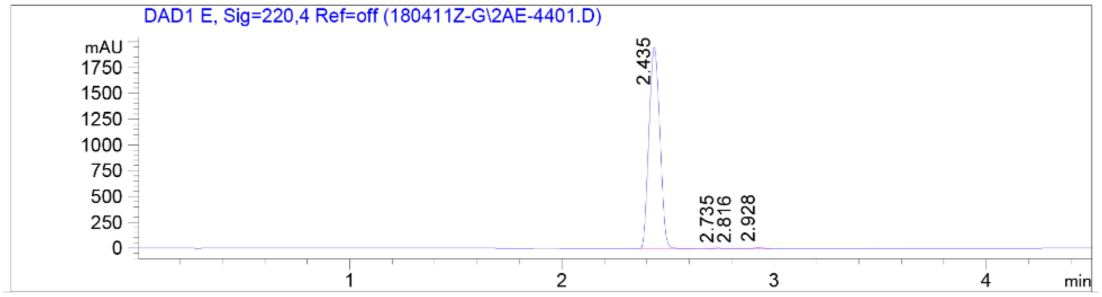

Integration Result

Signal 1 : DAD1 E, Sig=220,4 Ref=off

Peak #	RT [min]	Area	Height	Height %	Width [min]	Area %
1	2.435	7038.502	1963.202	98.924	0.060	99.195
2	2.735	15.165	5.562	0.280	0.044	0.214
3	2.816	4.763	1.769	0.089	0.043	0.067
4	2.928	37.184	14.027	0.707	0.043	0.524

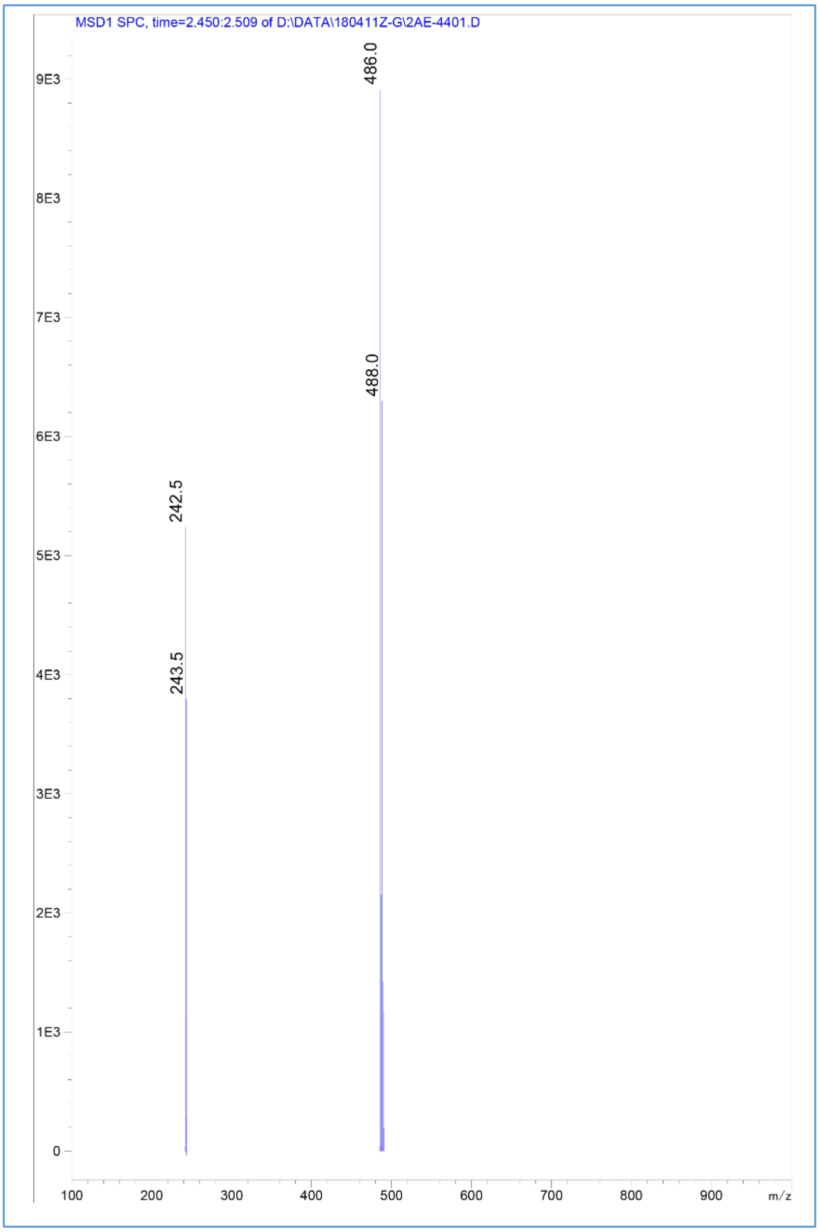

LCMS

B6

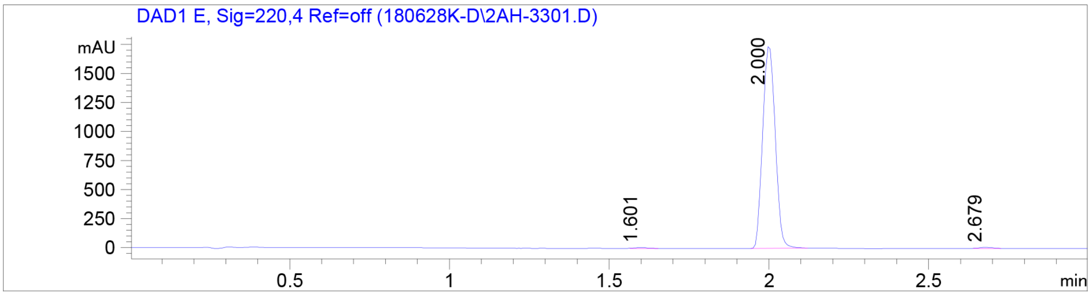

Integration Result

Signal 1 : DAD1 E, Sig=220,4 Ref=off

Peak #	RT [min]	Area	Height	Height %	Width [min]	Area %
1	1.601	16.635	6.717	0.377	0.041	0.344
2	2.000	4785.792	1764.082	99.061	0.045	99.084
3	2.679	27.624	10.011	0.562	0.046	0.572

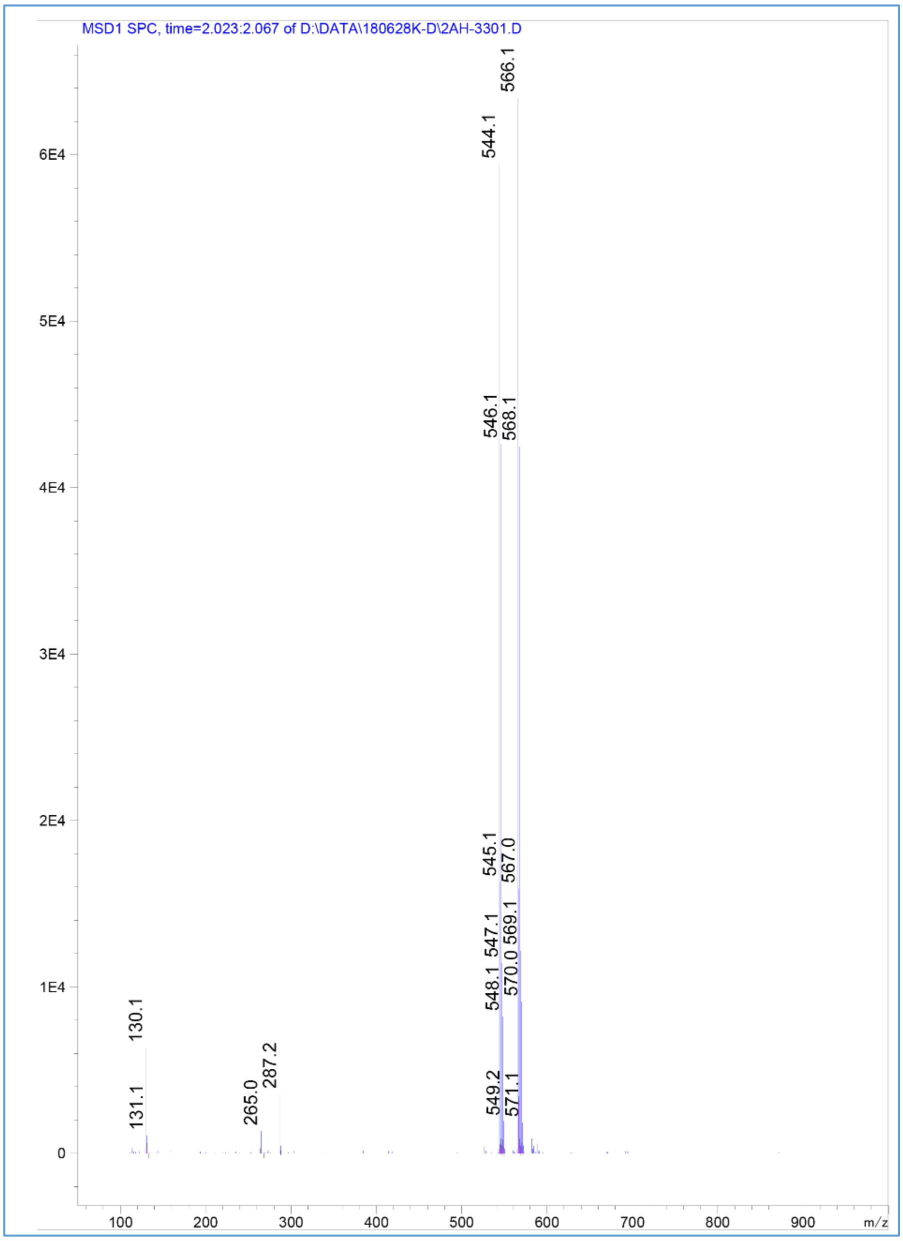

LCMS

B8

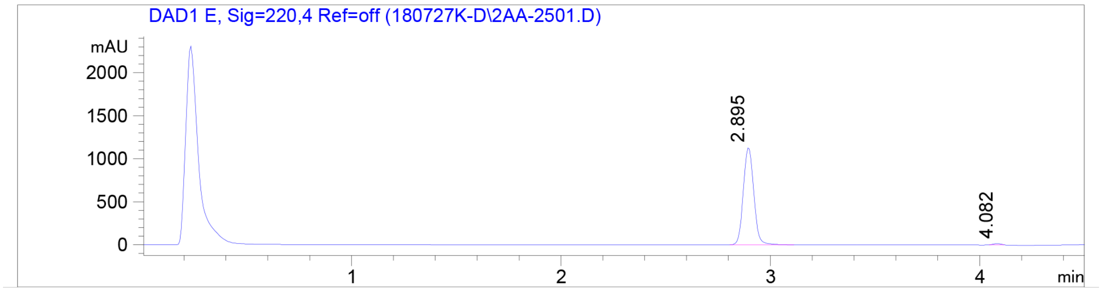

Integration Result

Signal 1 : DAD1 E, Sig=220,4 Ref=off

Peak #	RT [min]	Area	Height	Height %	Width [min]	Area %
1	2.895	3926.708	1134.522	98.824	0.054	99.160
2	4.082	33.276	13.506	1.176	0.041	0.840

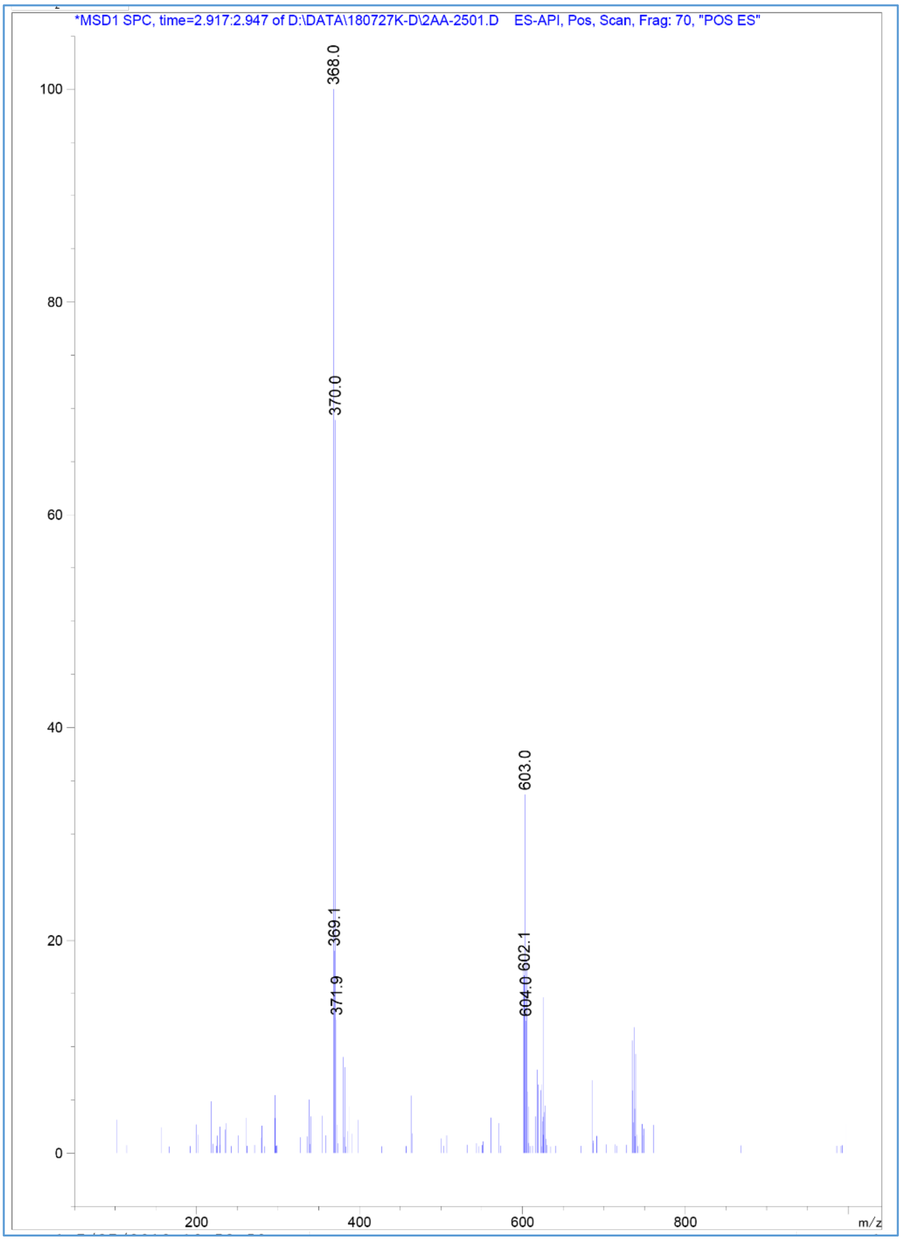

LCMS

B14

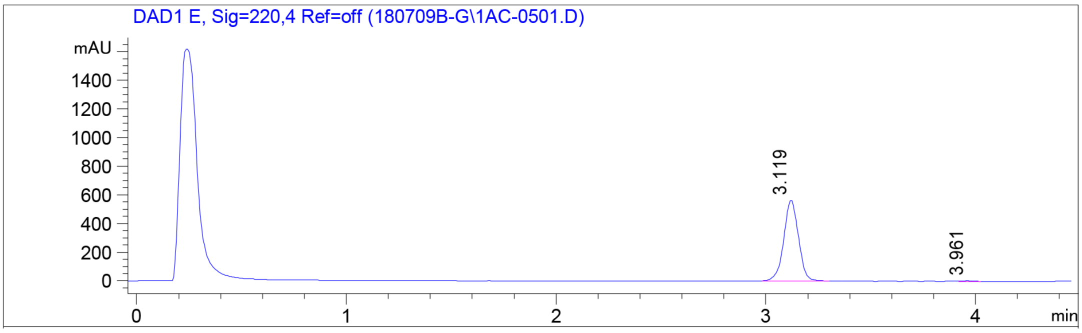

Integration Result

Signal 1 : DAD1 E, Sig=220,4 Ref=off

Peak #	RT [min]	Area	Height	Height %	Width [min]	Area %
1	3.119	2762.162	565.084	99.163	0.075	99.376
2	3.961	17.351	4.769	0.837	0.061	0.624

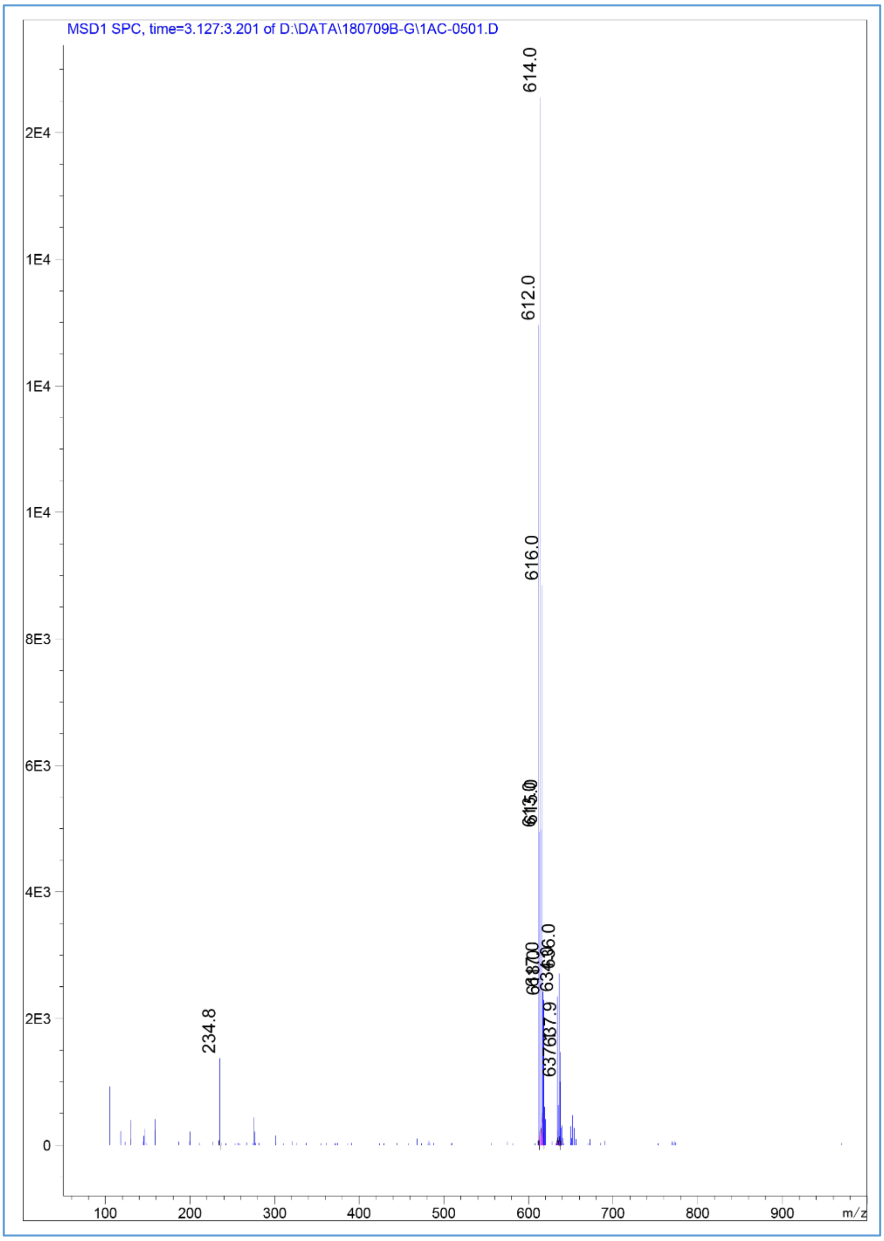

LCMS

B20

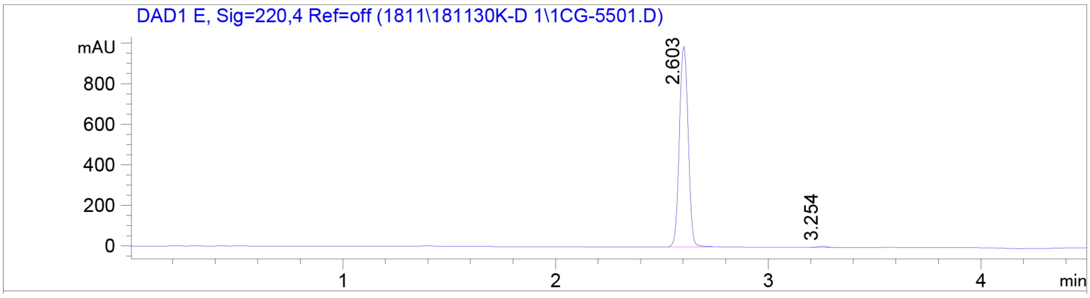


```
==========================================================================
                          Integration Result
==========================================================================
   Signal 1 : DAD1 E, Sig=220,4 Ref=off
   Peak    RT       Area     Height  Height %    Width    Area %
    #     [min]                                  [min]
   ----------------------------------------------------------
     1     2.603 2844.042   993.635    99.394    0.045    99.392
     2     3.254   17.389     6.059     0.606    0.045     0.608
   --- -------- -------- -------- -------- -------- --------
```

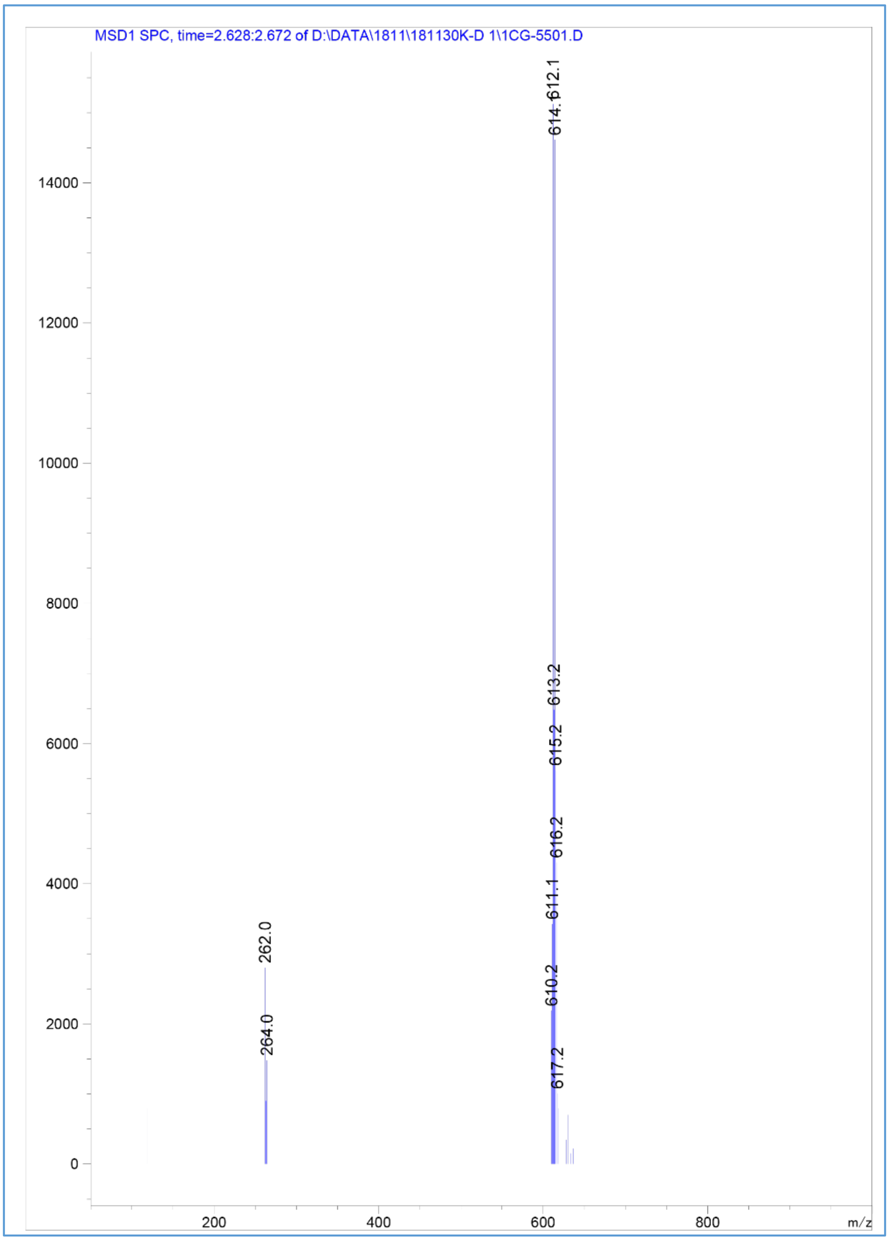

LCMS

B21

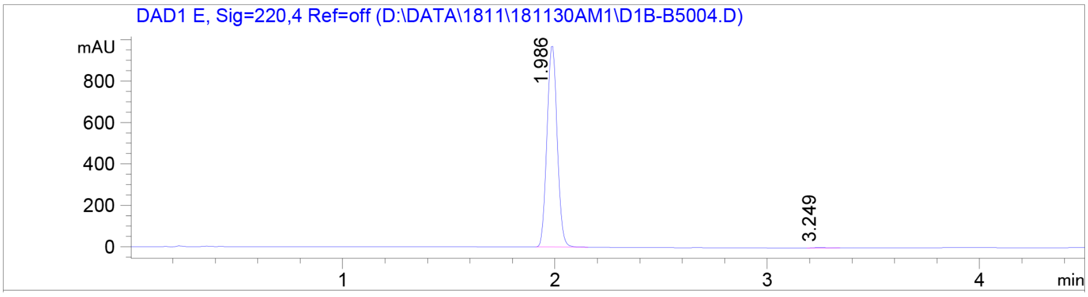

Integration Result

Signal 1 : DAD1 E, Sig=220,4 Ref=off

Peak #	RT [min]	Area	Height	Height %	Width [min]	Area %
1	1.986	3257.076	978.864	99.714	0.051	99.733
2	3.249	8.709	2.810	0.286	0.050	0.267

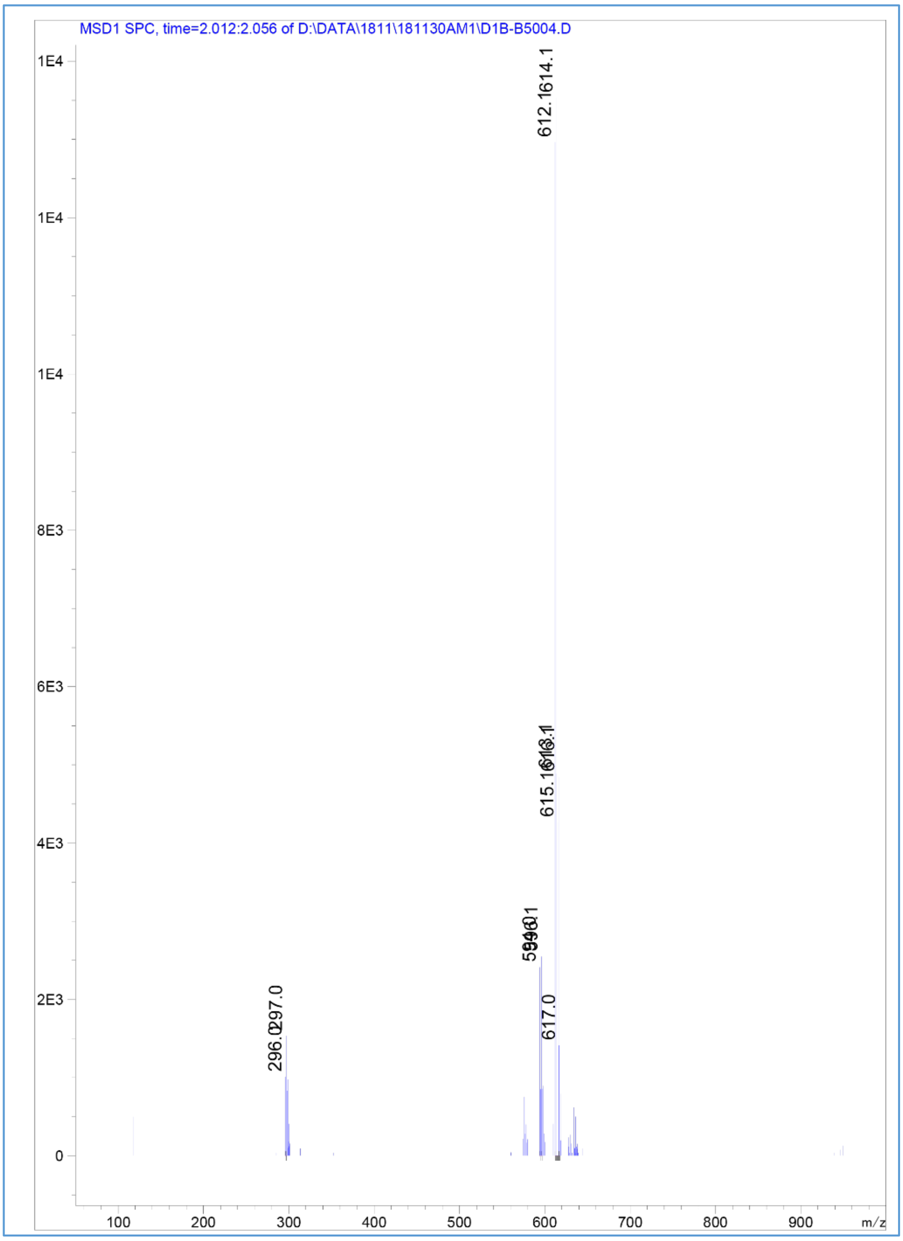

LCMS

B22

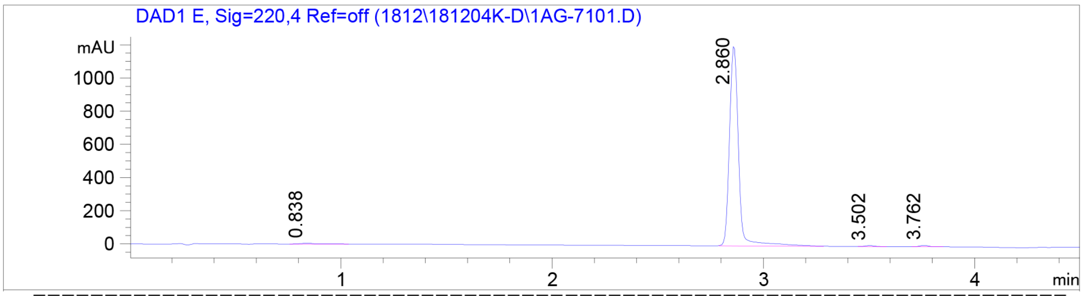

Integration Result

Signal 1 : DAD1 E, Sig=220,4 Ref=off

Peak #	RT [min]	Area	Height	Height %	Width [min]	Area %
1	0.838	22.257	5.079	0.413	0.065	0.600
2	2.860	3648.035	1210.809	98.463	0.047	98.359
3	3.502	15.487	5.662	0.460	0.044	0.418
4	3.762	23.123	8.156	0.663	0.045	0.623

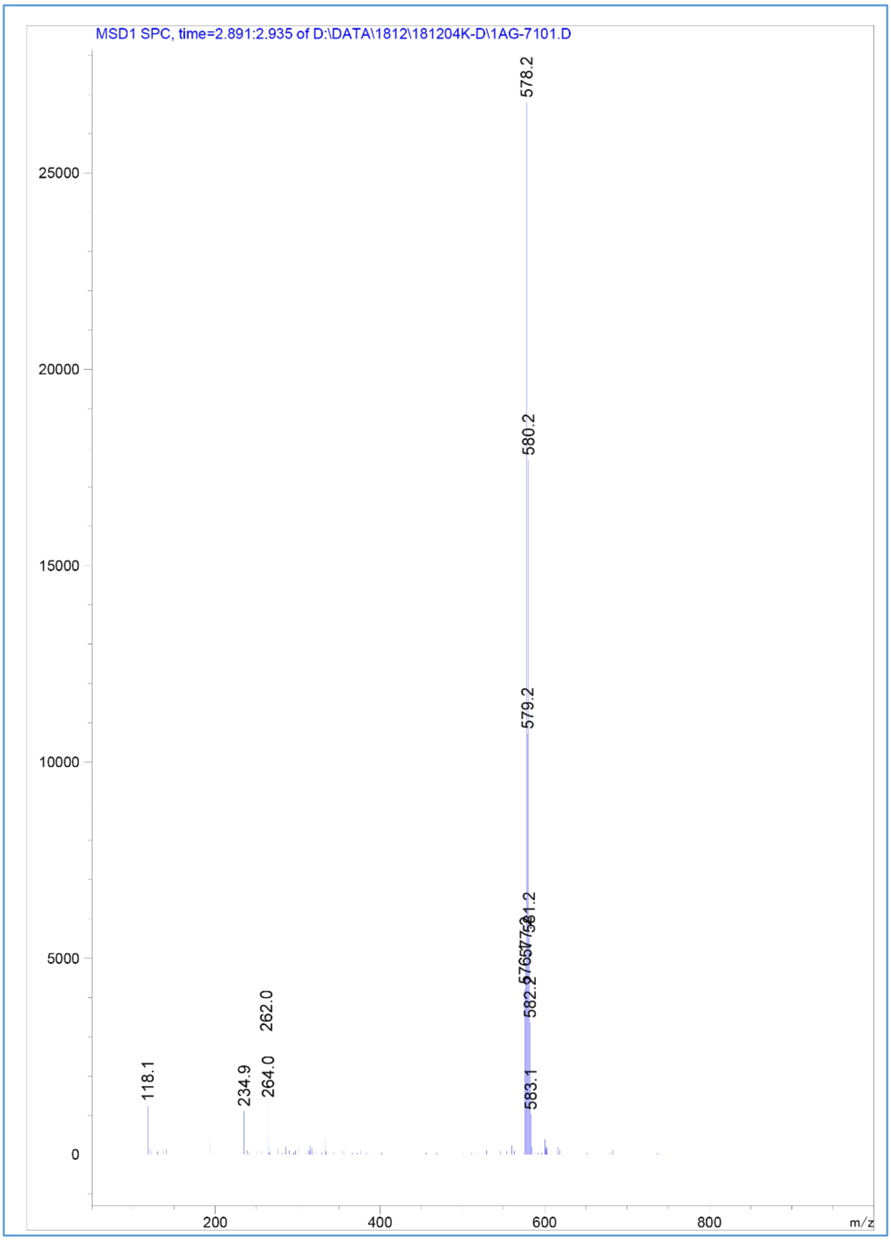

LCMS

B24

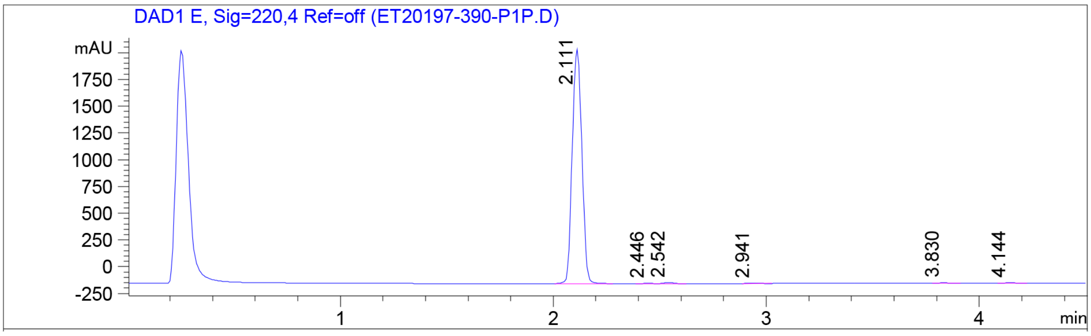

Integration Result

Signal 1 : DAD1 E, Sig=220,4 Ref=off

Peak #	RT [min]	Area	Height	Height %	Width [min]	Area %
1	2.111	6851.588	2194.351	99.009	0.050	99.058
2	2.446	8.174	2.884	0.130	0.045	0.118
3	2.542	27.871	9.480	0.428	0.046	0.403
4	2.941	7.715	2.557	0.115	0.047	0.112
5	3.830	10.967	3.758	0.170	0.046	0.159
6	4.144	10.442	3.280	0.148	0.049	0.151

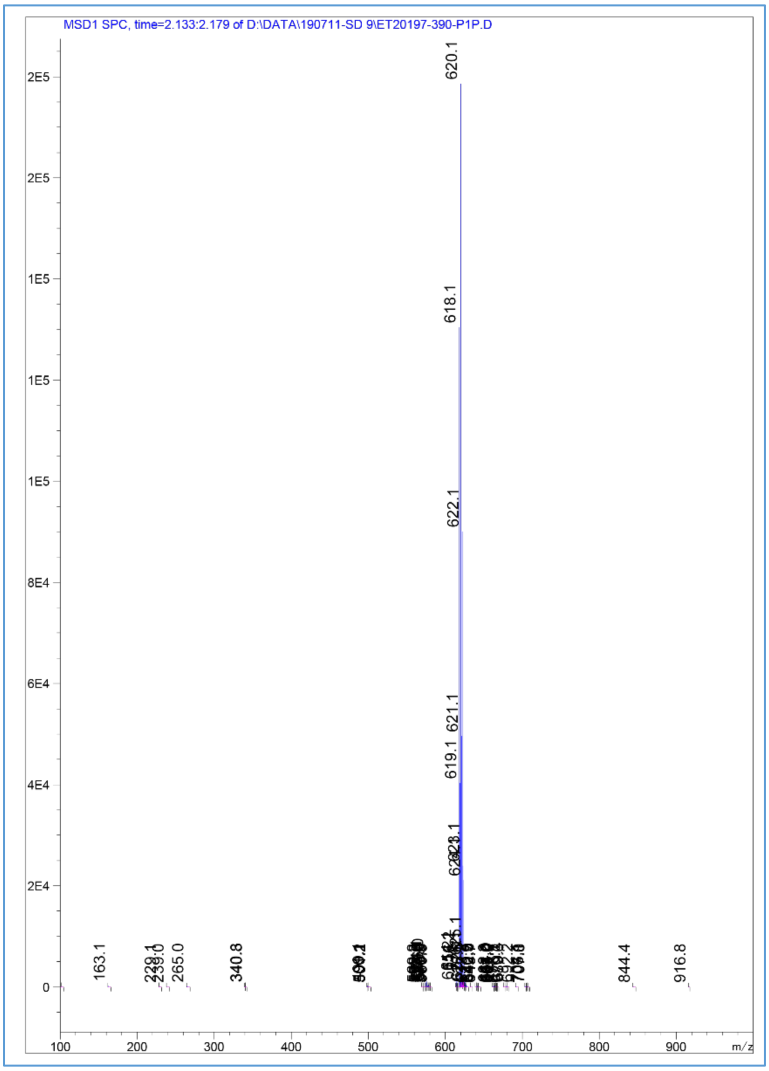

LCMS

B25

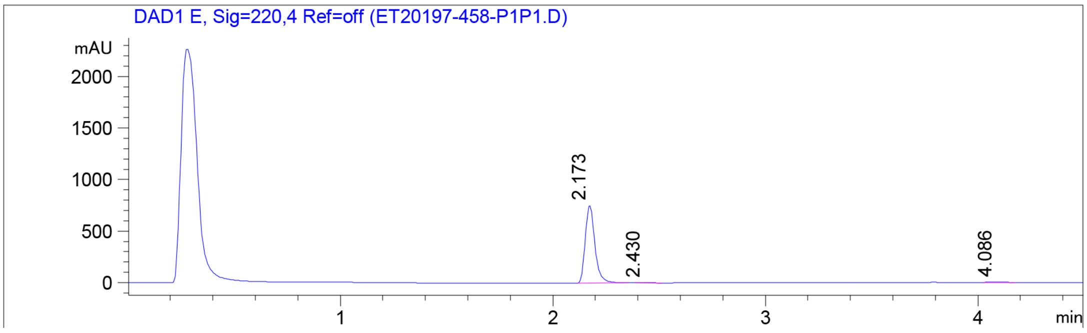


```
==========================================================================
                          Integration Result
==========================================================================
   Signal 1 : DAD1 E, Sig=220,4 Ref=off
```

Peak #	RT [min]	Area	Height	Height %	Width [min]	Area %
1	2.173	2407.067	756.382	98.967	0.049	98.885
2	2.430	6.178	2.388	0.312	0.042	0.254
3	4.086	20.952	5.510	0.721	0.056	0.861

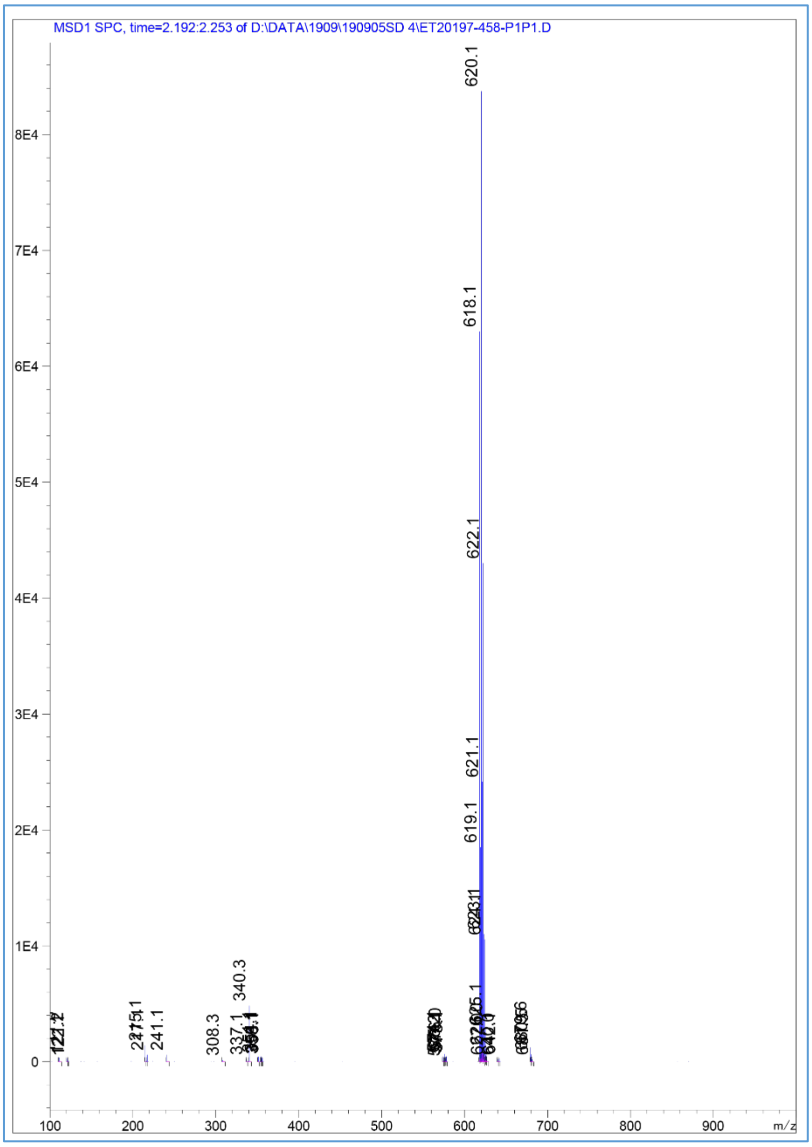

LCMS

B26

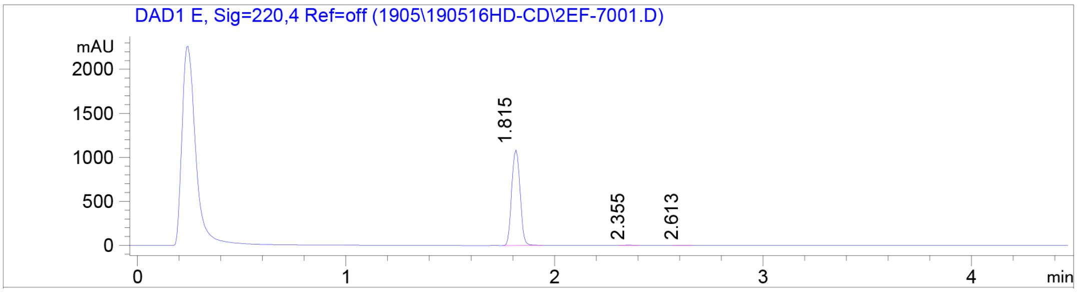

Integration Result

Signal 1 : DAD1 E, Sig=220,4 Ref=off

Peak #	RT [min]	Area	Height	Height %	Width [min]	Area %
1	1.815	3017.367	1091.346	99.507	0.046	99.475
2	2.355	12.036	4.096	0.373	0.049	0.397
3	2.613	3.890	1.316	0.120	0.049	0.128

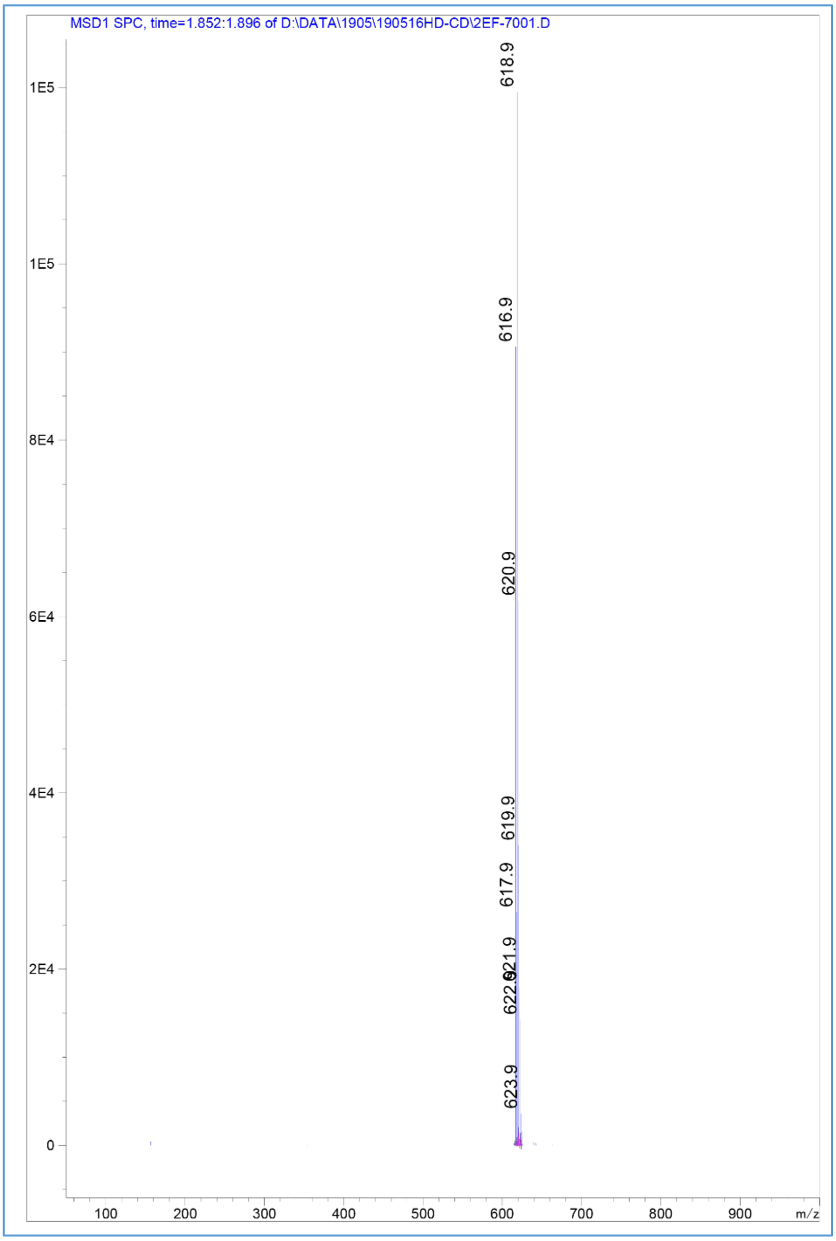

LCMS

B27

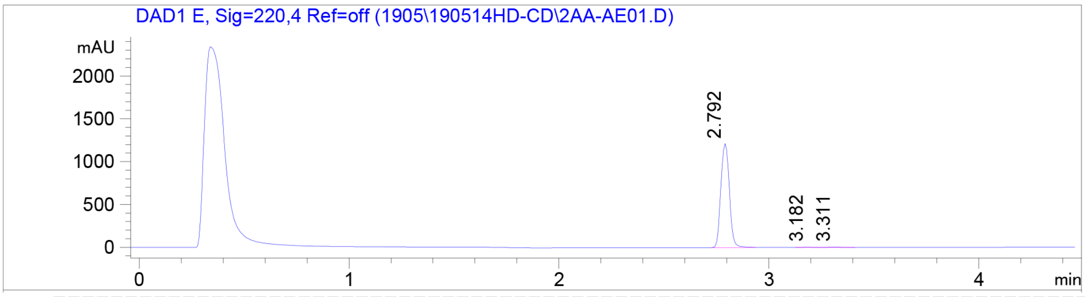

Integration Result

Signal 1 : DAD1 E, Sig=220,4 Ref=off

Peak #	RT [min]	Area	Height	Height %	Width [min]	Area %
1	2.792	3396.943	1210.844	99.318	0.045	99.262
2	3.182	9.055	3.248	0.266	0.045	0.265
3	3.311	16.197	5.071	0.416	0.049	0.473

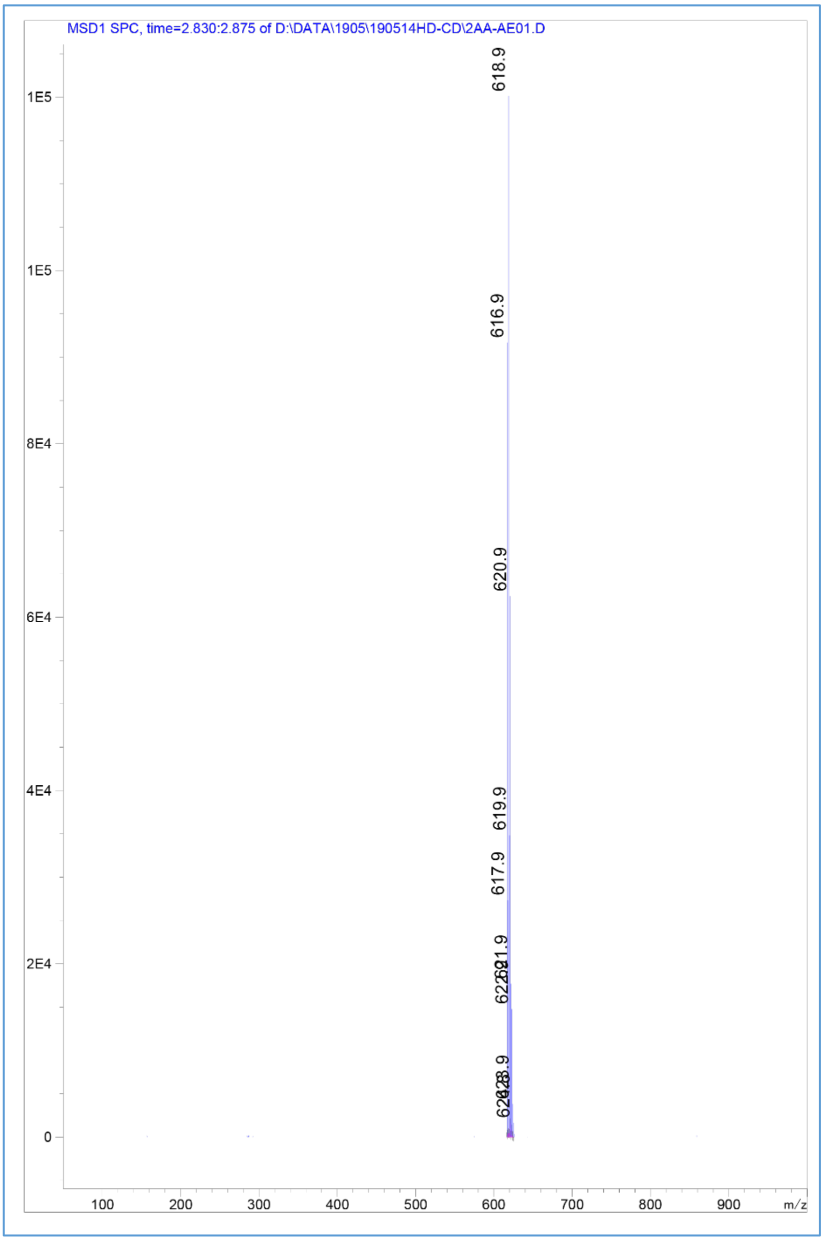

^{1}H NMR spectra

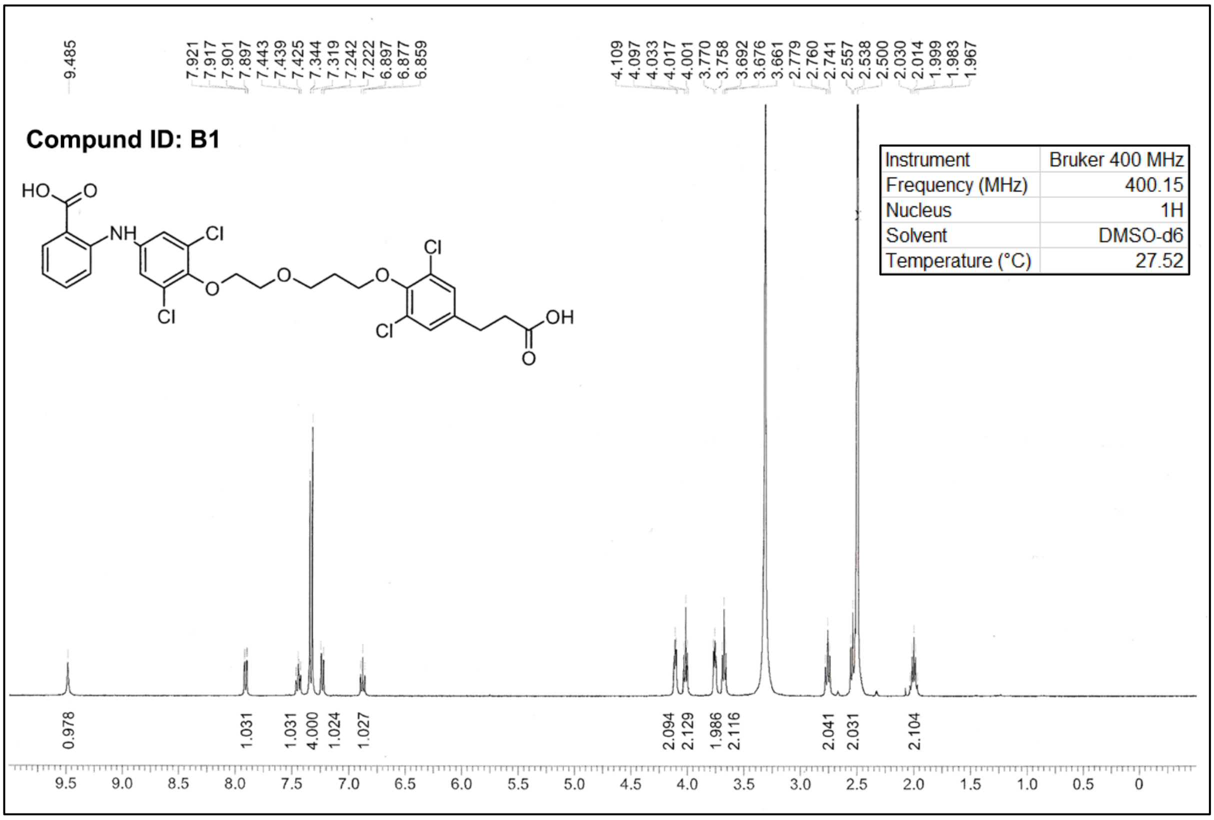

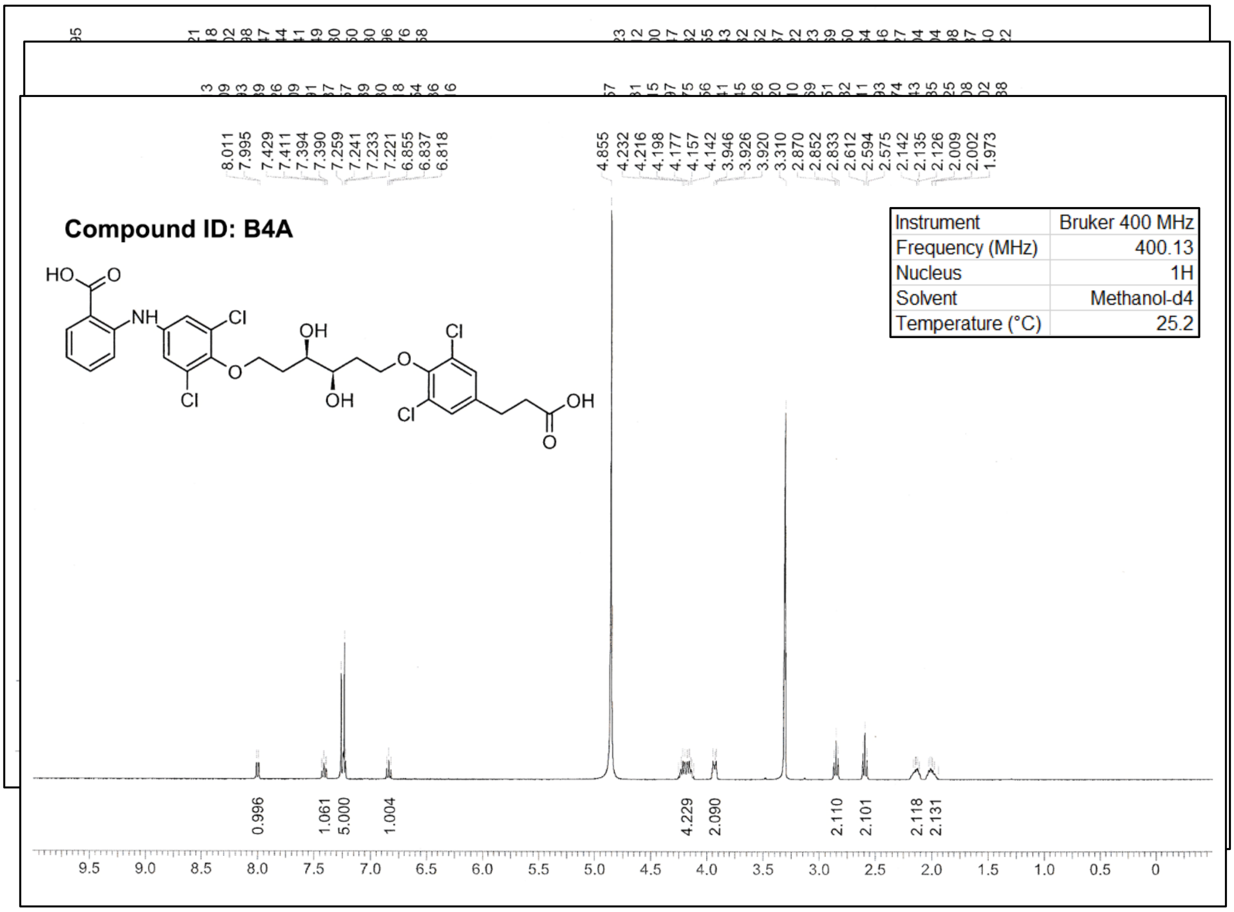

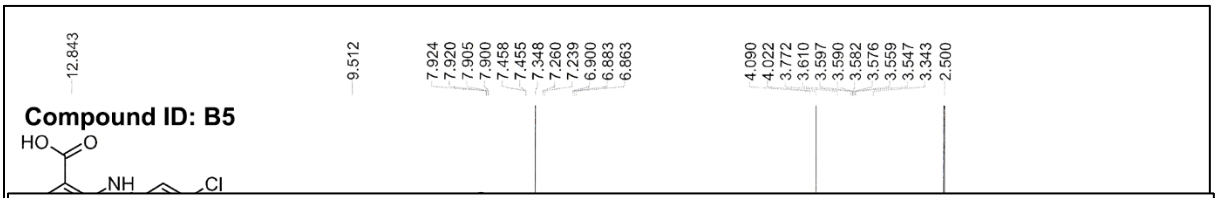
Compound ID: B5

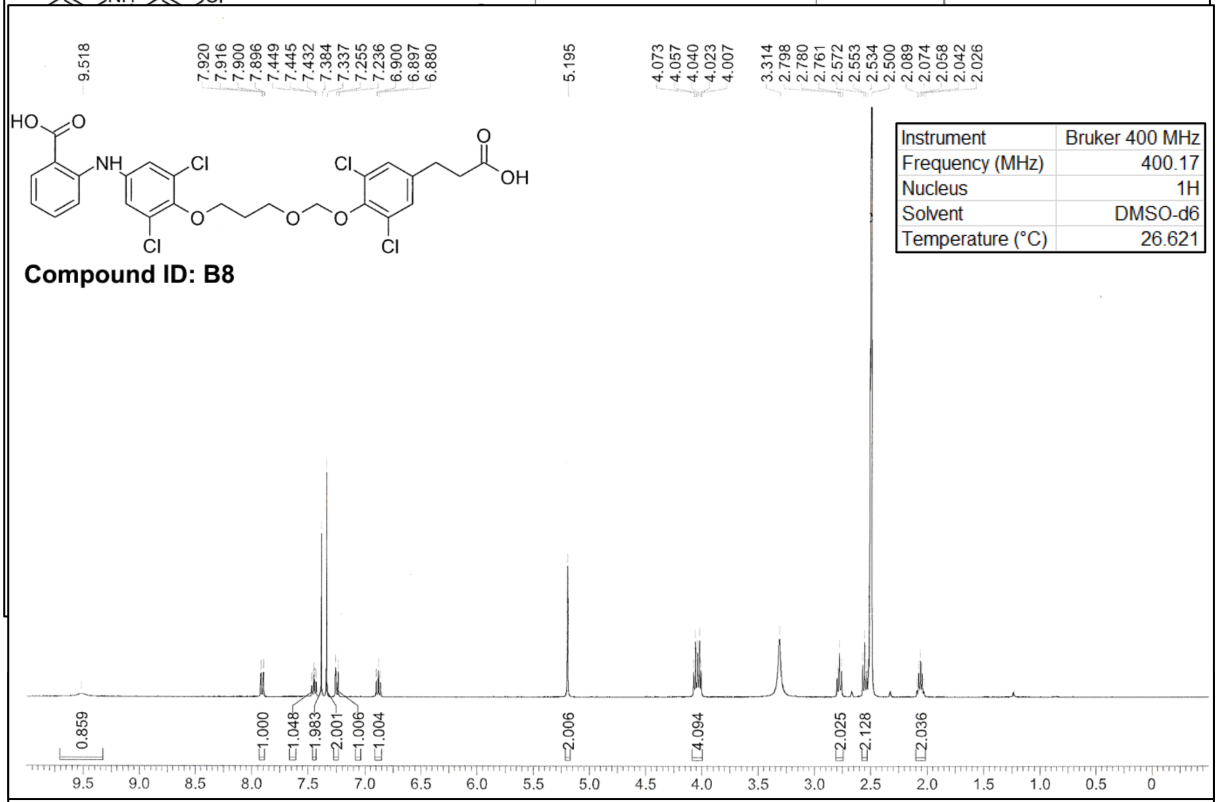
Compound ID: B8
Instrument | Bruker 400 MHz
Frequency (MHz) | 400.17
Nucleus | 1H
Solvent | DMSO-d6
Temperature (°C) | 26.621

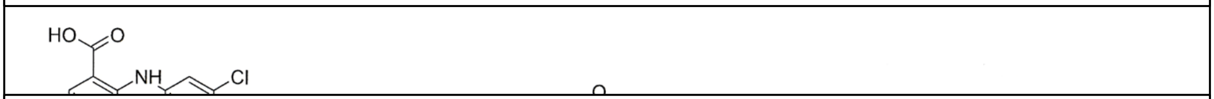

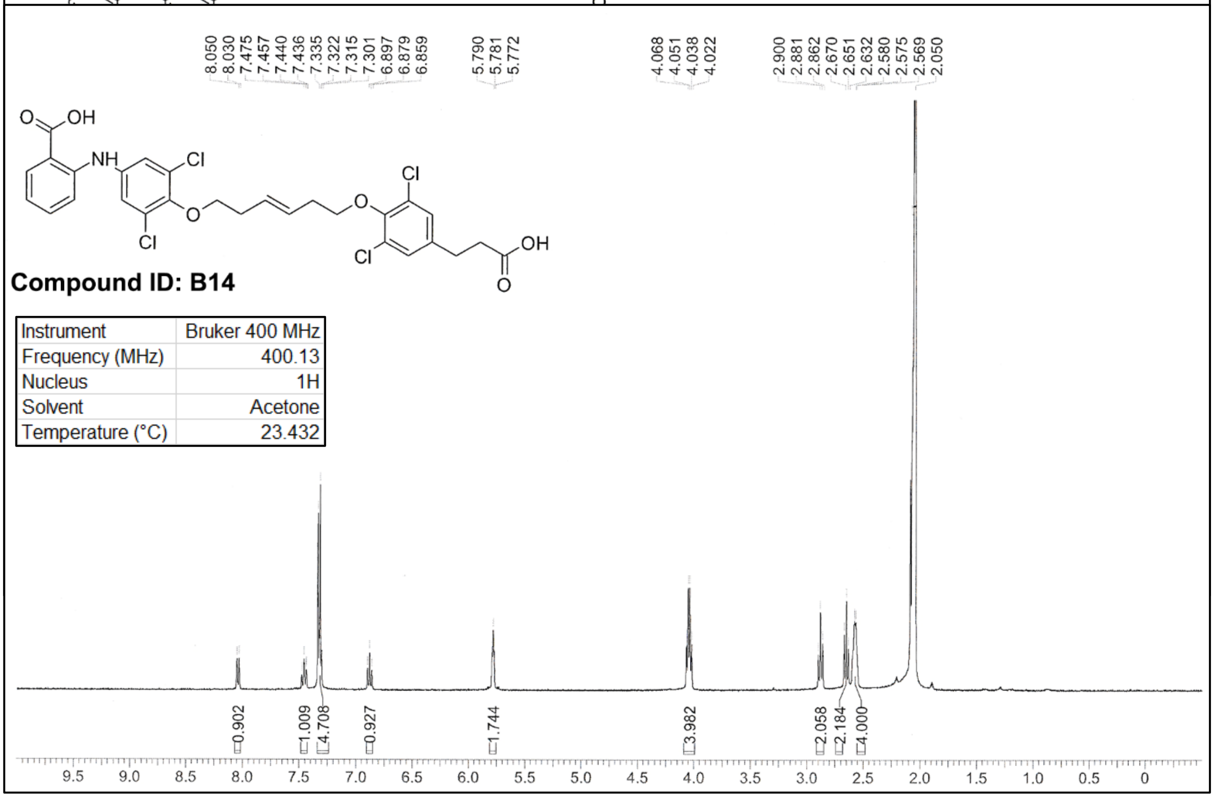
Compound ID: B14
Instrument | Bruker 400 MHz
Frequency (MHz) | 400.13
Nucleus | 1H
Solvent | Acetone
Temperature (°C) | 23.432

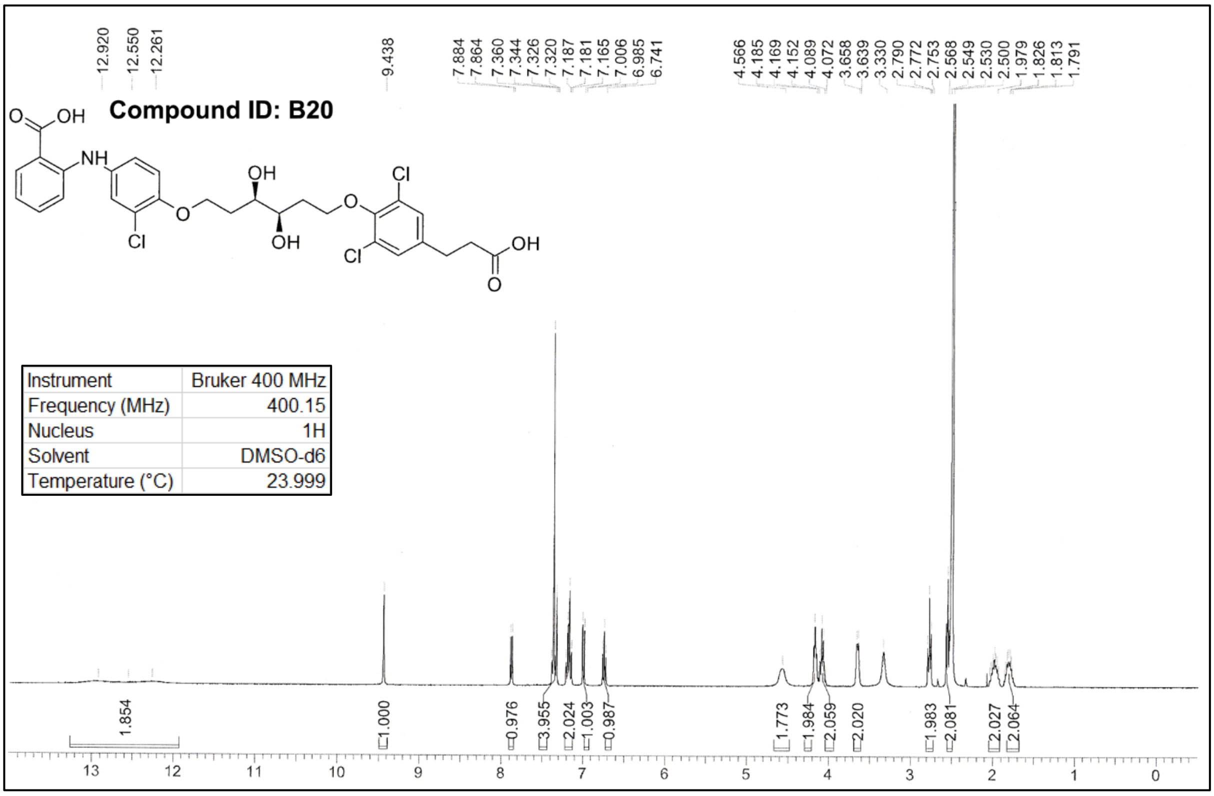
Compound ID: B20
Instrument Bruker 400 MHz
Frequency (MHz) 400.15
Nucleus 1H
Solvent DMSO-d6
Temperature (°C) 23.999

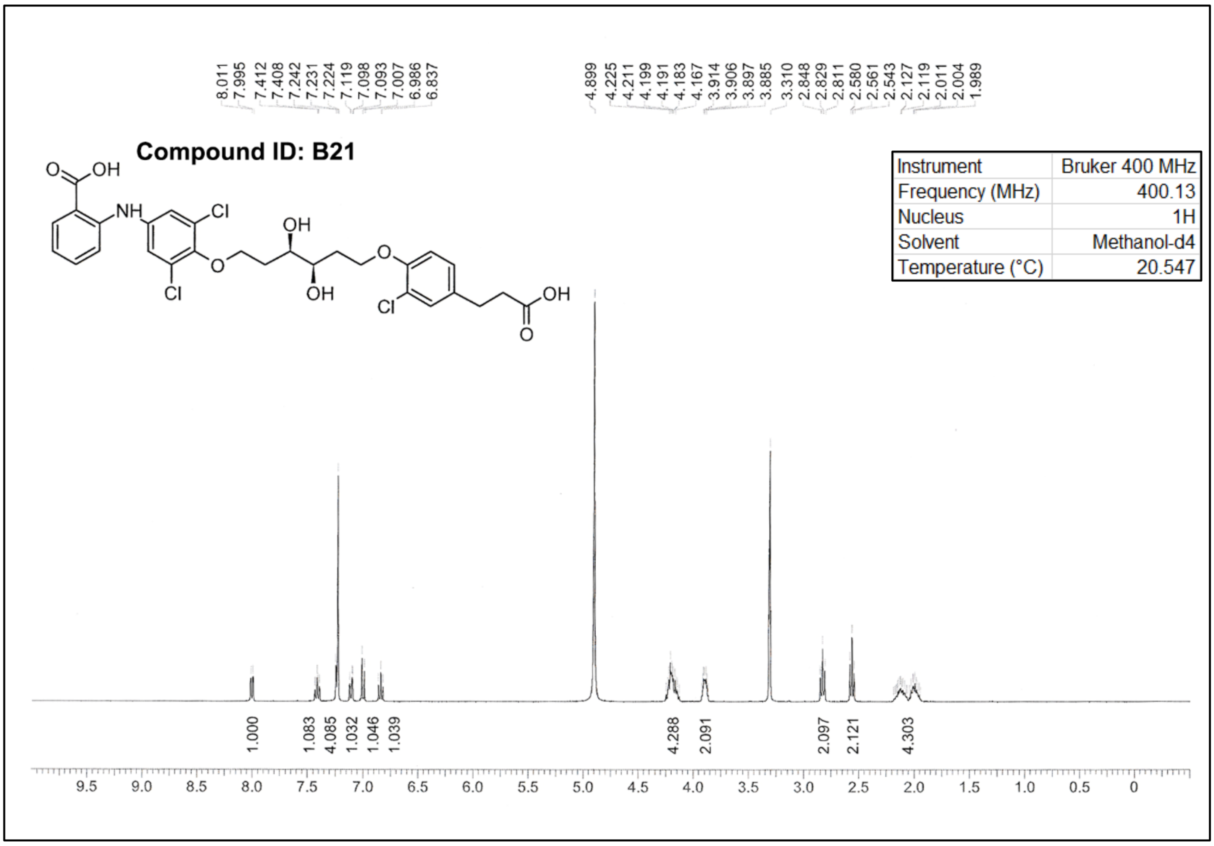
Compound ID: B21
Instrument Bruker 400 MHz
Frequency (MHz) 400.13
Nucleus 1H
Solvent Methanol-d4
Temperature (°C) 20.547

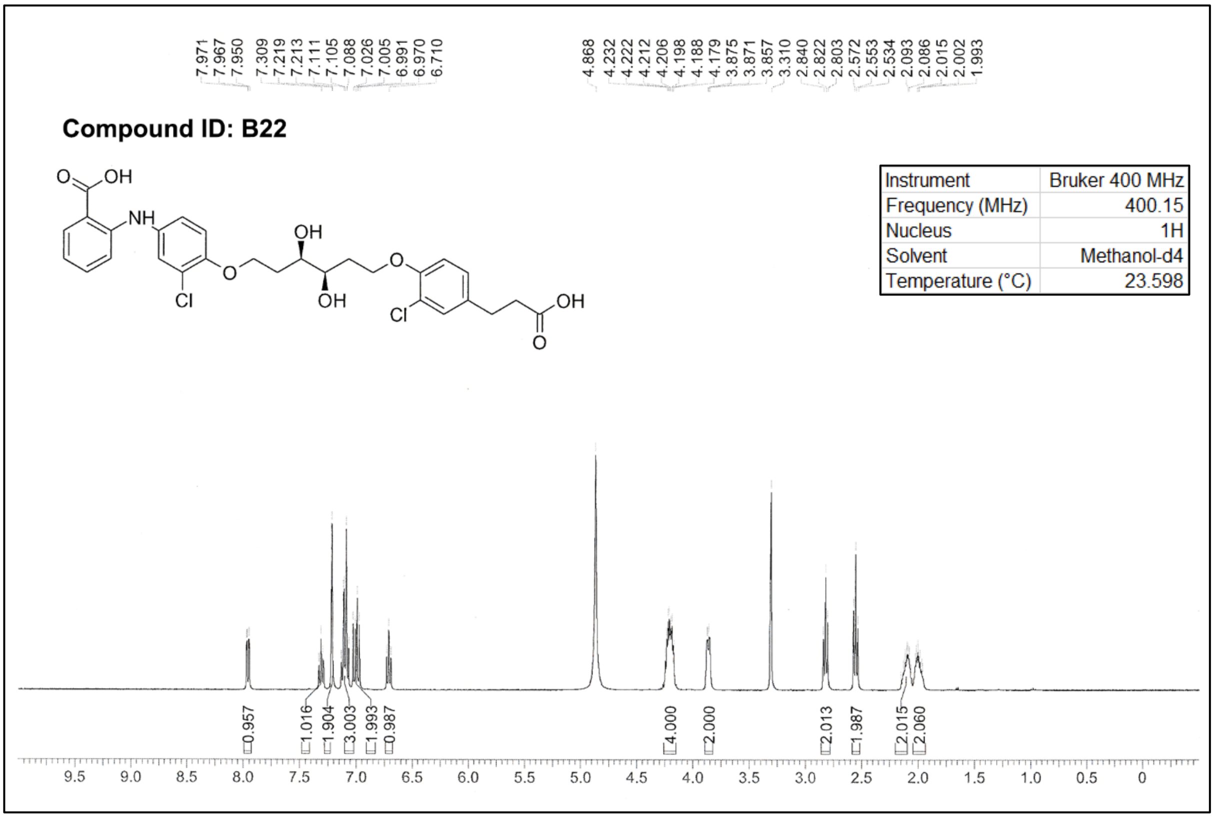

Compound ID: B22

Instrument	Bruker 400 MHz
Frequency (MHz)	400.15
Nucleus	1H
Solvent	Methanol-d4
Temperature (°C)	23.598

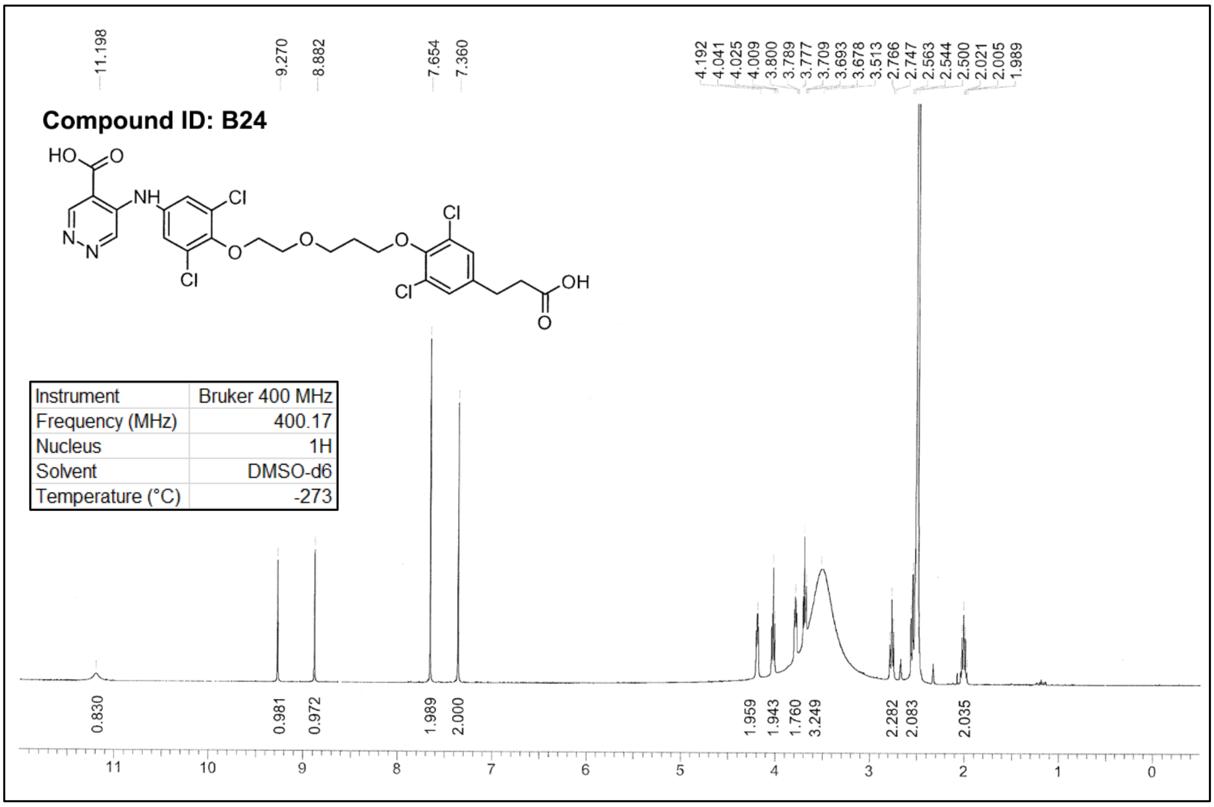

Compound ID: B24

Instrument	Bruker 400 MHz
Frequency (MHz)	400.17
Nucleus	1H
Solvent	DMSO-d6
Temperature (°C)	-273

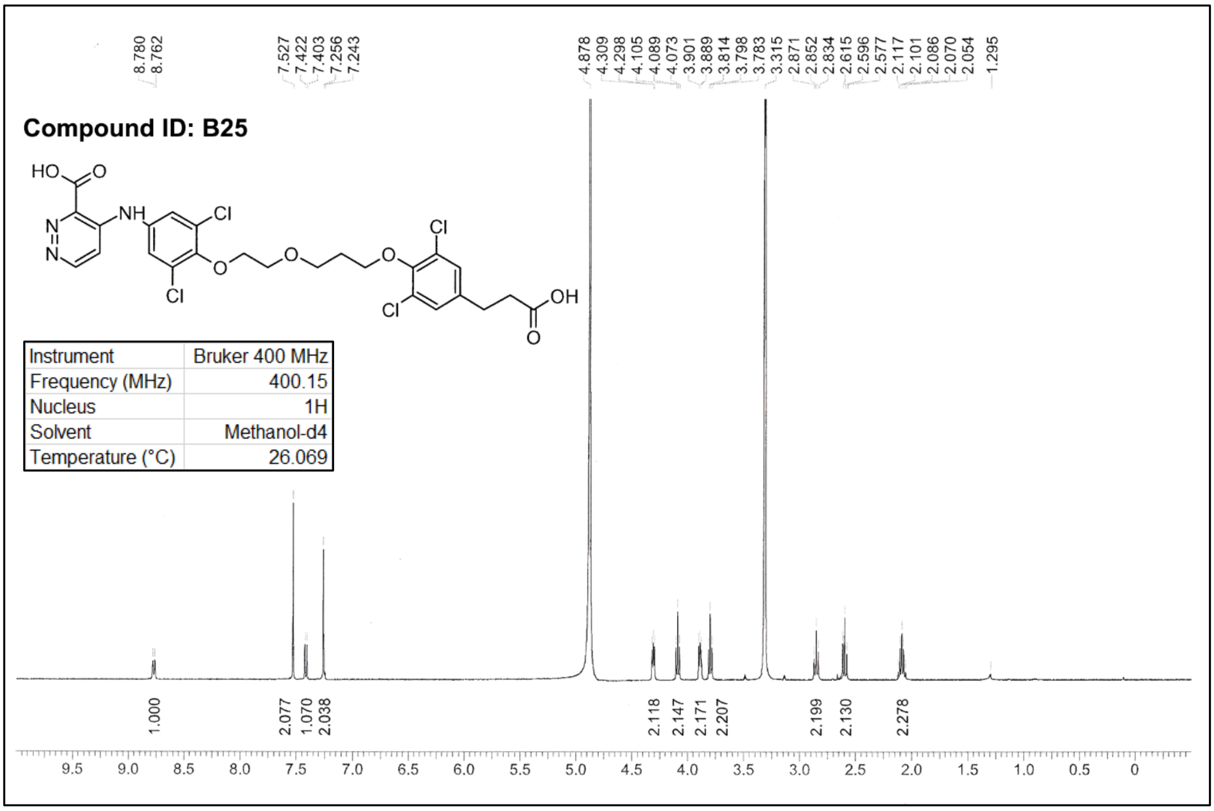

Compound ID: B25
Instrument | Bruker 400 MHz
Frequency (MHz) | 400.15
Nucleus | 1H
Solvent | Methanol-d4
Temperature (°C) | 26.069

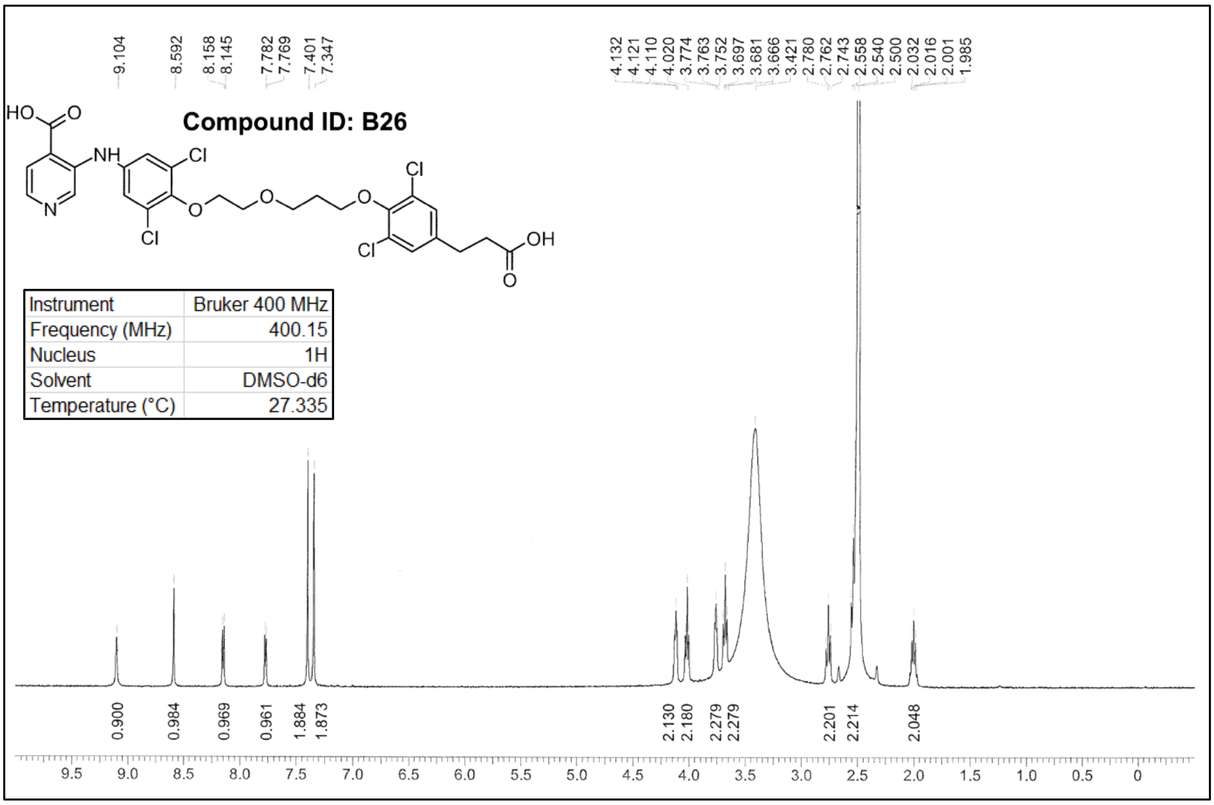

Compound ID: B26
Instrument | Bruker 400 MHz
Frequency (MHz) | 400.15
Nucleus | 1H
Solvent | DMSO-d6
Temperature (°C) | 27.335

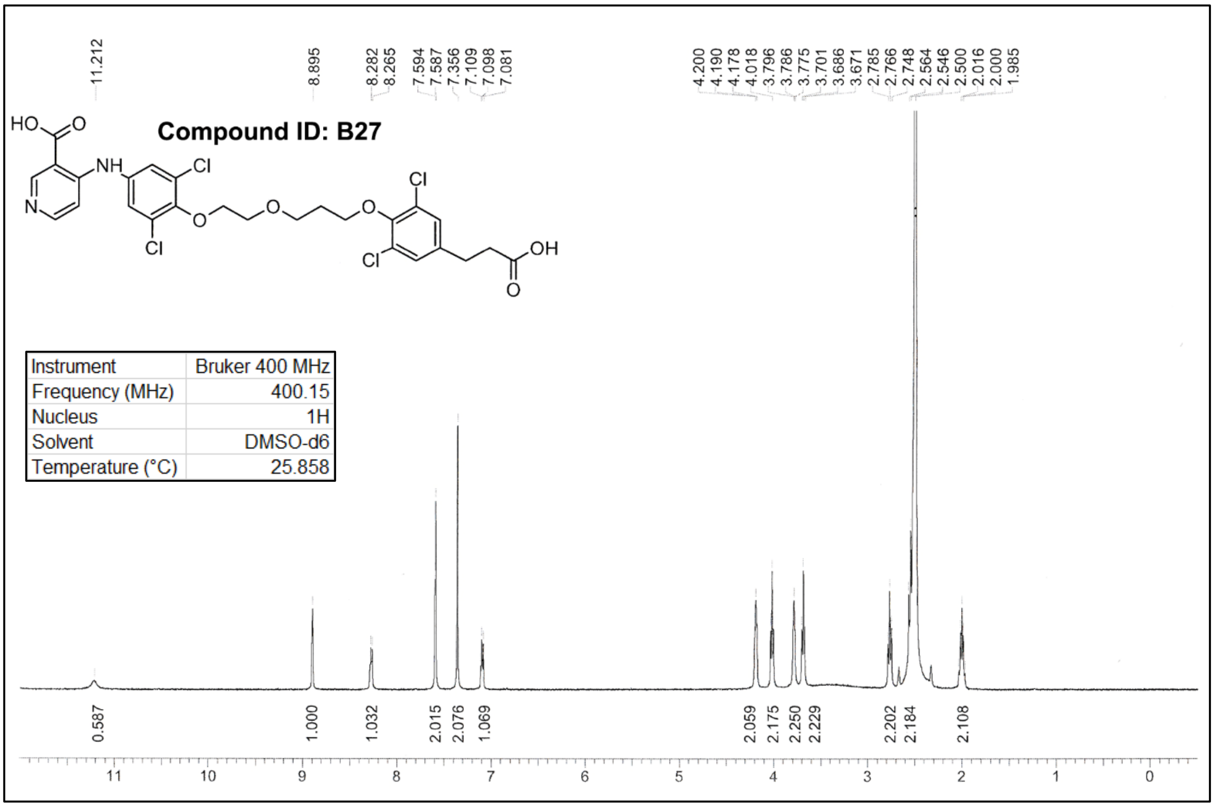
Compound ID: B27
HO
O
NH
N
Cl
Cl
O
O
O
Cl
Cl
OH
O
Instrument Bruker 400 MHz
Frequency (MHz) 400.15
Nucleus 1H
Solvent DMSO-d6
Temperature (°C) 25.858
11.212
8.895
8.282
8.265
7.594
7.587
7.356
7.109
7.098
7.081
4.200
4.190
4.178
4.018
3.796
3.786
3.775
3.701
3.686
3.671
2.785
2.766
2.748
2.564
2.546
2.500
2.016
2.000
1.985
0.587
1.000
1.032
2.015
2.076
1.069
2.059
2.175
2.250
2.229
2.202
2.184
2.108
11
10
9
8
7
6
5
4
3
2
1
0
